# Synthesis and Performance of MOF-Based Non-Noble Metal Catalysts for the Oxygen Reduction Reaction in Proton-Exchange Membrane Fuel Cells: A Review

**DOI:** 10.3390/nano10101947

**Published:** 2020-09-30

**Authors:** Nicolas Delaporte, Etienne Rivard, Sadesh K. Natarajan, Pierre Benard, Michel L. Trudeau, Karim Zaghib

**Affiliations:** 1 Hydro-Québec, Center of Excellence in Transportation Electrification and Energy Storage, Varennes, QC J0L 1N0, Canada; rivard.etienne@hydroquebec.com; 2 Université du Québec à Trois-Rivières (UQTR), Hydrogen Research Institute, Trois-Rivières, QC G9A 5H7, Canada; sadesh@irh.ca (S.K.N.); Pierre.Benard@uqtr.ca (P.B.); 3 Department of Materials Engineering, McGill University, 3610 University, Room 2140, Montreal, QC H3A 0C5, Canada; karim.zaghib@mcgill.ca

**Keywords:** catalysts, oxygen reduction, nanomaterials, fuel cells

## Abstract

Hydrogen is widely regarded as a prime energy carrier for bridging the gap between renewable energy supply and demand. As the energy-generating component of the hydrogen cycle, affordable and reliable fuel cells are of key importance to the growth of the hydrogen economy. However, the use of scarce and costly Pt as an electrocatalyst for the oxygen reduction reaction (ORR) remains an issue to be addressed, and in this regard, metal–organic frameworks (MOFs) are viewed as promising non-noble alternatives because of their self-assembly capability and tunable properties. Herein, recent (2018–2020) works on MOF-based electrocatalysts containing N-doped C, Mn, Fe, Co, multiple metals, and multiple sites are reviewed and summarized with a focus on ORR activity, and the principal physicochemical properties and electrochemical performance of these catalysts realized using rotating electrodes are compared.

## 1. Introduction

Globally, several actors are striving to foster and accelerate a shift away from fossil energy [1] for several reasons, with the major motivating factors being climate change mitigation [2], energy independence [3], and a cleaner environment [4]. In this regard, intermittent renewable energy sources (IRES; e.g., wind, solar, and tidal power) are generally safe and, therefore, interesting candidates. Conversely, nuclear technologies can provide energy in copious amounts [5] but are plagued by safety concerns and waste management issues [6]. The efficient use of IRES requires the development of energy storage systems [7], as exemplified by the field of transportation, e.g., vehicles cannot be practically powered by IRES directly. Among the numerous energy storage solutions, batteries, which are especially attractive in some areas [8], suffer from the drawbacks of low energy density and high cost [9], while mechanical energy storage systems are not generally practical despite being very simple and efficient [10]. The power-to-gas technology, defined as the implementation of water electrolysis or its combination with methanation [11], has recently attracted increased attention, offering attractive energy densities and a cost-lowering potential for some applications, which could induce a shift to a hydrogen economy in the near future [12,13,14]. Indeed, hydrogen has been long viewed as an ideal energy carrier because of its very high energy density of 120–142 MJ/kg, which is about three times that of gasoline.

Hydrogen can be converted to energy in several ways [15,16,17], with fuel cells currently being dominant in the case of transportation applications. In fact, fuel cell shipment volumes are rapidly increasing, e.g., nearly 70,000 units (≈800 MW) were shipped in 2018, with the corresponding combined revenue reaching nearly $2.3 billion [18]. The currently market-dominating fuel cells such as proton-exchange membrane fuel cells (PEMFCs) [19] are operated at low temperatures and require rare and expensive catalyst materials [20,21], the amount of which in the fuel cell systems of mid-size cars such as Toyota Mirai can exceed 30 g [22]. Today, the fuel cell cost is estimated at around $210/kW, but it is expected to decrease to ≈$45 /kW with the development of modern technologies, given that the high production volume target of 500 k per year is achieved. At $45/kW, the overall cost of the power system (fuel cell system and hydrogen tank) would be less than half the estimated cost of the present Tesla model S battery system (at $217/kWh) [23]. However, when the cost of energy is considered (electricity vs. hydrogen), it is quite probable that batteries will remain the dominant energy storage method for low-daily-mileage applications. For other (more intensive) applications such as fleet (e.g., taxi), buses, and trucks, hydrogen and fuel cell systems are viewed as the best combination to replace fossil fuels.

**Figure 1:**
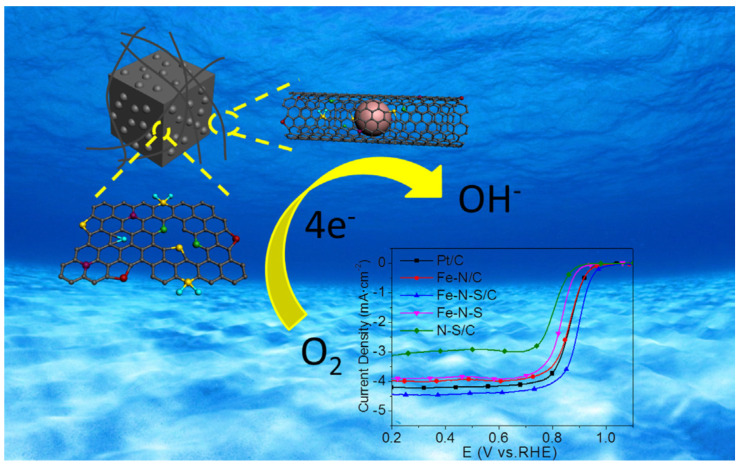
Illustration representing an hybrid of Fe_3_C@N,S co-doped carbon nanotubes coated porous carbon as a superior catalyst toward oxygen reduction reaction (ORR) for fuel cell. In insert: typical ORR polarization curve for various Fe-containing carbonaceous and Pt/C catalysts. Reprinted with permission from ref [26]. Copyright (2019) Elsevier.

The use of scarce and costly Pt as a noble electrocatalyst to overcome the sluggish kinetics and high overpotential of the oxygen reduction reaction (ORR) at the cathode remains one of the issues to be addressed [24]. Indeed, the ultimate Department of Energy (DOE) goal of $30/kW relies on the development of new Pt-group metal (PGM)-free catalysts [18]. For these reasons, non-noble catalysts, mostly based on porous carbon substrates with a large surface area and hierarchical porosity [25] to permit the efficient diffusion of electrolytes and gases to the active (e.g., non-noble metal) sites, are intensely studied (see Figure 1 for an example of carbonaceous catalyst composed of encapsulated metallic nanoparticles and used as the cathodic active material of a fuel cell for the reduction of O_2_ in OH^−^ in alkaline media) [26]. However, the low active site density of doped carbon catalysts requires the use of large carbon amounts to compete with Pt electrocatalysts. In fact, the usual doped-carbon catalyst loading (≈4 mg cm^−2^) significantly exceeds that of Pt/C (0.2–0.8 mg cm^−2^), which makes water and heat management in the former fuel cells more difficult [27]. Therefore, current research should focus on the development of materials with high active site density, large surface area, and optimal porous structures to boost the practical applications of non-noble electrocatalysts.

We encourage researchers to consult a recent review, titled “Efficient Oxygen Reduction Catalysts of Porous Carbon Nanostructures Decorated with Transition Metal Species” [28] Although non-noble catalysts may or may not contain metals, the above review focuses on transition metal-based electrocatalysts, as they are gaining more traction than their metal-free counterparts. The methods used to prepare metal-decorated porous carbons can be divided into non-atomically and atomically dispersed ones. The former methods include self-templated techniques, mixture processes, and multiprocesses, while the latter are categorized as templated, self-templated, mixture processes, wet chemical processes, and others. Among these methods, techniques relying on the preparation of metal–organic frameworks (MOFs) [29], a class of self-assembled materials synthesized by the complexation of metal ions (e.g., Co^2+^) by organic linkers/ligands such as 1,3,5-benzenetricarboxylic acid or 4,4′-bipyridine, are widely employed [30]. In the present review, the current state of research on MOF-based electrocatalysts for the ORR is summarized.

## 2. Methodology

In an ideal case, the ORR performance of a given catalyst should normally be tested in a practical fuel cell fed with air/O_2_ and H_2_ using a membrane electrode assembly (MEA). However, the comparison of different electrocatalysts is challenged by the abundance of parameters that can impact electrochemical performance, e.g., catalyst loading, the method used to prepare the catalyst ink (solvents, mixing method, etc.), nature of the proton-exchange membrane (e.g., Nafion), or catalyst layer fabrication method [31]. In addition, if the study focuses only on ORR performance evaluation, the cathode, where the reduction of oxygen to H_2_O takes place, has to be tested independently, preferably by rotating disk electrode (RDE) and rotating ring-disk electrode (RRDE) measurements. In this case, a thin catalyst layer is deposited on the surface of a polished rotating electrode (usually made of glassy carbon or Pt) as the working electrode of a three-electrode assembly immersed into an O_2_-saturated acidic or alkaline electrolyte [32]. Thus, in the present review, we report, where possible, the electrochemical parameters obtained for each catalyst and derived from RDE/RRDE experiments.

**Figure 2:**
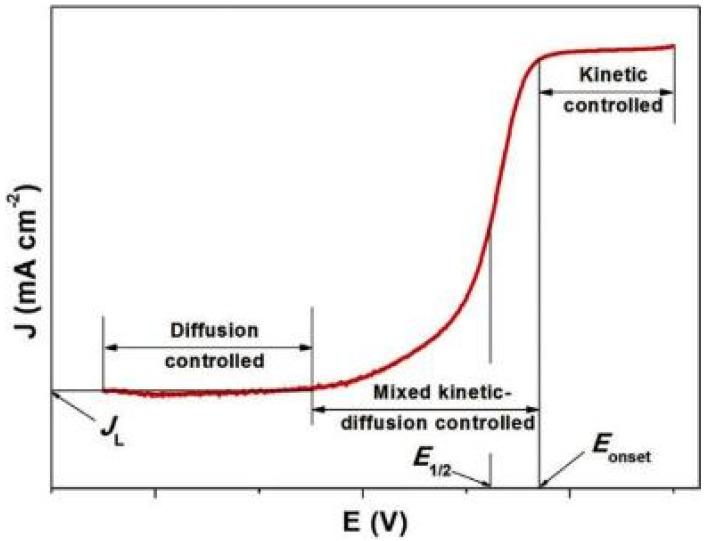
Typical ORR polarization curve showing the definitions of the onset potential (*E*_onset_), half-wave potential (*E*_1/2_), and diffusion-limited current density (*J*_L_). Reprinted with permission from ref. [33]. Copyright (2015) John Wiley and Sons.

Figure 2 shows a typical ORR polarization curve obtained for the working electrode with a standard catalyst [33], revealing three parts corresponding to different ORR steps. The first part, in which the ORR is quite slow and the current density slightly increases with decreasing potential, is the kinetically controlled area. In the intermediate area, corresponding to a mixture of kinetic and diffusion control, the current suddenly increases with decreasing potential, reflecting the onset of oxygen reduction. Finally, the diffusion-controlled area is characterized by a plateau that allows one to determine the diffusion-limited current density (*J*_L_), which strongly depends on the electrode rotation speed and is related to the rate of reactant diffusion through the catalyst layer. Electrocatalysts are also characterized by their onset potential (*E*_onset_) and half-wave potential (*E*_1/2_), with more positive values generally corresponding to more active catalysts. *E*_onset_ is generally determined at a current density of 0.1 mA cm^−2^ [34]. The electron transfer number (*n*) and H_2_O_2_ yield are also important parameters that have to be considered and are closely related. For the cathode, the energy yield is maximized for a four-electron reduction pathway, resulting in the conversion of oxygen to water. However, for inefficient catalysts, a two-electron pathway leading to the production of H_2_O_2_ can be preferred, which dramatically reduces fuel cell performance and can harm the MEA and carbon-based catalysts. The electron transfer number can be determined from RDE experiments using the Koutecky–Levich (K-L) equation:

1/*J* = 1/*J*_k_ + 1/*J*_d_ (1)

where *J*, *J*_k_, and *J*_d_ are the total current density, kinetic current density, and diffusion-limited current density, respectively. *J*_d_ depends on the electrode rotation speed and can be expressed as

*J*_d_ = *Bω*^1/2^ (2)

where *B* = 0.62*nFC*_0_(*D*_0_)^2/3^*υ*^−1/6^. In this expression, *F* is the Faraday constant (96485 C mol^−1^), *C*_0_ is the bulk oxygen concentration, *D*_0_ is the oxygen diffusion coefficient in the electrolyte, *υ* is the kinetic viscosity of the electrolyte, and *ω* is the angular velocity.

The H_2_O_2_ yield (%) and *n* can be also calculated from RRDE measurements as

(3) $$n=\frac{4I_{D}}{I_{D}+I_{R}/N}$$

(4) $$H_{2}O_{2}\%=\frac{200I_{R}/N}{I_{D}+I_{R}/N}$$

where *I_D_* and *I_R_* are the disk and ring currents, respectively, recorded during RRDE tests, and *N* is the current collection efficiency of the ring.

Moreover, catalyst durability, which can be calculated in two ways, is also an important parameter. In chronoamperometric measurements, the current density at a fixed potential is recorded for a certain time, and the percentage corresponding to the retention of the initial current density is calculated. In the second method, several thousands of cyclic voltammetry (CV) cycles are performed at a relatively high scan rate (generally 50–150 mV s^−1^) within a predefined potential window, and the *E*_1/2_ value observed after the CV experiment is compared to the initial value.

Finally, in addition to electrochemical parameters, some crucial physical properties such as the Brunauer–Emmett–Teller (BET) surface area and metal (M) and nitrogen atomic contents have to be considered. In fact, a high content of atomically dispersed M-N*_x_* sites and a high BET surface area, facilitating the contact between the electrolyte and the active sites, are mandatory to obtain a good electrocatalyst.

## 3. MOF-Based ORR Electrocatalysts

MOFs are characterized by a well-ordered crystalline structure, high porosity, excellent structural flexibility, and the easy incorporation of tunable functionalities through the choice of an appropriate linker [35]. The utilization of MOFs (especially that of porous carbon materials obtained by MOF calcination) for hydrogen storage was first reported in 2008 by Liu et al. [36]. Thereafter, numerous examples of MOF pyrolysis have been reported to yield nanostructured carbon-based materials with various pores sizes, specific surface areas, and morphologies [37]. After carbonization, the organic ligands are transformed into a porous graphitic carbon matrix, while the metal ions are converted into metal or alloy nanoparticles to afford efficient electrocatalysts for PEMFCs [38]. The thermal decomposition of MOFs offers several advantages [39]. Well-controlled porosity allows one to facilitate mass transport and thus optimize the electrocatalytic reactions, while the good dispersion of metal centers in the carbon-based material improves catalyst utilization efficiency. The incorporation of heteroatoms such as N [38], S [40], B [41], F [42], or P [43] into ligands yields doped carbon–based electrocatalysts with superior ORR activity. For instance, Co-N*_x_*-C [44] and most importantly, Fe-N*_x_*-C [45,46] structured ORR electrocatalysts are known to be very efficient. The results of previous studies indicate that the ORR performance of M/N-C (M = metal) catalysts usually depends on the metal and decreases in the order of Fe > Co > Mn > Cu > Ni [47,48]. In addition, the encapsulation of precursors in the pores of freshly assembled MOFs allows one to achieve a perfect dispersion of single metal atoms and heteroatoms after pyrolysis. For example, Li et al. fabricated an isolated single-atom Fe-N-doped porous carbon by the pyrolysis of a zeolitic imidazolate framework 8 (ZIF-8) with an Fe source encapsulated in its pores [49]. 

Herein, because of the large amount of papers and reviews focusing on the fabrication of non-noble electrocatalysts for fuel cells through the thermal decomposition of MOFs, we solely review works reported since 2018. The related studies are categorized according to the type of catalytically active sites, i.e., N-doped carbons, Fe-, Mn-, and Co-based catalysts, as well as multi-metal (more than one metal such as Ce, Ni, Fe, Cu, Zn, or Co is involved in the ORR) and multi-site (different sites are identified, corresponding to metallic species, organic species, or a mixture of them) electrocatalysts. Notably, almost all materials detailed below are N-doped carbonaceous electrocatalysts, although in some cases, the MOFs contained no transition metals (except for Zn, which was evaporated), and hence, the only catalytic sites in these cases corresponded to the nitrogen of N-doped carbons, e.g., pyridinic N, pyrrolic N, and graphitic N, which are known to efficiently catalyze O_2_ reduction [50]. Table 1, Table 2, Table 3, Table 4, Table 5 and Table 6 list the principal characteristics and electrochemical performance of these ORR catalysts. It is worth noting that although some MOF-based ORR electrocatalysts have been recently reported, they still contain small quantities of noble metals and are consequently not discussed here [51,52,53,54,55]. 

### 3.1. N-Doped Carbons

Huang et al. used pre-designed ZnO@ZIF-8 core–shell microparticles as self-sacrificial templates to prepare hollow N-doped carbon microspheres (HNCSs) [56]. ZnO microspheres were uniformly coated by ZIF-8 nanoparticles (≈100 nm) to generate core–shell structures with a diameter of 1–2 µm. The hollow structure of the carbonaceous HNCS framework was confirmed by transmission electron microscopy (TEM) imaging after pyrolysis. N_2_ adsorption–desorption curves revealed that meso-/macropores were much more abundant than micropores, which resulted in a low Brunauer–Emmet–-Teller (BET) surface area of 502 m^2^ g^−1^. However, hierarchically porous HNCSs possessed a large total pore volume with a *V*_meso + macro_/*V*_micro_ volume ratio of 39.4, therefore offering a great capacity for mass transfer during the ORR. HNCSs exhibited a high electrocatalytic activity for the ORR, featuring *E*_onset_ = 0.92 V, *E*_1/2_ = 0.864 V, and *J*_L_ = 5.34 mA cm^−2^, which were close to the respective values for Pt/C (0.97 V, 0.83 V, and 5.30 mA cm^−2^). Moreover, HNCSs featured *n* = 3.86–3.98 and peroxide yields as low as 4.28–5.03% between 0.3 and 0.7 V. Finally, the above electrocatalyst also presented a high current retention of 96.5% after 10-h testing, whereas a value of 90% was obtained for Pt/C under identical conditions.

Huang et al. reported the effect of amino acid modulation on the growth of ZIF particles, using L-aspartic acid as a crystal and structure-directing agent [57]. Pyrolysis of the amino-functionalized ZIF-8 yielded N-doped carbon (A-Z-1000). The X-ray diffraction (XRD) pattern of the amino-modified ZIF showed typical peaks in accordance with the simulated pattern of ZIF-8, except for the absence of a peak at 7°, revealing the occurrence of amino acid-assisted oriented crystal growth for ZIF-8. The pyrolyzed MOF exhibited a flower-like structure with a BET specific surface area of 484 m^2^ g^−1^. X-ray photoelectron spectroscopy (XPS) showed that the incorporation of L-aspartic acid, which acted as a secondary nitrogen source, increased the atomic content of nitrogen in N-doped carbon (4.08 at%) compared to that of ZIF-8 synthesized following the same procedure without the amino acid (3.28 at%). When the CV profiles of A-Z-1000 were recorded under identical conditions in N_2_- and O_2_-saturated 0.1 M KOH as an electrolyte, a well-defined cathodic peak was observed only in the O_2_-saturated solution, which demonstrated that the investigated carbon exhibited obvious ORR activity. A-Z-1000 featured an onset potential of 0.87 V and a limiting current density of 4.25 mA cm^−2^, which exceeded the values of the control sample prepared without the amino acid (0.81 V, 3.5 mA cm^−2^). However, the *n* values of A-Z-1000 obtained from K-L plots at various potentials (*n* = 2.70–3.04) indicated the occurrence of both two- and four-electron ORR pathways. Consequently, the peroxide yield reached ≈43% in the 0.0–0.7 V potential window. Unfortunately, long-term stability and methanol tolerance tests were not performed in this work, which makes us doubt the practical applicability of this catalyst for the ORR.

A hierarchical nanoporous N-doped carbon was prepared by the facile pyrolysis of a well-designed nanosized ZIF-8 precursor (synthesized in ethanol using 2-methylimidazole as a ligand and Zn^2+^ as a metal center) under optimal conditions [58]. After pyrolysis at 1000 °C, the organic ligand of ZIF-8 was transformed into N-doped nanoporous carbon with a uniform particle size of ≈45 nm. XPS showed that this catalyst, denoted as ZNC-1000, contained ≈54.7 at% pyridinic and graphitic nitrogens, which are known to be effective for ORR electrocatalysis. Moreover, the catalyst had a significant BET surface area of 1205 m^2^ g^−1^ and a micropore surface area of 1035 m^2^ g^−1^, offering efficient access to catalytically active sites. ZNC-1000 featured *E*_onset_ = 0.915 V, *E*_1/2_ = 0.813 V, and a high *J*_L_ of 5.599 mA cm^−2^, which exceeded the values obtained for Pt/C (0.909 V, 0.812 V, and 4.032 mA cm^−2^, respectively). The peroxide yield was calculated as 7.1% (13.2% for Pt/C), corresponding to *n* = 3.86 (3.74% for Pt/C). Finally, catalyst stability was tested by performing 5000 CV scans, and no variance in *E*_onset_ was observed. In addition, after 30,000 s of chronoamperometric testing, ZNC-1000 featured a current retention of 87.5%, whereas a smaller value of 40.2% was obtained for Pt/C under identical conditions.

Another catalyst was synthesized by the pyrolysis of an in situ formed hybrid of MIL-101(Fe) (30 wt %)) and polypyrrole (PPy) nanotubes at 800 °C under nitrogen [59]. The obtained catalyst, denoted as 30%MIL/PPy-800, comprised a hierarchically porous material wrapped around carbon nanotubes and, according to XPS results, contained pyridinic N (≥46.1 at%), which is one of the most crucial active sites in N-doped ORR catalysts. However, no Fe was detected by XPS, and the content of this metal was not probed by other methods such as inductively coupled plasma (ICP). Thus, the above catalyst was categorized as N-doped carbon. The *E*_onset_ and *E*_1/2_ of 30%MIL/PPy-800 equaled 17 and −116 mV, respectively, being 4 and 12 mV more positive than those of Pt/C. Moreover, *n* = 3.7–3.9 and a peroxide yield of <15% (exceeding the values reported in Table 1 for other N-doped carbonaceous electrocatalysts) in a wide potential range were obtained. The long-term stability of Pt/C and 30%MIL/PPy-800 was tested by continuous CV scanning at 100 mV s^−1^ over 10,000 cycles. For the latter catalyst, only a slight decrease in limiting current density was observed without an apparent negative shift of onset/half-wave potentials. In contrast, the onset/half-wave potentials and the *J*_L_ of the benchmark Pt/C catalyst dramatically dropped, demonstrating the superior stability of 30%MIL/PPy-800.

**Figure 3:**
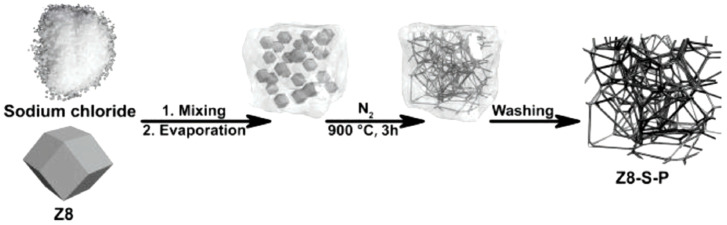
Schematic synthesis of an interconnected hierarchically porous carbon catalyst (Z8-S-P) by pyrolysis of an NaCl/ZIF-8 (zeolitic imidazolate framework 8) composite. Reprinted with permission from ref. [60]. Copyright (2018) John Wiley and Sons.

Zhao et al. reported a simple synthesis of interconnected carbon structures based on the pyrolysis of NaCl/ZIF-8 composites, with the general procedure presented in Figure 3 [60]. During pyrolysis, molten NaCl activated the surface of ZIF-8 particles and connected them into carbon fibers. Moreover, subsequent NaCl removal led to the formation of macropores ideal for fast mass transfer. Scanning electron microscopy (SEM) analyses revealed the web-like morphology of the catalyst (denoted as Z8-S-P) and the presence of abundant meso- and macropores (20–80 nm). ICP analysis confirmed that Zn was present as trace levels (<0.005 wt %), and ORR activity was therefore mainly attributed to N-containing sites. Z8-S-P presented a BET surface area of 1086 m^2^ g^−1^ and a high total pore volume of 2.0 cm^3^ g^−1^. In 0.1 M KOH, this new catalyst did not outperform the benchmark Pt/C catalyst, featuring *E*_onset_ = 0.964 V and *E*_1/2_ = 0.862 V, while the respective values of Pt/C reached 0.979 and 0.834 V. The electron transfer number of Z8-S-P (≈3.95) was also lower than that of Pt/C (≈3.99), while the peroxide yield of the former (<5%) was higher than that of the latter but still low. After a 10,000-cycle accelerated degradation test carried out to check catalyst durability, the *E*_1/2_ of Pt/C shifted to more negative values by 27 mV, whereas a two-fold lower shift in the same direction was observed for the MOF-based catalyst.

N-doped graphene-modified MOF catalyst was synthesized using a planetary ball milling method by grinding N-functionalized graphene (N-G, prepared by mixing graphene oxide and melamine for several hours) and ZIF-8 under optimized conditions without any heat treatment [61]. The obtained catalyst (N-G/MOF-350) presented both amorphous and crystalline structures inherited from ZIF-8 and N-G precursors, featuring a broad particle size distribution with an average of 955 nm and a fairly large BET surface area of 1039.8 m^2^ g^−1^, although the total pore volume remained low (≈0.42 cm^3^ g^−1^). In our opinion, the electrochemical performance of the above catalyst was not fully investigated, although catalyst stability was tested by performing 2000 CV cycles in an O_2_-saturated electrolyte. The shape of the linear sweep voltammetry (LSV) curve obtained after 2000 cycles was compared to that of the curve recorded before cycling in an O_2_-saturated electrolyte. The catalyst retained only 58.2% of its initial peak current density, which indicated inferior stability in comparison with that of other examples reported in Table 1; moreover, no comparison with the benchmark Pt/C catalyst was provided. However, N-G/MOF-350 exhibited high selectivity and strong tolerance toward methanol oxidation, while Pt/C showed a >50% spontaneous performance loss upon methanol addition.

**Figure 4:**
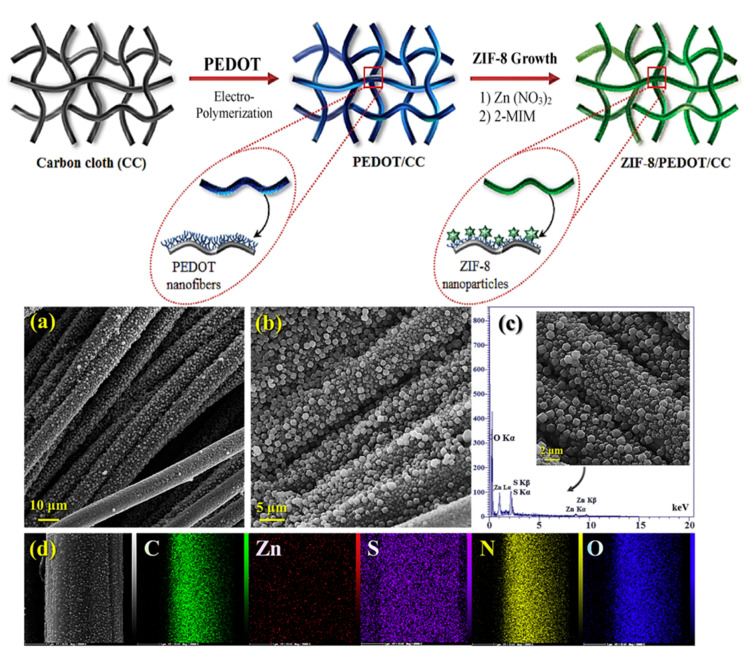
(**a**) Schematic representation of successive carbon cloth (CC) modification with a poly(3,4-ethylenedioxythiophene) (PEDOT) film and ZIF-8 nanoparticles. (**b**) Low- and (**c**) high-magnification SEM images and (**d**) EDX spectrum of ZIF-8 nanoparticles deposited on PEDOT-coated carbon fibers. (**e**) Elemental distributions of C, Zn, S, N, and O obtained for ZIF-8/PEDOT/CC. Reprinted and modified with permission from ref. [62]. Copyright (2020) Elsevier.

In 2020, Asadian et al. reported a material that could be the precursor of an N-doped carbon catalyst [62], proposing a cost-effective and metal-free catalyst based on ZIF-8 nanoparticles/electropolymerized poly(3,4-ethylenedioxythiophene) (PEDOT) film on the surface of a flexible carbon cloth (CC) electrode. The PEDOT film was electrodeposited on the CC surface via a pulse potentiostatic electropolymerization method in an aqueous electrolyte, and then, ZIF-8 nanoparticles were grown on the surface of PEDOT-coated carbon fibers (Figure 4a). The catalyst, denoted as ZIF-8/PEDOT/CC, was used without additional heat treatment. SEM imaging showed that carbon fibers were homogeneously covered by a uniform PEDOT film composed of worm-like nanofibers, and after 12 h of crystal formation, the carbon fibers were covered with polygonal ZIF-8 nanoparticles (Figure 4b–d). The authors optimized the PEDOT deposition time, revealing that the PEDOT-coated CC electrode fabricated using 50 consecutive potentiostatic cycles featured the best electrochemical performance. The presence of the conductive PEDOT film led to an increase in electrical conductivity, as expected. Unfortunately, the nitrogen content of the resulting material was not quantified, and the BET surface area was not provided. Electrochemical tests were conducted in 0.1 M KOH as an electrolyte, and *E*_onset_ values of 0.78 and 0.70 V were obtained for ZIF-8/PEDOT/CC and the bare CC electrode, respectively. The peroxide yield was not calculated, while *n* = 3.5–3.9 (depending on the potential) was obtained. The long-term stability of Pt/C and ZIF-8/PEDOT/CC was tested by applying a constant potential of 0.2 V for 10,000 s. At the end of the test, ZIF-8/PEDOT/CC retained 80% of the initial current density, whereas Pt/C retained only 60% of the steady-state current, which revealed the superior durability of the former catalyst.

**Table 1:** Recent research works (2018–2020) dealing with the preparation of N-doped carbonaceous electrocatalysts for fuel cells with a focus on ORR activity. Examples in alkaline media are provided, and major physicochemical properties and electrochemical performances realized using rotating electrodes are compared.

				RDE/RRDE Experiments ***					
Precursors	Main Types of Catalytically Active Sites	BET Surface Area (m^2^ g^−1^)	N/M (N = Nitrogen; M = Metal) Contents (at%) *	*E*_1/2_ (V vs. RHE) **	Average n	Peroxide Yield	Cyclability	Methanol Tolerance	Reference
ZnO@ZIF-8	N-doped carbon	502	1.92/none	0.82 (5 mV s^−1^)	3.86	4.28–5.03% (0.3–0.7 V)	After 10 h, 96.5% of initial current retained at 0.6 V	Excellent under alkaline conditions	[56]
ZIF-8 + L-aspartic acid	N-doped carbon	484	4.08/none	0.87 (onset potential)	2.7–3.04	≈43% (0.0–0.7 V)	n/a	n/a	[57]
ZIF-8	N-doped carbon	1205	4.72/0.44(Zn)	0.813	3.86	7.1% (0.2–0.8 V)	After 8.3 h, 87.5% of initial current retained at −0.5 V vs. Ag/AgCl	Excellent under alkaline conditions	[58]
MIL-101(Fe) + PPy nanotubes	N-doped carbon (no proofs for Fe-N_x_)	892	5.04/n/a	−0.116 vs. Ag/AgCl	3.7–3.9	<15% (−0.1 to −0.8 V vs. Ag/AgCl)	Stability experiments realized by CV (100 mV s^−1^, −0.8 to 0.2 V vs. Ag/AgCl, 10000 cycles): no variance in *E*_1/2_	Excellent under alkaline conditions	[59]
ZIF-8 + NaCl	N-doped carbon	1086	5.72/none	0.964	≈3.95	<5% (0.2–0.8 V)	Stability experiments realized by CV (50 mV s^−1^, −0.4 to 0.0 V, 10000 cycles, 400 rpm): negative *E*_1/2_ shift of ≈13 mV	n/a	[60]
ZIF-8 + N-doped graphene	N-doped carbon	1039.8	n/a	n/a	n/a	n/a	Stability experiments realized by CV (150 mV s^−1^, 0.3–1.1 V, 2000 cycles): 58.2% of initial current retained	Excellent under alkaline conditions	[61]
ZIF-8 + PEDOT	Precursor of N-doped carbon	n/a	n/a	0.78 (onset potential, no electrode rotation, 20 mV s^−1^)	3.5–3.9 (0.2–0.3 V)	n/a	After 2.8 h, 80% of initial current retained at 0.2 V	Excellent under alkaline conditions	[62]

* Sometimes, weight percentage was used, and hence, “wt %” data are provided. More than one metal or heteroatom can be provided depending on the active sites involved. ** Potentials are referenced to RHE except when stated otherwise. The ORR performance values were evaluated at 1600 rpm and 10 mV s^−1^ except when stated otherwise. *** All the ORR performance values listed were measured in 0.1 M KOH.

### 3.2. Mn-Based Electrocatalysts

**Figure 5:**
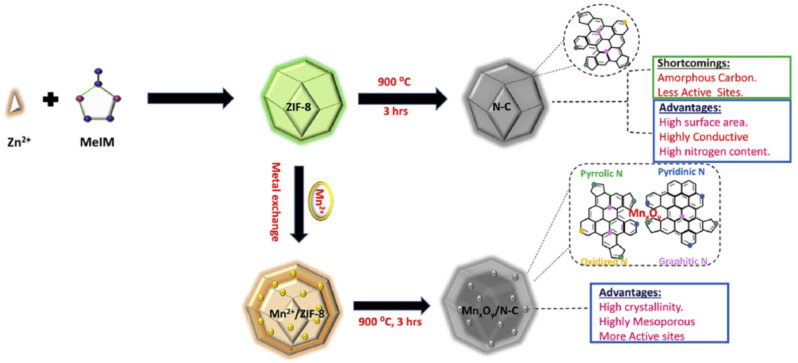
Schematic synthesis of a hybrid Mn*_x_*O*_y_*/N-C catalyst based on post-synthetic cation exchange. Reprinted with permission from ref. [63]. Copyright (2018) Elsevier.

One of the first hybrid Mn*_x_*O*_y_*/N-C catalysts was prepared by Ahmad Shah et al. by the immobilization of Mn^2+^ ions into a ZIF-8 structure through Mn-N coordination with imidazole [63] (see Figure 5 for the synthetic procedure) followed by the exchange of Zn^2+^ in ZIF-8 polyhedra for Mn^2+^ via the simple soaking of ZIF-8 in a methanolic solution of Mn(NO_3_)_2_. The structure (polyhedra of ≈150 nm) was not affected by this cation exchange even when ≈13% of Zn^2+^ in the MOF structure was successfully exchanged for Mn^2+^. The catalyst produced by thermal treatment under N_2_ well inherited the overall polyhedral morphology, while its surface underwent slight shrinkage and became concave. The resulting material presented a BET surface area of 1497 m^2^ g^−1^, and high-resolution TEM images showed that the graphitic carbon matrix contained white dots identified as Mn_3_O_4_ and MnO_2_ nanoparticles by XRD. The electrocatalytic activity of this catalyst was measured in 0.1 M KOH, and the onset potential (0.987 V) was found to be almost identical to that of Pt/C, whereas the half-wave potential (0.871 V) exceeded that of Pt/C (0.854 V). RRDE experiments showed that the H_2_O_2_ yield of the novel catalyst remained below 2% in the potential window of 0.20–0.80 V, while the average *n* of ≈3.75 indicated high selectivity for the four-electron reaction. Catalyst stability was probed by CV cycling between 0.6 and 1.2 V at a sweep rate of 50 mV s^−1^ for 8000 cycles. At the end of the experiment, *E*_1/2_ negatively shifted by 2 mV, whereas the corresponding shift observed for the benchmark Pt/C catalyst equaled 35 mV, which indicated the superior stability of the Mn*_x_*O*_y_*/N-C catalyst.

Benzene tricarboxylic acid (BTC)-based MOFs were synthesized inside activated carbon (AC) using four first-row transition metals, namely Mn, Fe, Co, and Cu [64]. The ligand was adsorbed in AC pores, and then, the metal source was added to generate the corresponding MOF. Catalytic activity strongly depended on the metal and decreased in the order of Mn > Fe > Cu > Co. Therefore, only the Mn-based catalyst, denoted as Mn-BTC@AC, is discussed here. XRD revealed the presence of Mn_2_O_3_, which is known to catalyze the ORR via the undesirable two-electron pathway. SEM and TEM imaging showed that the catalyst comprised large aggregates of irregular sub-micrometer- to nanometer-scale (≈100 nm) particles. The BET surface area was obtained as 616 m^2^ g^−1^, and Mn content was estimated by ICP as 2.2 wt %. All catalysts containing Mn, Fe, Co, and Cu featured lower ORR activity (in term of current density and *E*_onset_/*E*_1/2_) than Pt/C but still outperformed pure AC. Mn-BTC@AC exhibited onset and half-wave potentials of 0.9 and 0.79 V, respectively, while lower respective values of 0.5 and 0.41 V were obtained for AC. According to RRDE measurements, the average *n* was calculated as 3.65, but no peroxide yield was provided. Catalyst durability was tested by imposing a constant potential of 0.6 V during 12 h, and 21% of the initial current density was lost at the end of the experiment. No comparison with Pt/C was performed.

An efficient ORR catalyst with atomically dispersed N-coordinated single Mn sites on partially graphitized carbon (Mn-N-C) has recently been reported by Wu et al. [65]. In the first step, Mn^2+^ and Zn^2+^ ions were combined to prepare Mn-doped ZIF-8 precursors. After carbonization and acid leaching, the obtained porous carbon was used as a host to adsorb additional Mn (MnCl_2_) and N sources (see Table 2 for details, cyanamide gave the best results), which was followed by another thermal treatment. The best-performing sample, denoted as 20Mn-NC-second, featured a homogeneous distribution of polyhedral carbon particles with a size of ≈50 nm. Both XRD patterns and scanning transmission electron microscopy (STEM) images verified the absence of crystalline Mn-containing phases or clusters in the partially graphitized carbon catalyst. The Fourier transform extended X-ray absorption fine structure (EXAFS) spectrum of 20Mn-NC-second revealed the presence of Mn–N and Mn–C bonds, and the first-shell coordination number of Mn–N was calculated as 3.2 ± 1.0 according to the results of Mn K-edge EXAFS spectrum fitting, suggesting that Mn-N_4_ was the dominant Mn–N structure. In addition, the corresponding N 1s spectrum contained a peak (399 eV) ascribed to metal-bonded N atoms, most probably Mn–N_4_. The percentage of Mn–N_4_ active sites was estimated at 11.2%. The bright spots observed in the medium-angle annular dark-field STEM images corresponded to single heavy atoms uniformly dispersed throughout the carbon structure, indicating a high density of Mn doping. In an acidic electrolyte, the best-performing 20Mn-NC-second catalyst presented an LSV curve shaped similarly to that obtained for the standard electrocatalyst, while the *E*_1/2_ of 20Mn-NC-second was only 60 mV lower than that of Pt/C (≈0.86 V). The peroxide yield was less than 2% within a large potential window of 0.05–0.75 V, indicating a four-electron reduction pathway. Catalyst durability was probed by chronoamperometric experiments at different constant potentials and CV scanning for thousands of cycles. For instance, when stability was tested by applying a constant potential of 0.7 V for 100 h during the ORR, the catalyst retained 88% of the initial current density and exhibited a 29-mV loss of *E*_1/2_.

**Table 2:** Recent research works (2018–2020) dealing with the preparation of Mn-based electrocatalysts for fuel cells with a focus on ORR activity. Examples in acidic and alkaline media are provided, and major physicochemical properties and electrochemical performances realized using rotating electrodes are compared.

				RDE/RRDE Experiments ***					
Precursors	Main Catalytically Active Sites	BET Surface Area (m^2^ g^−1^)	N/M (N = nitrogen; M = Metal) Contents (at%) *	*E*_1/2_ (V vs. RHE) **	Average n	Peroxide Yield	Cyclability	Methanol Tolerance	Reference
ZIF-8 + Mn(NO_3_)_2_	MnO_2_ + Mn_3_O_4_	1497	4.3/2.7	0.871 (1600 rpm and 10 mV s^−1^)	≈3.75	<2% (0.2–0.8 V)	Stability tested by CV measurements (50 mV s^−1^, 0.6–1.2 V, 8000 cycles). negative *E*_1/2_ shift of 2 mV	Excellent under alkaline conditions	[63]
H_3_BTC + Mn(NO_3_)_2_,	Mn_2_O_3_	616	n/a/2.2 (wt %)	0.79 (900 rpm and 5 mV s^−1^)	≈3.65	n/a	After 12 h, ≈79% of initial current retained at 0.6 V, 900 rpm	n/a	[64]
Mn-doped ZIF-8 + MnCl_2_ + nitrogen sources (dipicolylamine, cyanamide, phenanthroline, or melamine)	Mn-N_4_	658	1.3/0.1	0.5 M H_2_SO_4_: 0.8 (900 rpm and 1.67 mV s^−1^)	n/a	<2% (0.05–0.75 V)	After 100 h, 87.5% of initial current retained at 0.7 V, 200 rpm	n/a	[65]

* Sometimes, weight percentage was used, and hence, “wt %” data are provided. More than one metal or heteroatom can be provided depending on the active sites involved. ** Potentials are referenced to RHE except when stated otherwise. *** All the ORR performance values listed were measured in 0.1 M KOH except when stated otherwise.

### 3.3. Fe-Based Electrocatalysts

Liu et al. proposed a novel ZIF-8 “thermal melting” strategy to synthesize 2D porous Fe-N-doped graphene nanosheets (Fe-N/GNs) [66]. The incorporation of graphene oxide nanosheets during the synthesis of Fe-modified ZIF-8 decreased the crystal growth rate to favor the formation of ≈50-nm-diameter rhombic dodecahedral nanocrystals. The resulting material was thermally molten on graphene nanosheets and then pyrolytically decomposed to generate a thin active porous carbon layer. The obtained catalyst featured a high specific surface area of 1237.9 m^2^ g^−1^ that permitted the electrolyte to easily access single-atom Fe active sites during electrochemical reactions. High-angle annular dark-field STEM (HAADF-STEM) revealed the presence of isolated Fe atoms in Fe-N/GNs. This feature benefitted ORR activity, which was evaluated using RDE and RRDE techniques in alkaline (0.1 KOH) and acidic (0.1 M HClO_4_) media. In 0.1 M KOH, Fe-N/GNs showed better ORR activity (*E*_1/2_ = 0.903 V vs. RHE) than the standard Pt/C catalyst (*E*_1/2_ = 0.895 V), whereas the two electrocatalysts featured similar *E*_1/2_ values in 0.1 M HClO_4_. Average *n* values of 4.05 and 3.91 were calculated from K-L plots for the ORR in alkaline and acidic media, respectively, demonstrating the efficient reduction of O_2_ via the four-electron pathway. Consequently, the H_2_O_2_ yield in 0.1 M HClO_4_ remained less than 1% at all potentials. Chronoamperometric measurements in 0.1 M HClO_4_ showed that the Fe-N/GN electrocatalyst retained no less than 98% of its initial current after the application of a constant voltage for 11 h, whereas Pt/C showed a loss of 48%. These results were attributed to the well-graphitized structure that ensured good electrical conductivity for electron transfer, while the high specific surface area offered abundant space to uniformly distribute the atomically isolated Fe active sites.

Wang et al. [67] introduced an “MOF-protective-pyrolysis” strategy for the preparation of Fe-N-C catalysts, in which NH_2_-MIL-101-Fe (FeMOF) with a regular structure was selected as an Fe source, while the ZIF-8 shell acted as a protective layer and a source of carbon and nitrogen. TEM images acquired after pyrolysis revealed that the octahedral FeMOF was still well surrounded by ZIF-8 and that carbon nanotubes grew on the surface of the pyrolyzed material. According to XPS results, the obtained electrocatalyst (denoted as P-FeMOF@ZIF-8) contained 8.93 at% N and 0.95 at% Fe, while ^57^Fe Mössbauer spectroscopy indicated that Fe in P-FeMOF@ZIF-8 existed predominantly as Fe(II)-N_4_. The formation of Fe(II)-N_4_ active sites as well as the mesoporous structure and the large specific area of 785 m^2^ g^−1^ make P-FeMOF@ZIF-8 a promising ORR electrocatalyst. This catalyst exhibited high four-electron-pathway selectivity, good stability, and excellent methanol tolerance in acidic and alkaline electrolytes. According to the authors, the Fe(II)-N_4_ active sites accelerate the decomposition of H_2_O_2_ via the Fenton reaction, and the hydroxyl radicals produced in this process oxidize carbon to carbon-containing free radicals to accelerate the adsorption and reduction of O_2_. The nitrogen in P-FeMOF@ZIF-8 stabilizes the metal in a suitable valence state to act as direct active sites and promote the decomposition of H_2_O_2_ to yield superior ORR activity.

An Fe-N-C catalyst was prepared by the co-calcination of NH_2_-MIL-101@polydopamine (PDA) and melamine [68]. TEM imaging showed that this catalyst exhibited a crumpled structure and featured well-dispersed ≈60-nm-diameter Fe species wrapped in graphitic layers. Interestingly, a control sample synthesized without the addition of PDA exhibited obvious aggregation, which revealed the important role of PDA in preventing Fe species from aggregating. The existence of Fe-N*_x_* active sites was verified by XRD, and the corresponding patterns showed peaks attributable to Fe_3_N and Fe_24_N_10_ planes as well as revealed the absence of Fe_3_O_4_. The electrocatalyst featured a high N content (8.07 at%), a large specific surface area of 584.6 m^2^ g^−1^, and half-wave potentials of 0.844 and 0.657 V in alkaline and acidic electrolytes, respectively. The calculated *n* was close to four in both media, and an HO_2_^−^ yield of ≈6% was obtained in 0.1 M KOH (cf. 0.5% in an acidic medium), which was comparable to that obtained for the Pt/C catalyst under identical conditions and implied a high four-electron-pathway selectivity. The MOF-based electrocatalyst retained 93% of its initial current after 34,000 s of continuous reduction, featuring better long-term durability than Pt/C.

A MOF-derived doped-carbon catalyst with a high density of active sites/single Fe atoms, optimized porosity, and high surface area was prepared by Deng et al. [69] using a metallorganic gaseous doping strategy. Ferrocene (Fe source) was vaporized and trapped into a ZIF-8 host structure, and, together with the organic skeleton, it was pyrolyzed at 950 °C to yield a highly porous carbon-based catalyst with single Fe atoms coordinated by N on the carbon skeleton. The abundant bright dots and the absence of agglomerates observed by TEM indicated that Fe was effectively dispersed at the atomic scale. The material had a specific surface area of 1255 m^2^ g^−1^ and N/Fe contents of 5.51/1 at%. The density of ORR-active Fe atoms was calculated as 1.75812 × 10^13^ cm^−2^. In alkaline solution, the above catalyst showed interesting ORR performance with *E*_1/2_ = 0.864 V (cf. *E*_1/2_ = 0.814 V for Pt/C). High *n* values of 3.97 and 3.98 were obtained in alkaline and acidic media, respectively. In addition, the catalyst generated negligible amounts of H_2_O_2_ (<3% and 2%, respectively), thus strongly favoring the four-electron process in both electrolytes.

A dual-MOF strategy relying on the mixing of ZIF-8 (support precursor) and MIL-101(Fe) (Fe precursor) at different mass ratios followed by mixture pyrolysis was developed [70]. Aberration-corrected scanning transmission electron microscopy (AC-STEM) of the produced material showed the presence of numerous atomic-scale bright spots attributed to atomically dispersed Fe in the carbon matrix. Moreover, elemental mapping confirmed the homogeneous distribution of N and Fe, while Mössbauer spectroscopy confirmed the presence of FeN_4_ active sites. According to the authors, MIL-101(Fe) first disintegrates and collapses into fragments to provide efficient contact with the outer surface of the unreacted ZIF-8 during pyrolysis between 300 and 600 °C. Subsequently, ZIF-8 is carbonized to form a support, while Fe in the MIL-101(Fe) fragments is coordinated by N in ZIF-8 to form FeN*_x_* sites. RRDE tests demonstrated that ORR on this catalyst followed a near-four-electron pathway between 0.2 and 0.8 V and produced peroxide in <5% yield. The electrocatalyst retained 95.9% of its initial current after 40,000 s at a constant potential of 0.5 V. Interestingly, a poisoning experiment was performed utilizing SCN^−^, which was strongly adsorbed and limited the access of O_2_ to metallic sites, thus leading to a significant performance decay. This behavior confirmed that the high ORR activity of the above catalyst was due to FeN_4_ sites.

A new ORR electrocatalyst has recently been prepared by carbonizing electrospun poly(acrylonitrile) nanofibers with embedded nanocrystals of an iron-based MOF, MIL-88B-NH_2_ [71]. The non-pyrolyzed catalyst contained homogeneously embedded MIL-88B-NH_2_ nanocrystals and uniform nanofibers with rough surfaces and an average diameter of ≈220 nm, whereas the pyrolyzed composite contained continuous and uniform porous carbon nanofibers (diameter = 300 nm) with abundant and well-dispersed 30-nm Fe_3_C nanoparticles. The pores in carbon nanofibers originated from the acid etching of Fe nanoparticles and accounted for a large specific surface area of 557 m^2^ g^−1^. Remarkably, the above composite featured high electrocatalytic activity in an alkaline electrolyte, exhibiting *E*_onset_ = 1.012 V and *E*_1/2_ = 0.873 V, which surpassed the corresponding values of Pt/C by 13 and 46 mV, respectively. The electron transfer number was calculated as ≈3.80 at 0.55–0.80 V, demonstrating a preference for the four-electron pathway. Long-term stability testing showed a high retention (73%) of the initial current density for the MOF-based catalyst after 30,000 s, whereas Pt/C suffered from nanoparticle migration and therefore experienced a rapid activity loss (current retention = 62%). In addition, superb electrocatalytic performance was obtained in 0.5 M H_2_SO_4_. These results were ascribed to the 1D structure that provided a large exposed surface area, high electrical conductivity (high graphitization degree of the carbon shell), numerous Fe_3_C active sites for the ORR, and hierarchical macro-, meso-, and microporous structures that enhanced mass transportation.

A three-dimensional hierarchical open-porous carbon framework characterized by abundant Fe-N*_x_* active sites without the less active Fe-based nanoparticles was obtained by the pyrolysis of ZIF-8 using g-C_3_N_4_ as an etching agent [72]. The ZIF-8 precursor contained well-dispersed polyhedral particles with sizes of 50–100 nm, while a three-dimensional hierarchical open-porous structure with interconnected and wrinkled sheets was observed after pyrolysis. During annealing above 550 °C, the degradation of g-C_3_N_4_ led to the formation of highly corrosive species (e.g., C_2_N_2_^+^, C_3_N_2_^+^, or C_3_N_3_^+^) that reacted with Fe via nitrogen-doping reactions to form Fe-N*_x_* moieties. TEM imaging revealed abundant bright dots, demonstrating that Fe was dispersed at the atomic scale. In addition, the XPS-determined contents of N (6.6 at%) and Fe (4.05 at%) suggested the presence of abundant active sites such as Fe-N, pyridinic-N, and graphitic-N. The above catalyst exhibited outstanding ORR performance in alkaline and acidic electrolytes, e.g., the half-wave potential of 0.845 V obtained in 0.1 M KOH was ≈30 mV higher than that of commercial Pt/C, while the Tafel slope of 69 mV dec^−1^ was much lower than that of Pt/C (86 mV dec^−1^), indicating faster electron transfer in the novel catalyst. The electron transfer number was estimated at 3.95–3.99, and the yield of H_2_O_2_ was less than 2% within a 0.1–0.8 V potential window. The long-term stability of the MOF-based electrocatalyst was evaluated by chronoamperometric measurements for 40,000 s, and the observed current retention (95.6%) exceeded that of the benchmark Pt/C catalyst (85%).

Another Fe-based electrocatalyst (Fe/N-PCNs) prepared using g-C_3_N_4_ as a nitrogen source was reported by Zheng et al. [73], who first synthesized g-C_3_N_4_ by the thermal decomposition of urea at 550 °C and then prepared a Zn/Fe-MOF via a hydrothermal reaction between Zn(NO_3_)_2_·6H_2_O, FeSO_4_·7H_2_O, and 1,4,5,8-naphthalenetetracarboxylic anhydride as an organic ligand. Finally, the mixture of g-C_3_N_4_ and Zn/Fe-MOF was heated under Ar at 950 °C for 1 h (the best conditions found by the authors) to afford Fe/N-PCNs. The MOF precursor contained 2D nanosheets with a lateral size of <1 µm and an average thickness of <25 nm. After pyrolysis, the 2D structure was retained, but the external surface of the catalyst became much rougher, and the thickness decreased to 20 nm. HAADF-STEM imaging showed that the doped metal was well dispersed in the catalyst as single atoms and clusters of 2–4 atoms. According to the authors, the Zn nodes acted as separators to complicate the aggregation of Fe atoms. At the same time, the decomposition of g-C_3_N_4_ generated N_4_ moieties capable of complexing Fe atoms to form abundant Fe-N*_x_* active sites. XPS analysis revealed that nitrogen involved in the formation of these sites accounted for no less than 29.86%. The high density of Fe-N*_x_* active sites coupled with a high BET surface area of 864 m^2^ g^−1^ led to enhanced ORR activity. In an alkaline medium, Fe/N-PCNs presented *E*_1/2_ = 0.86 V, which slightly exceeded that of Pt/C (0.84 V). The electron transfer number was obtained as ≈3.95, but the H_2_O_2_ yield was not calculated, and the results for Pt/C were not presented. Stability tests showed that the Fe/N-PCN catalyst was more stable than Pt/C in alkaline and acidic media. In fact, after 20,000 s of long-term chronoamperometric measurements, the initial current density decreased by 3.1% in 0.1 M KOH and by 10% in 0.1 M HClO_4_, whereas losses of 19.3% and 20.4%, respectively, were observed under identical conditions for Pt/C.

Several Fe-N-C catalysts incorporated into ZIF-8 were prepared using solid-state reactions by varying parameters such as Fe content and atmosphere employed for MOF formation and calcination [74]. The best-performing catalyst, denoted as C-Fe2-Z8-Ar, was synthesized using an iron content of 2 at% by 3-h pyrolysis in Ar at 1000 °C. TEM imaging revealed an amorphous carbon structure with highly dispersed Fe atoms, which was conducive to the formation of Fe-N*_x_* sites and the realization of high ORR activity. The material also had a large specific surface area of 1235.4 m^2^ g^−1^ and high pyridinic (49.4%) and graphitic (50.6%) N contents desirable for increasing the positive charge density on carbon atoms, oxygen adsorption, and O=O bond weakening. RRDE measurements in O_2_-saturated 0.1 M KOH showed that C-Fe2-Z8-Ar exhibited excellent ORR performance with *E*_1/2_ = 0.831 V (comparable to that of commercial Pt/C (0.838 V)) and a high *E*_onset_ of 0.98 V. A high value of *n* = 3.98 was obtained in the range of 0.2–0.8 V, and the stable H_2_O_2_ yield was less than 1%, which indicated a direct four-electron reduction of O_2_. Finally, C-Fe2-Z8-Ar exhibited remarkable ORR stability, featuring a current density loss of only 3.9% after 10,000 s at a constant potential of 0.75 V.

Jiao et al. reported a new proof-of-concept catalyst synthesis via chemical vapor deposition (CVD) of FeCl_3_ at a relatively low temperature of 750 °C onto an N-doped carbon substrate to form abundant Fe-N_4_ sites [75]. FeCl_3_ was chosen as the Fe precursor owing to its low boiling point (316 °C), while the N-C substrate was prepared by mixing a ZIF-8 MOF and 1,10-phenanthroline via dry ball-milling followed by pyrolysis at 1050 °C under Ar. Before pyrolysis, ZIF-8 presented a uniform crystal size of ≈40 nm, while the collapse of the crystal structure after impregnation and thermal treatment led to the formation of an amorphous carbon matrix with a layered structure and amorphous Fe clusters. The catalyst, denoted as FeNC-CVD-750, was first probed by CV cycling in N_2_-saturated 0.5 M H_2_SO_4_ between 0.05 and 0.95 V. The prominent Fe^3+^/Fe^2+^ redox observed around 0.64 V evidenced the presence of abundant electrochemically active Fe-N_4_ sites, while the high electrochemical surface area of 1176 m^2^ g^−1^ agreed with the specific surface area of 970 m^2^ g^−1^. RDE experiments revealed a half-wave potential of 0.82 V in O_2_-saturated 0.5 M H_2_SO_4_ electrolyte with a fairly high *J*_L_ of ≈4 mA cm^−2^ (900 rpm) between 0.05 and 0.6 V. Unfortunately, no long-term stability or methanol tolerance data were presented. More interestingly, FeNC-CVD-750 was directly used to prepare a cathode for MEA tests in a proton-exchange membrane fuel cell (PEMFC) under H_2_-O_2_ conditions. According to the authors, the current density of 0.033 mA cm^−2^ obtained at a voltage of 0.9 V was 1.5-fold higher than the highest value reported to date for a PGM-free catalyst in an H_2_-O_2_ PEMFC. In addition, a current density of 0.044 mA cm^−2^ was obtained at 0.89 V, i.e., at a voltage only 0.01 V lower than the DOE 2020 target [76]. 

Huang et al. prepared a highly efficient ZIF-8-derived Fe-N-C catalyst for the ORR in both alkaline and acidic media [77]. The fairly long synthesis involved the decoration of a ZIF-8 MOF with FeCl_3_ through a wet impregnation method followed by treatment with a KOH solution to afford a core–shell Fe-(OH)_3_@ZIF-8 material with polyhedral morphology and a particle size of ≈50 nm, which was subsequently pyrolyzed under N_2_ at 1000 °C to form iron oxide. Except for a slight deformation, the catalyst roughly preserved the original shape of the precursor, in contrast to control samples prepared in the absence of FeCl_3_ or KOH. This result was attributed to the supporting effect of the in situ generated Fe_2_O_3_ template, which helped to avoid the rapid collapse of the ZIF-8 structure. Finally, a mesoporous Fe-N-C electrocatalyst with abundant mesopores and a specific surface area of 1021 m^2^ g^−1^ was obtained. The combination of a thin (≈5 nm) carbon shell with a hollow interior (≈48 nm) increased the accessibility of active sites to the electrolyte and significantly reduced mass transfer resistance. The ORR activity of Fe-(OH)_3_@ZIF-8 was probed by CV measurements, which revealed that the well-defined oxygen reduction peak at 0.73 V observed in O_2_-saturated 0.1 M HClO_4_ was not detected in N_2_-saturated solution. In acidic solution, the catalyst showed onset and half-wave potentials of 0.91 and 0.80 V, respectively, which were close to the values obtained for the benchmark Pt/C cathode (0.99 and 0.86 V, respectively); thus, it was ranked as one of the best Fe-N-C catalysts in acidic media. In 0.5 M KOH, this catalyst outperformed Pt/C, showing *E*_1/2_ = 0.88 V (30 mV higher than that of Pt/C). The electron transfer number (>3.9) and H_2_O_2_ yield (<5.0%) were very close to those of Pt/C. A sudden negative shift of *E*_1/2_ (by 50 mV) was observed upon the addition of KSCN, showing the ORR catalytic activity of Fe-N*_x_* sites. Finally, an H_2_-O_2_ PEMFC assembled with the pyrolyzed Fe-(OH)_3_@ZIF-8 cathode catalyst delivered a maximum power density of 411 mW cm^−2^ at 0.35 V and showed a current density decay of 12.5% after a 12 h operation at a constant voltage of 0.4 V in an H_2_-air configuration.

Shui et al. prepared several Fe-N-C single-atom catalysts with identical morphologies and very similar contents of atomic Fe-N*_x_* active sites, varying the Fe-N coordination number by changing the calcination temperature of a ZIF-8 MOF with adsorbed Fe^3+^ ions from 300 to 1000 °C [78]. ZIF-8 nanoparticles were synthesized by a liquid-phase method and pyrolyzed to yield N-doped carbon, which was impregnated with FeCl_3_ and annealed at 300, 500, or 1000 °C under Ar. The best catalytic performance was obtained for the sample calcined at 1000 °C (denoted as FeNC-1000), which comprised well-defined dodecahedral particles of ≈100-nm size and featured a specific surface area of 1188 m^2^ g^−1^. SEM elemental mapping showed that Fe and N were uniformly distributed on carbon, and HAADF-STEM revealed the presence of atomically dispersed Fe atoms (as follows from the observation of bright spots). The EXAFS spectrum presented a main peak around 1.48 Å, which was assigned to Fe-N coordination, while the lack of a Fe-Fe peak indicated the absence of Fe nanoparticles or clusters. According to EXAFS fitting results, the N coordination number of FeNC-1000 was estimated as four. Density functional theory (DFT) calculations suggest that ORR activity decreases in the order of Fe-N_4_ > Fe-N_3_ > Fe-N_2_ > Fe-N_1_ > Fe-N_5_. Thus, Fe-N_4_ has the most stable configuration and the highest ORR activity among all Fe-N*_x_* active sites, which is in line with the superior performance of the sample calcined at 1000 °C, which predominantly contained Fe-N_4_ sites. This catalyst presented a high *J*_L_ and featured *E*_onset_ = 0.894 V and *E*_1/2_ = 0.804 V in 0.5 M H_2_SO_4_, which were slightly lower than those of the benchmark Pt/C catalyst. Notably, 98.7% of the initial current was retained after 10,000 s at 0.5 V, which was indicative of high stability. Unfortunately, the stability of Pt/C was not tested under these conditions. Finally, the novel catalyst featured *n* ≈ 4 and a peroxide yield of <1% within a large potential range.

A recent study proposed the use of Cd instead of Zn as a low-temperature sacrificial metal for the synthesis of a new kind of MOF [79]. This strategy allows the generation of carbonaceous electrocatalysts from MOF precursors at a low pyrolysis temperature of 750 °C, which helps to preserve the single-atom Fe active sites and limit their agglomeration into metal nanoparticles. The precursor MOF was synthesized using 1,4-diazabicyclo[2.2.2]octane (DABCO) as the N-containing ligand, terephthalic acid as a second linker, and Cd and Fe nitrates as metal sources. The Fe-C-N catalyst was obtained after ball-milling in the presence of 1,10-phenanthroline and pyrolysis at 750 °C under NH_3_. SEM imaging revealed that the MOF precursor exhibited an orthorhombic morphology with a long-edge length of 5–7 μm, with a conversion to irregular shapes observed after pyrolysis. Inductively coupled plasma-mass spectrometry revealed that pyrolysis resulted in a nearly complete removal of Cd (residual content = 0.4 wt %). In addition, TEM imaging showed the presence of numerous single Fe atoms, which were identified by their high contrast and the small diameter of bright ≈1-Å spots. The Mössbauer spectrum of the Fe-C-N catalyst exhibited two sets of doublets assigned to single-atom Fe in FeN_4_ sites and Fe_2_N species. RDE tests were carried out in O_2_-saturated 0.1 M HClO_4_, and a half-wave potential of 0.70 V was obtained, while K-L analysis indicated a preference for the four-electron pathway. Although no long-term stability and SCN/methanol poisoning experiments or comparison with Pt/C were presented in this short communication, the reported results bring new opportunities for the design of low-temperature-pyrolyzed MOF–based electrocatalysts.

Sun et al. proposed an innovative strategy featuring the use of SiO_2_ chains to enhance the cathode mass transport by both tuning the mesoporous structures and increasing catalyst hydrophobicity [80]. More precisely, commercial ZIF-8 was ball-milled in the presence of an Fe source (1,10-phenanthroline and Fe(CH_3_COO)_2_) and hydrophobic fumed silica, which was followed by pyrolysis under Ar and NH_3_ to give SiO_2_-Fe/N/C, a catalyst with an interconnected structure containing agglomerated Fe particles and abundant voids of up to 100 nm in length. Raman spectroscopy showed that the use of SiO_2_ led to a larger number of defects in the carbon matrix (*I*_D_/*I*_G_ = 1.07 when SiO_2_ was used vs. 0.87 when SiO_2_ was not used), which are generally believed to be highly active for oxygen reduction. XRD revealed the presence of metallic Fe as well as small amounts of Fe_3_C and Fe_2_N, although only the single-atom-distributed FeN_4_-like species were the main ORR active sites. The pore size/volume distribution suggested that the MOF synthesized in the presence of SiO_2_ possessed a lower surface area (820 vs. 902 m^2^ g^−1^) but a larger total pore volume (0.78 vs. 0.66 cm^3^ g^−1^) than the material prepared without SiO_2_. Thus, the coexistence of micropores (which hosted active sites) and mesopores (which ensured sufficient mass transport) resulted in enhanced ORR performance. However, similar onset (0.88 V) and half-wave (0.80 V) potentials and current densities (5.3 mA cm^−2^) at 0.2 V were obtained for catalysts prepared with and without SiO_2_. In addition, catalyst performance was not compared to that of a standard Pt/C electrode, and the conclusions regarding the potential of this kind of catalyst were uncertain. The H_2_O_2_ yield (1–6% in the potential range of 0.2–0.8 V) and *n* (3.95–4.0) were close to the values obtained for the sample prepared without SiO_2_ (H_2_O_2_ < 2.5%, *n* > 3.97).

Song et al. prepared several Fe-ZIF-8 electrocatalysts and probed their ORR kinetics [81]. The authors intentionally introduced different amounts of H_2_O_2_ into the acidic electrolyte to study its effect on ORR catalyzed by Fe-ZIF-8 and Pt/C. First, the precursor amount was optimized to enhance ORR activity, with the best performance observed for the material prepared at a FeSO_4_:ZnCl_2_ mass ratio of 1:13.3 and denoted as 7.5Fe-ZIF-8. SEM imaging of the pyrolyzed material revealed the presence of carbon sheets with a porous morphology and without any visible metal particles, as confirmed by the absence of Fe metal, carbide, or oxide peaks in the corresponding XRD pattern. TEM imaging revealed that N and Fe were homogeneously distributed in 7.5Fe-ZIF-8, while XPS allowed the respective contents to be estimated at 5.35 and 0.38 at %. In addition, the catalyst featured a large surface area of 626 m^2^ g^−1^ suitable for high ORR activity. The fairly high half-wave potential of 0.76 V obtained for 7.5Fe-ZIF-8 in 0.1 M HClO_4_ was still lower than that of Pt/C (≈0.82 V). The high value of *n* (≈4) and the low yield of H_2_O_2_ (<1% between 0.2 and 0.8 V) make this electrocatalyst a perfect candidate to replace the standard Pt/C. After electrochemical characterization of the freshly prepared catalyst, the authors compared the influence of H_2_O_2_ content in the electrolyte on ORR performance, revealing that the ORR rate-limiting step is not the reduction of H_2_O_2_ but rather involves O_2_, i.e., it corresponds to O_2_ adsorption, activation, and reduction to H_2_O_2_.

A highly dispersed Fe-N*_x_* catalyst was prepared by the pyrolysis of Fe-Zn-ZIF nanocrystals synthesized in the presence of graphene support and polyvinylpyrrolidone (PVP) as a surfactant [82]. The use of graphene and PVP helped avoid the agglomeration of Fe particles during pyrolysis and precisely control the size and morphology of ZIF particles intercalated into graphene layers. The authors varied the amounts of the ZIF precursor, graphene, and PVP to optimize catalyst performance, showing that overly low loadings of graphene led to its curling, which was not observed at higher loadings that favored a more uniform ZIF distribution. PVP was found to regulate the ZIF particle size and morphology by coordinating and electrostatically interacting with Zn^2+^ ions. Pyrolysis afforded C-rGO-ZIF as a promising ORR catalyst with a specific surface area of 650 m^2^ g^−1^ and a high total pore volume of 2.03 cm^3^ g^−1^. ^57^Fe Mössbauer spectroscopy of C-rGO-ZIF showed that the main form of Fe in this catalyst was Fe^II^-N*_x_*, which agreed with the high content of Fe-N species revealed by XPS. C-rGO-ZIF presented an onset potential of 0.86 V and a half-wave potential of 0.77 V (only 30 mV lower than that of commercial Pt/C). Within a large potential window of 0.1–0.8 V, an average *n* of 3.8 and a peroxide yield of 2–3% were obtained, demonstrating a preference for the four-electron pathway. In contrast to Pt/C, this MOF-based material was insensitive to methanol addition into the electrolyte. Finally, long-term stability testing at a fixed potential of 0.8 V showed that C-rGO-ZIF retained 61% of its initial current density after 20 h, while Pt/C retained only 31%.

**Figure 6:**
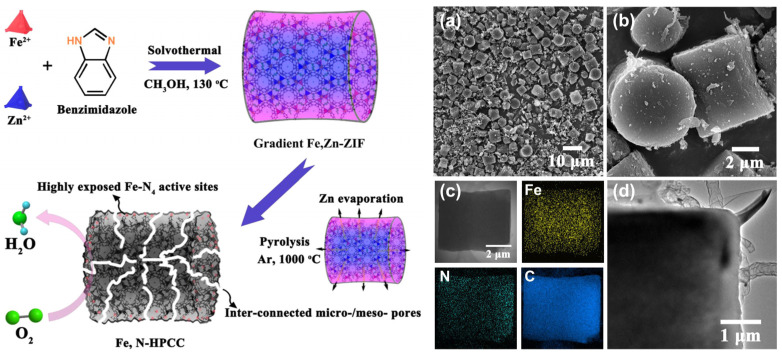
(**Left**) Schematic synthesis of a Fe- and N-co-doped hierarchically porous carbon cylinder catalyst denoted as Fe,N-HPCC. (**Right**) (**a**,**b**) SEM images, (**c**) STEM mappings of N, C, and Fe, and (**d**) TEM image of Fe,N-HPCC. Reprinted and modified with permission from ref. [83]. Copyright (2019) Elsevier.

Wang et al. reported a new class of Fe/Zn bimetallic ZIF materials with a cylindrical morphology and a well-defined transition metal concentration gradient featuring a Zn-rich core and an Fe-rich shell [83]. The optimal Zn:Fe molar ratio was identified as 20. Carbonization under Ar afforded an Fe- and N-co-doped hierarchically porous carbon cylinder catalyst denoted as Fe,N-HPCC. STEM mapping showed that the non-calcined MOF featured a pore diameter of ≈6.5 μm and an unusual gradient distribution of metal sites, with the Fe content gradually increasing upon going from the Zn-rich core to the outer shell (Figure 6). The large amount of Zn compared to that of Fe facilitated the nucleation and growth of the Zn-ZIF core, while an increase in Fe content and an enrichment of the MOF surface in Fe were observed with increasing reaction time. Pyrolysis at 1000 °C resulted in a uniform distribution of Fe and N, which favored the formation of Fe-N*_x_* sites. The large surface area of 817 m^2^ g^−1^ featured the contributions of micropores (0–2 nm; 373 m^2^ g^−1^) and mesopores (2–10 nm; 142 m^2^ g^−1^) that favored high ORR performance and were produced by Zn evaporation during calcination. The onset and half-wave potentials of Fe,N-HPCC in 0.1 M KOH equaled 0.972 and 0.898 V, respectively, with the latter value exceeding that of commercial Pt/C (0.847 V) by 50 mV. The peroxide yield was less than 10% in the potential window of 0.2–0.8 V, while *n* ranged from 3.8 to 3.9 and indicated a high preference for the four-electron pathway. Finally, Fe,N-HPCC retained 94.9% of its initial current density after 10,000 s at a constant potential, whereas the value of Pt/C equaled 79%. In an acidic medium, the former catalyst also showed a higher performance and longer lifetime than Pt/C, featuring excellent methanol tolerance in both media.

Chen et al. used Zn-mediated template synthesis to prepare a hierarchically porous carbon with densely exposed Fe-N*_x_* moieties (SA-Fe-NHPC) [84]. More specifically, SA-Fe-NHPC was fabricated by the pyrolysis of the nitrogen-rich 2,6-diaminopyridine/Zn^2+^-Fe^3+^/SiO_2_ complex followed by etching. 2,6-Diaminopyridine was selected as a ligand to promote the formation of Fe-N*_x_* sites because of its high N/C atomic ratio of 3/5, while SiO_2_ nanospheres were used as a hard template to generate a mesoporous carbon nanostructure after leaching with HF. TEM imaging revealed that the catalyst contained abundant pores with an average diameter of 12 nm, which agreed with the size of silica beads. In addition, HAADF-STEM imaging showed that Fe atoms were atomically dispersed on the carbon support and demonstrated the absence of metallic nanoparticles. The specific surface area and total pore volume of SA-Fe-NHPC were measured as 1327 and 2.7 cm^3^ g^−1^, respectively. The combination of a high surface area and pore volume as well as a hierarchical porous carbon structure and well-dispersed Fe-N*_x_* active sites contributed to enhanced electrochemical performance. SA-Fe-NHPC demonstrated high ORR activity in 0.1 M KOH, featuring an onset potential of ≈1.01 V and a half-wave potential of 0.93 V that was much higher than that of Pt/C (0.85 V). RRDE experiments showed that the H_2_O_2_ yield did not exceed 5% in the potential window of 0.2–0.9 V and was slightly less than that of Pt/C, while the electron transfer number of SA-Fe-NHPC (3.88–3.98) confirmed its superior selectivity for the four-electron reduction of O_2_. Long-term stability testing showed that the *E*_1/2_ of SA-Fe-NHPC after 10,000 CV cycles between 0.6 and 1.0 V at a scan rate of 50 mV s^−1^ decreased only by 1 mV. Finally, the authors demonstrated that SA-Fe-NHPC could be used as a cathode in Zn-air batteries and delivered a specific capacity of 795.3 mAh g^−1^ at 10 mA cm^−2^, which corresponded to ≈96.9% utilization of the theoretical capacity (820 mAh g^−1^).

**Figure 7:**
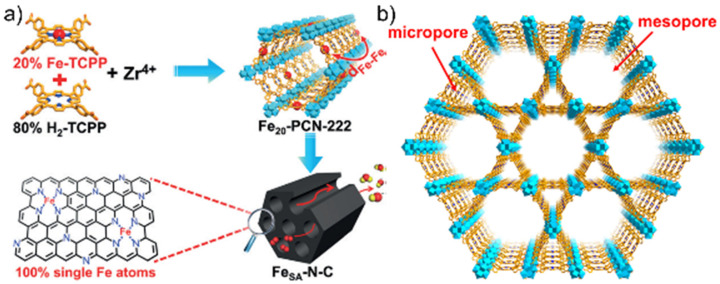
(**a**) Schematic synthesis of FeSA-N-C via the thermal decomposition of an Fe-containing metal–organic framework (MOF), PCN-222. (**b**) View of the 3D network of PCN-222 featuring mesochannels running through the *c*-axis. Reprinted and modified with permission from ref. [85]. Copyright (2018) John Wiley and Sons.

Jiao et al. fabricated an ORR catalyst through the thermal decomposition of a porphyrinic MOF (PCN-222) featuring 1D mesochannels with a diameter of 3.2 nm and well-defined micropores (≈1.2 nm) [85]. Figure 7 shows the structure of PCN-222 as well as the synthesis of this MOF and the related carbonaceous catalyst. For MOF synthesis, Fe-TCPP and H_2_-TCPP were used as ligands (optimized Fe-TCPP:H_2_-TCPP ratio = 0.2), while ZrOCl_2_ was used as a metal precursor. Pyrolysis afforded single-atom Fe sites implanted into porous N-doped carbon (Fe_SA_-N-C). The parent MOF had a fairly high BET surface area of 2062 m^2^ g^−1^ and comprised uniform rod-shaped particles with a diameter of ≈200 nm. Upon pyrolysis at 800 °C, the rod-like structure was preserved, and the resulting microporous material presented a BET surface area of 532 m^2^ g^−1^. XRD and Raman (*I*_D_/*I*_G_ = 0.95) analyses confirmed the occurrence of graphitization, and XPS revealed the presence of Fe-N*_x_* catalytic sites. Aberration-corrected HAADF-STEM imaging unambiguously identified single Fe atoms as bright spots dispersed in the carbon matrix, but no isolated Fe particles were found. The Fourier-transformed Fe K-edge EXAFS spectrum of Fe_SA_-N-C presented a single main peak at ≈1.44 Å, which was attributed to Fe-N scattering, and no Fe–Fe bond peak at ≈2.13 Å was detected, which confirmed the presence of single-atom Fe sites in the catalyst. In addition, EXAFS fitting showed that each Fe atom was coordinated by ≈4 N atoms. The electrochemical performance surpassed that of standard Pt/C even in an acidic electrolyte. Specifically, in 0.1 HClO_4_, Fe_SA_-N-C showed an onset potential of 0.93 V and a half-wave potential of 0.78 V, which were close to the values of Pt/C (0.91 and 0.78 V, respectively). RRDE test results (H_2_O_2_ yield < 1.0%, *n* = 4) demonstrated the high potential of this new catalyst. Moreover, Fe_SA_-N-C exhibited excellent durability in an alkaline medium, featuring an only 6-mV decay after 5000 CV cycles and a 4% drop of current after a 10-h chronoamperometric test, thus outperforming Pt/C.

In 2020, the same group reported a similar material, in which SiO_2_ was incorporated into the mesopores of PCN-222 [86]. The above MOF was synthesized as described in ref. [85] and impregnated with tetraethylorthosilicate (TEOS), which was converted to SiO_2_ by treatment with HCl vapor. The successful adsorption and conversion of TEOS in the mesopores were confirmed by the reduction of surface area (1190 m^2^ g^−1^) and mesopore size (2.9 nm) relative to those of the parent MOF (2040 m^2^ g^−1^ and 3.2 nm, respectively). Carbonization in a flow of N_2_ at 800 °C followed by treatment with HF to remove SiO_2_ yielded a single-atom Fe–implanted N-doped porous carbon (FeSA–N–C) with a high Fe loading (3.46 wt %) and a very high BET surface area (1615 m^2^ g^−1^, the highest value reported for a MOF-based electrocatalyst in this review). Consequently, the above catalyst featured exceptional ORR activity in acidic and alkaline media. For instance, in 0.1 M KOH, the catalyst presented a half-wave potential (0.9 V) exceeding that of Pt/C, and good performance (i.e., *E*_1/2_ = 0.8 V, *n* ≈ 4, peroxide yield < 1%) was observed even in an acidic electrolyte. Catalyst durability in both electrolytes was probed by performing 20,000 CV cycles. No potential shift was reported by the authors, but the quasi-overlap of LSV curves recorded before and after the 20,000 cycles indicated high stability in acidic and alkaline electrolytes. In addition, the above catalyst presented a high methanol tolerance. According to the authors, SiO_2_ prevented Fe aggregation, while its removal after thermal treatment strongly increased the porosity and surface area of the resulting N-doped porous carbon, facilitating active site exposure and mass transfer.

An atomically dispersed Fe-N-C catalyst with a high Fe content was synthesized by an in situ ionothermal method involving the dispersion of a large amount of Fe(II) phthalocyanine (FePc) and SiO_2_ in an imidazolium-based ionic liquid–impregnated ZIF-8 [87]. Pyrolysis and subsequent NH_3_ activation afforded Fe-IL@MOF-NH_3_, which featured a polyhedral morphology and comprised interconnected micro- and mesopores. TEM imaging and the corresponding elemental mappings showed that Fe, N, and C were uniformly distributed over the entire framework, which featured a BET surface area of 1381 m^2^ g^−1^. According to ICP and ^57^Fe Mössbauer spectroscopy measurements, the prepared catalyst contained ≈2.25 wt % Fe and abundant Fe-N*_x_* active sites. LSV testing in an acidic medium revealed a half-wave potential value of ≈0.791 V, which was slightly lower than that of 20 wt % Pt/C (0.820 V), and the high value of *n* (≈3.8) was indicative of a low peroxide yield (not given). Finally, Fe-IL@MOF-NH_3_ showed no noticeable change in the half-wave potential after 1000 voltammetric cycles, thus featuring superior durability.

Yang et al. prepared a hierarchically porous carbon with atomically dispersed Fe-N*_x_* active species using a nitrogen-rich bridging ligand (tetrapyridophenazine) as a nitrogen and carbon source [88]. The self-assembly of this ligand and Fe^2+^ ions upon solvothermal treatment yielded a 1D coordination polymer (Fe-tpphz) with an MOF-like structure and a flower-like morphology assembled from numerous variable-size nanosheets. After thermal treatment, slight particle shrinkage was observed, and some sheets fragmented into small pieces with abundant nanopores ideal for providing facile access to active sites for electrocatalysis. HAADF-STEM imaging revealed the uniform distribution of C, N, and Fe in the carbon matrix. In addition, numerous small isolated bright spots with diameters of 0.1–0.2 nm were observed and ascribed to single Fe atoms, favoring the formation of Fe-N*_x_* active centers. EXAFS data supported the formation of such active sites, with the single main peak (≈1.5 Å) corresponding to Fe–N coordination. Finally, the BET surface area of 357 m^2^ g^−1^ and the high pore volume of 1.07 cm^3^ g^−1^ provided more exposed active sites and faster transport. The MOF-based catalyst displayed a more positive onset potential (0.996 V) than Pt/C (0.983 V) in alkaline solution, featuring a half-wave potential (≈0.863 V) ≈21 mV higher than that of Pt/C. When catalyst durability was evaluated by CV in alkaline solution (8000 cycles between 0 and 1.0 V at a scan rate of 100 mV s^−1^), *E*_1/2_ shifted to more negative values by ≈19 mV, whereas a decrease of 59 mV was observed for Pt/C. Notably, better stability was also obtained in an acidic medium. Finally, in both alkaline and acidic media, average *n* values above 3.9 and peroxide yields lower than 4% were obtained.

A microporous MOF-confined method was developed to synthesize an Fe-N-C single-atom dispersed material [46]. More precisely, Fe-ZIF-8 was synthesized in the presence of a small quantity of Fe(acac)_3_ and pyrolyzed in an Ar/H_2_ mixture at temperatures ranging from 850 to 1050 °C, with the best catalyst (Fe-N-C-950) obtained at 950 °C. ICP analysis revealed that Fe-ZIF-8 had an Fe content of only 0.137 wt %; moreover, the fact that Fe incorporation did not affect the rhombododecahedral MOF morphology confirmed the successful incorporation of Fe(acac)_3_ into MOF pores. The XRD pattern of Fe-N-C-950 revealed the absence of crystalline Fe compounds, suggesting that Fe was mostly present as amorphous Fe-N*_x_* species. This hypothesis (especially the formation of Fe-N_4_ active sites) was confirmed by ^57^Fe Mössbauer spectroscopy. In addition, the Fourier transformed K-edge EXAFS spectrum of Fe-N-C-950 showed a distinct Fe-N peak at 1.44 Å, certifying the presence of Fe-N*_x_* coordination. The catalyst featured a high BET surface area of 1498 m^2^ g^−1^, which is one of the highest reported in the works discussed herein. The numerous bright dots observed in TEM images corresponded to isolated Fe and Zn atoms. ORR activity was investigated in 0.1 M HClO_4_, and the obtained onset and half-wave potentials (0.92 and 0.78 V, respectively) were less than those of Pt/C. RRDE experiments revealed an electron transfer number of >3.98 and a peroxide yield of <1% in the potential window of 0.1–0.8 V, which signified good performance in an acidic electrolyte. Finally, both Pt/C and Fe-N-C-950 presented good stability under a constant potential applied for several hours.

Horike et al. reported the preparation of an ɛ-Fe_2_N-containing catalyst from an Fe-based MOF [89]. An air-stable carbon Fe_3_C composite was prepared by the pyrolysis of an Fe-triazolate framework, [Fe(C_2_N_3_H_2_)_2_]*_n_*, in N_2_. The XRD pattern of the powder obtained at 800 °C featured several Fe_3_C peaks between 37 and 49°, while the corresponding high-resolution TEM (HR-TEM) images showed the presence of nanoparticles with crystalline lattices corresponding to the (001) and (100) planes of Fe_3_C. The resulting material was calcined in an atmosphere of NH_3_ to convert Fe_3_C to active ɛ-Fe_2_N sites, the presence of which was verified by the observation of three notable peaks (at 38, 41, and 43°) in the related XRD pattern. TEM imaging of the obtained material revealed ≈100-nm-diameter nanoparticles dispersed in carbon and covered with a 3–7-nm-thick carbon layer. In 0.1 M HClO_4_, the ɛ-Fe_2_N-containing catalyst showed an onset potential of 0.86 V and a half-wave potential of 0.66 V that was 100 mV less than that of the benchmark Pt/C catalyst. Interestingly, *n* = 3.9 obtained at 0.5 V decreased to 2.8 for the sample containing the Fe_3_C precursor, which indicated the poor electrocatalytic selectivity of this species. Durability tests in the range of 0.5–0.8 V revealed that the new catalyst showed excellent stability (76% current retention after 5000 cycles), whereas Pt/C showed poorer performance (51% current retention).

**Table 3:** Recent research works (2018–2020) dealing with the preparation of Fe-based electrocatalysts for fuel cells with a focus on ORR activity. Examples in acidic and alkaline media are provided, and major physicochemical properties and electrochemical performances realized using rotating electrodes are compared.

				RDE/RRDE Experiments ***					
Precursors	Main catalytically Active Sites	BET Surface Area (m^2^ g^−1^)	N/M (N = Nitrogen; M = Metal) Contents (at%) *	*E*_1/2_ (V vs. RHE) **	Average n	Peroxide Yield	Cyclability	Methanol Tolerance	Reference
ZIF-8 + graphene nanosheets	Fe-N_x_	1237.9	n/a/2.16	0.903 0.1 M HClO_4_: 0.837	3.98 0.1 M HClO_4_: 3.995	<2% (0.2–0.8 V) 0.1 M HClO_4_: <1% (0.2–0.8 V)	After 11 h, 94% of initial current 0.1 M HClO_4_: after 11 h, 98% of initial current	Excellent under acidic conditions	[66]
NH_2_-MIL-101@ZIF-8	Fe-N_4_	785	8.93/0.95	1.01 (onset potential) 0.5 M H_2_SO_4_: 0.85 (onset potential)	≈3.85 0.5 M H_2_SO_4_: ≈3.8	5% (0.25–0.5 V) 0.5 M H_2_SO_4_: 11% average (0.25–0.5 V)	After 2.8 h, ≈95% of initial current at 0.7 V 0.5 M H_2_SO_4_: after 2.8 h, ≈40% of initial current at 0.7 V	Excellent under alkaline and acidic conditions	[67]
NH_2_-MIL-101@polydopamine + melamine	Fe-N_x_	584.6	8.07/1.30	0.844 0.5 M H_2_SO_4_: 0.657	≈3.86 0.5 M H_2_SO_4_: ≈3.99–4.0	<6% (0.15–0.75 V) 0.5 M H_2_SO_4_: <0.5% (0.15–0.75 V)	After 9.4 h, ≈93% of initial current at 0.51 V	Excellent under alkaline conditions	[68]
ZIF-8 + ferrocene	Fe-N_4_	1255	5.51/1	0.864 0.1 M HClO_4_: 0.78	3.98 0.1 M HClO_4_: 3.97	<3% (0.2–0.8 V) 0.1 M HClO_4_: <2% (0.2–0.8 V)	After 5.6 h, 96% of initial current at 0.65 V, 900 rpm 0.1 M HClO_4_: after 5.6 h, 90% of initial current at 0.62 V, 900 rpm	n/a	[69]
ZIF-8 + MIL-101(Fe)	Fe-N_4_	681	9.64/0.8	0.1 M HClO_4_: 0.78	0.1 M HClO_4_: 3.94	0.1 M HClO_4_: ≈4% (0.2–0.8 V)	0.1 M HClO_4_: after 11.1 h, 95.9% of initial current at 0.5 V	Excellent under acidic conditions	[70]
MIL-88B-NH_2_ + polyacrylonitrile	Fe_3_C	557	4.52/6.13	0.873 0.5 M H_2_SO_4_: 0.664	0.1 M KOH: ≈3.80 (0.55–0.8 V)	n/a	After 8.3 h, 73% of initial current at 0.2 V vs. Ag/AgCl	Excellent under alkaline conditions	[71]
ZIF-8 + ferric acetylacetonate + C_3_N_4_	Fe-N_x_	≈540	6.6 (wt %)/4.05 (wt %)	0.845 0.5 M H_2_SO_4_: 0.745	3.95–3.99 0.5 M H_2_SO_4_: 3.94–3.97	<2% (0.1–0.8 V) 0.5 M H_2_SO_4_: <3% (0.2–0.8 V)	After 11.1 h, 95.6% of initial current 0.5 M H_2_SO_4_: after 11.1 h, 82% of initial current	n/a	[72]
NTCDA + Zn(NO_3_)_2_ + FeSO_4_ + C_3_N_4_	Fe-N_x_	864	10.28 (wt %)/3.89 (wt %)	0.86 0.1 M HClO_4_: 0.79	≈3.95 (0.5–0.7 V) 0.1 M HClO_4_: ≈3.80 (0.5–0.7 V)	n/a	After 5.6 h, 96.9% of initial current at 0.62 V, 900 rpm 0.1 M HClO_4_: after 5.6 h, 90.8% of initial current at 0.62 V, 900 rpm	n/a	[73]
Fe-doped ZIF-8	Fe-N_x_	1235.4	2.62/n/a	0.831(5 mV s^−1^)	3.98	<1% (0.2–0.8 V)	After 2.8 h, 96.1% of initial current at 0.75 V	n/a	[74]
ZIF-8 + 1,10-phenanthroline	Fe-N_4_	970	n/a/2.6 (wt %)	0.5 M H_2_SO_4_: 0.82 (900 rpm and 20 mV s^−1^)	n/a	n/a	n/a	n/a	[75]
ZIF-8 + FeCl_3_	Fe-N_x_	1021	5.78/0.68	0.88 V 0.1 M HClO_4_: 0.80 V	>3.9	<5% (0.2–0.8 V)	Stability experiments realized for H_2_-air PEMFC	Excellent under acidic conditions	[77]
ZIF-8 + FeCl_3_	Fe-N_x_	1188	5.93/3.91	0.903 0.5 M H_2_SO_4_: 0.804	0.5 M H_2_SO_4_: ≈3.9–4.0	0.5 M H_2_SO_4_: <1.0% (0.1–0.7 V)	0.5 M H_2_SO_4_: after 2.8 h, 98.7% of initial current at 0.5 V	n/a	[78]
PTA + DABCO + Cd(NO_3_)_2_ + Fe(NO_3_)_2_ + 1,10-phenanthroline	Fe-N_x_	431	n/a	0.1 M HClO_4_: 0.7 (900 rpm)	0.1 M HClO_4_: close to 4	n/a	n/a	n/a	[79]
ZIF-8 + SiO_2_ chains + FeAc + 1,10-phenanthroline	Fe-N_x_	820	2.08/0.32	0.1 M HClO_4_: 0.8	0.1 M HClO_4_: 3.95–4.0	0.1 M HClO_4_: ≈1–6% (0.2–0.8 V)	n/a	n/a	[80]
ZIF-8 + FeSO_4_	Fe-N_x_	626	5.35/0.38	0.1 M HClO_4_: 0.76 (5 mV s^−1^)	0.1 M HClO_4_: 3.95–4.0	0.1 M HClO_4_: <1% (0.2–0.8 V)	n/a	n/a	[81]
Fe(NO_3_)_3_ + Zn(NO_3_)_2_ + 2-mIm + GO + PVP	Fe-N_x_	650	3.47/0.40	0.1 M HClO_4_: 0.77	0.1 M HClO_4_: ≈3.8	0.1 M HClO_4_: ≈2–3% (0.1–0.8 V)	0.1 M HClO_4_: after 20 h, ≈61% of initial current at 0.8 V	Excellent under acidic conditions	[82]
FeSO_4_ + Zn(NO_3_)_2_ + BIM	Fe-N_x_	817	4.21/0.41	0.8980.1 M HClO_4_: 0.76	≈3.8–3.9 0.1 M HClO_4_: ≈3.95	<10.0% (0.2–0.8 V) 0.1 M HClO_4_: ≈2.1–3.6 (0.2-0.8 V)	After 2.8 h, ≈94.9% of initial current 0.1 M HClO_4_: after 2.8 h, ≈81.1% of initial current	Excellent under alkaline and acidic conditions	[83]
DAP + Zn(NO_3_)_2_ + Fe(NO_3_)_3_ + SiO_2_	Fe-N_x_	1327	5.6 (wt %)/1.25 (wt %)	0.93 (5 mV s^−1^)	≈3.88–3.98	<5.0% (0.4–0.9 V)	Stability experiments realized by CV (50 mV s^−1^, 0.6–1.0 V, 10000 cycles): negative *E*_1/2_ shift of 1 mV	Excellent under alkaline conditions	[84]
Fe-TCPP + H_2_-TCPP + ZrOCl_2_	Fe-N_x_	532	4.67 (wt %)/1.76 (wt %)	0.89 (5 mV s^−1^) 0.1 M HClO_4_: 0.78 (5 mV s^−1^)	>3.9	<5% (0.2–0.8 V)	After 10 h, 96% of initial current	Excellent under alkaline conditions	[85]
Fe-TCPP + H_2_-TCPP + ZrOCl_2_ + TEOS	Fe-N_x_	1615	4.87 (wt %)/3.46 (wt %)	0.9 0.1 M HClO_4_: 0.8	≈3.87–3.99 0.1 M HClO_4_: >3.98	<7% (0.2–0.8 V) 0.1 M HClO_4_: <1% (0.2–0.85 V)	Stability experiments realized by CV: quasi no variance in *E*_1/2_	Excellent under alkaline and acidic conditions	[86]
ZIF-8 + [bmim][Tf_2_N] + SiO_2_ + FePc	Fe-N_x_	1381	3.21/2.25	0.5 M H_2_SO_4_: 0.791 (5 mV)	0.5 M H_2_SO_4_: ≈3.8	n/a	Stability experiments realized by CV (50 mV s^−1^, 0.0–1.2 V, 1000 cycles): 0.5 M H_2_SO_4_: no variance in *E*_1/2_	Excellent under acidic conditions	[87]
Tpphz + FeSO_4_	Fe-N_x_	357	9.81/0.52	0.863 0.1 M HClO_4_: 0.633	≈3.93–3.98 0.1 M HClO_4_: >3.95	<3.6% (0.15–0.95 V) 0.1 M HClO_4_: <2.5% (0.1–0.75 V)	Stability experiments realized by CV (100 mV s^−1^, 0.0–1.0 V, 8000 cycles): negative *E*_1/2_ shift of 19 mV 0.1 M HClO_4_: negative *E*_1/2_ shift of 27 mV	n/a	[88]
ZIF-8 + Fe(acac)_3_	Fe-N_4_	1498	3.28/0.32 (wt %)	0.1 M HClO_4_: 0.78 (mV s^−1^)	0.1 M HClO_4_: >3.98	0.1 M HClO_4_: <1.0% (0.1–0.8 V)	0.1 M HClO_4_: after 4.2 h, ≈91% of initial current	n/a	[46]
FeCl_2_ + 1H-1,2,3-triazole	ɛ-Fe_2_N	554	4.5/0.7	0.1 M HClO_4_: 0.66	0.1 M HClO_4_: 3.9 (at 0.5 V)	n/a	Stability experiments realized by CV (0.5–0.8 V, 5000 cycles): 0.1 M HClO_4_: 76% of initial current	n/a	[89]

* Sometimes, weight percentage was used, and hence, “wt %” data are provided. More than one metal or heteroatom can be provided depending on the active sites involved. ** Potentials are referenced to RHE except when stated otherwise. The ORR performance values were evaluated at 1600 rpm and 10 mV s^−1^ except when stated otherwise. *** All the ORR performance values listed were measured in 0.1 M KOH except when stated otherwise.

### 3.4. Co-Based Electrocatalysts

**Figure 8:**
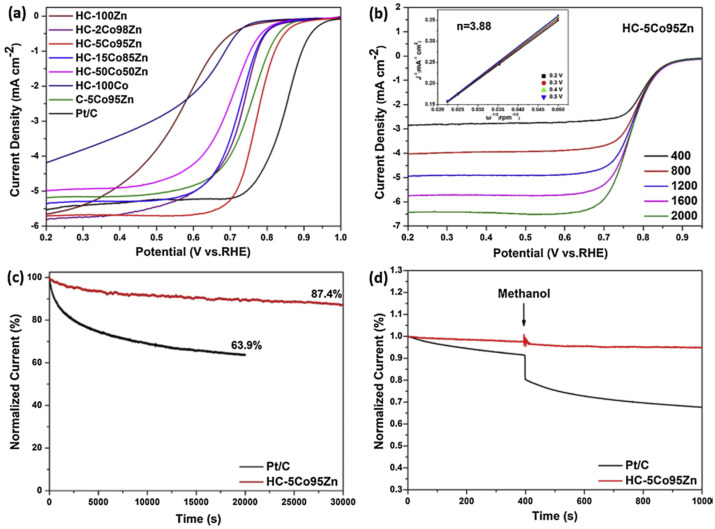
(**a**) Linear sweep voltammetry (LSV) curves recorded at 1600 rpm for several catalysts prepared with various concentration of Co and Zn, and Pt/C for ORR; (**b**) LSV curves for the best sample (denoted HC-5Co95Zn) collected at various rotation speeds, the inset is the corresponding K-L plots; (**c**) Chronoamperometric responses and (**d**) methanol tolerance tests of Pt/C and HC-5Co95Zn catalysts. All tests were performed in O_2_-saturated 0.1M HClO_4_. Reprinted with permission from ref. [90]. Copyright (2019) Elsevier.

A bimetallic organic framework-derived hierarchically porous electrocatalyst was synthesized via a three-step process [90]. 2-Methylimidazole was reacted with P123 (surfactant) to form micelles with ligands self-assembled by hydrogen bonds on their surface; then, Co and Zn nitrates were used to generate a bimetallic ZIF. The introduction of the low-boiling-point (906 °C) Zn into the ZIF was useful for creating additional micropores during carbonization. Consequently, a high specific surface area of 1539 m^2^ g^−1^ was obtained. The above electrocatalyst showed high ORR activity (see Figure 8, which reports the results of some electrochemical tests performed with RDE in 0.1M HClO_4_), featuring a half-wave potential of 0.78 V and a limiting current density of 5.7 mA cm^−2^ (at 1600 rpm) that exceeded the value obtained for Pt/C (≈5.3 mA cm^−2^). In addition, better long-term stability (12.6% decrease in initial current after 30,000 s at a constant potential of 0.45 V) was obtained in an acidic medium, whereas an initial current decrease of 36.1% was obtained for Pt/C after a 20,000-s stability test (see Figure 8c). The new catalyst was also tested as the cathode of a real fuel cell containing a Pt-impregnated carbon cloth and supplied with pure O_2_ and H_2_. The maximum power density calculated for the MOF-based catalyst (412 mW cm^−2^) was slightly lower than that of Pt/C (590 mW cm^−2^).

The controllable synthesis of CoN_3_ nanoparticles embedded in graphitized carbon was successfully achieved through the in situ pyrolysis of Co/Zn-ZIF-67 [91], which afforded Co nanoparticles and NH_3_ (via ZIF decomposition). The abundant micropores formed by the evaporation of Zn and the large surface area of the obtained material facilitated the reaction between NH_3_ and CO to afford 40–70-nm CoN_3_ particles well dispersed in the carbon matrix. The resulting material presented *E*_1/2_ = 0.72 V and a current density of 5.4 mA cm^−2^, which were similar to those obtained for Pt/C in 0.5 M H_2_SO_4_. Moreover, *n* = 3.90 was calculated for the entire investigated potential range, while the H_2_O_2_ yield was close to 6%, which indicated a strong preference for the four-electron reduction. The developed material also exhibited excellent performance and stability as a cathode catalyst in an H_2_/O_2_ fuel cell. DFT calculations performed to explain this high performance revealed that the (220) facets of CoN_3_ nanoparticles have a low energy barrier and facilitate the adsorption of O_2_ and the ORR process in acidic electrolytes.

Three-micron ZnO@ZIF-8 cubes impregnated with metal –ammonia complexes (MACs, metal = Ag, Co, Ni, or Cu) were calcined at 800 °C in a flow of Ar to yield a 3D cubic porous structure [92]. The Co-containing MOF presented the best performance. HR-TEM imaging of this catalyst showed that the entire porous carbon skeleton was covered by graphitized carbon, with Co and Zn located in the shell and the core of the cube, respectively. The material exhibited a specific surface area of 678.8 m^2^ g^−1^, and the presence of Co-N*_x_* active sites was confirmed by the presence of two main peaks (784.3 and 800.3 eV) in the Co 2p core-level spectrum. The Co-based catalyst showed high catalytic activity, featuring a half-wave potential of 0.8 V, an onset potential of 0.91 V, and a current density of 5.88 mA cm^−2^ at 1600 rpm in an alkaline electrolyte. After a continuous 10 h operation at 0.6 V, the current was maintained at 97.4% of the initial value for the pyrolyzed MOF, when Pt/C lost almost 10% of its initial current. The above electrocatalyst was also used as the Pt-free cathode of an in-house-built Zn-O_2_ battery.

ZnCo bimetallic MOFs with different Zn/Co molar ratios and dispersed Co sites were prepared by an in situ transformation method [93], with the best results obtained at Zn/Co = 5:1. SEM imaging showed the presence of rhombododecahedral ZIF particles with sizes of ≈100 nm. After dopamine polycondensation and carbonization, a spherical carbon nanostructure with well-dispersed Co was obtained. The prepared catalyst exhibited an excellent intrinsic ORR activity in 0.1 M KOH, featuring onset (0.94 V) and half-wave (0.838 V) potentials similar to those obtained for the control Pt/C catalyst. The four-electron pathway was strongly favored, and the HO_2_^−^ yield approached 0%. Chronoamperometry experiments performed at 0.75 V and 1600 rpm demonstrated high catalyst stability, with only 5.9% of the initial current density (−5.1 to −4.8 mA.cm^−2^) lost after 10,000 s.

In a recent study, three materials produced by the assembly of Co(II) and 2-methylimidazole (typically ZIF-67), nicotinate, and 2,3-pyrazinedicarboxylate ligands (the related MOF is named Co(CO_2_)_2_Pz) were evaluated as cathode catalysts for PEMFCs [94], with the best results obtained for Co(CO_2_)_2_Pz. The above MOF was pyrolyzed at 700 °C and then acid-leached to afford a mesoporous carbon (340 m^2^ g^−1^) doped with Co (3 at%) and N (6.5 at%). As the same group has characterized this catalyst and its O_2_ reduction activity in an earlier study [95], the related results are not repeated here. An MEA was prepared by sandwiching a Nafion membrane between two gas diffusion layers comprising carbon papers coated with anodic (Pt/C) and cathodic (Co(CO_2_)_2_Pz) catalysts. The assembled cell was fed with H_2_/O_2_ and tested at 80 °C, with the maximum power density, limiting current density, and open cell voltage determined as 259 mW cm^−2^ (at 0.25 V), 1.36 A cm^−2^, and 0.86 V, respectively. Under similar conditions, comparable results were obtained for the standard Pt/C catalyst (352 mW cm^−2^ at 0.43 V, 1.23 A cm^−2^, 0.98 V). The fuel cell prototype constructed with Co(CO_2_)_2_Pz could be operated over 500 h without a significant change of electrochemical parameters and Co/Pt leakage.

**Figure 9:**
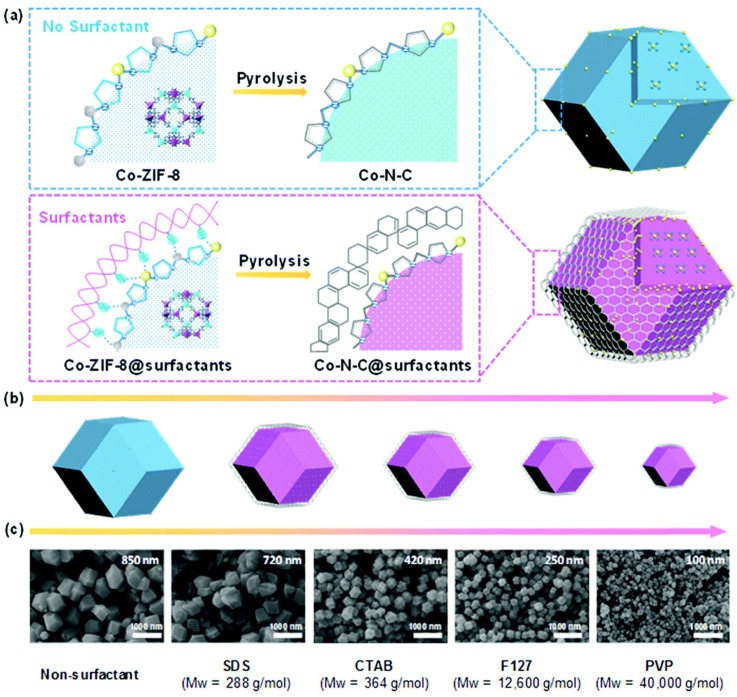
(**a**) In situ confinement pyrolysis strategy used to synthesize core–shell-structured Co-N-C@surfactant catalysts with increased active site density. (**b**) Expected particle sizes and (**c**) SEM images showing the different morphologies and particles sizes of catalysts obtained in the presence and absence of surfactants (SDS, cetrimonium bromide (CTAB), F127, and polyvinylpyrrolidone (PVP)). Reprinted with permission from ref. [96]. Copyright (2018) Royal Society of Chemistry.

An innovative surfactant-assisted MOF approach to core–shell-structured Co-N-C catalysts was reported by Wu et al. [96]. The confinement effect of the surfactant covering ZIF-8 nanocrystals resulted in the formation of a core–shell structure with atomically dispersed Co-N-C@surfactant catalysts and a high density of active sites. Figure 9 illustrates the application of the proposed strategy to the synthesis of core–shell-structured Co-N-C@surfactant catalysts, revealing that among the various surfactants such as sodium dodecyl sulfate (SDS), cetrimonium bromide (CTAB), and PVP, the F127 block copolymer (PEO100-PPO65-PEO100) presented the best results. Co-ZIF-8 particles had a non-uniform rhombododecahedral shape with an average size of 850 nm, while the presence of F127 restricted crystal growth to afford particles with a uniform size of 250 nm (Figure 9c). After carbonization, the initial shape was conserved, and the apparition of a partially graphitized carbon shell was confirmed by HR-TEM and Raman spectroscopy (*I*_D_/*I*_G_ = 1.52). According to DFT calculations, the catalyst contained abundant CoN_2+2_ active sites favoring four-electron reduction. ORR activity was studied in an acidic medium, and the obtained onset (0.93 V) and half-wave (0.84 V) potentials were similar to those of state-of-the-art catalysts. The authors compared their results with those reported in [54] closely related papers, claiming that the new catalyst outperforms previously known PGM- and Fe-free catalysts and thus holds a new record. The negligible (1%) yield of H_2_O_2_ indicated a strong preference for four-electron reduction. In addition, the catalyst showed high stability, probably being the most stable among the catalysts presented herein. Specifically, after 100 h, initial activity retentions of up to 94.5% and 65% were observed when constant potentials of 0.7 and 0.85 V were applied, respectively. Finally, the novel material was used as the cathode catalyst of an H_2_-O_2_ fuel cell, delivering a power density of 0.87 W cm^−2^ with encouraging durability.

**Figure 10:**
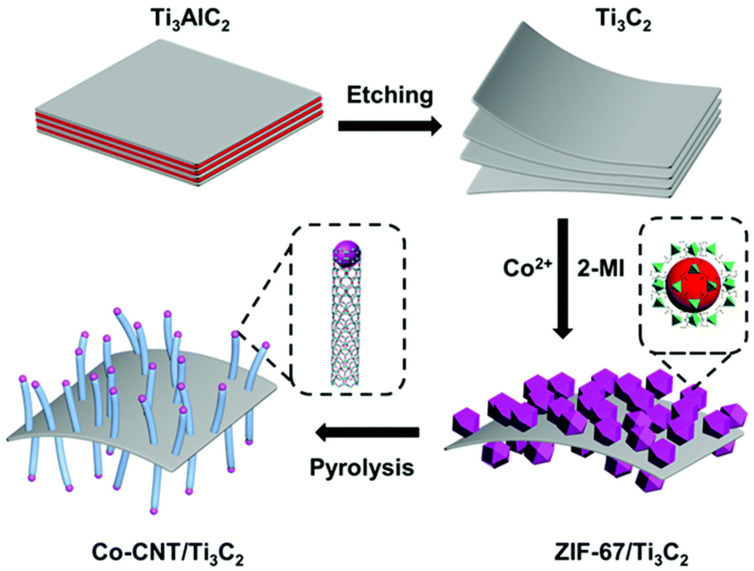
Schematic synthesis of Co-CNT/Ti_3_C_2_. Reprinted with permission from reference [97]. Copyright (2018) Royal Society of Chemistry.

Chen et al. [97] fabricated Co-tipped carbon nanotube/Ti_3_C_2_ nanosheet composites (Co-CNT/Ti_3_C_2_) by the in situ growth of ZIF-67 particles on Ti_3_C_2_ nanosheets followed by thermal treatment (Figure 10). The abundant oxygen-containing functional groups and fluorine on the surface of 2D Ti_3_C_2_ nanosheets facilitated MOF nucleation and growth. SEM imaging revealed the growth of ZIF-67 polyhedra with diameters of tens of nanometers on nanosheets. The presence of metallic Co in Co-CNT/Ti_3_C_2_ was verified by the presence of characteristic Co (111), (200), and (220) peaks at 44.2°, 51.5°, and 75.9°, respectively, in the corresponding XRD pattern. The related high-resolution N 1s spectrum featured a peak of Co-N active sites at 399.6 eV. SEM and TEM imaging showed that Co-CNT/Ti_3_C_2_ was a 2D carbonaceous material with vertically aligned 1D nanotubes on its surface. In addition, abundant 10-nm Co nanoparticles were present on carbon nanotube tips. The amount of Ti_3_C_2_ used during catalyst preparation was found to significantly affect ORR activity. The best-performing specimen featured a half-wave potential of 0.82 V and a *J*_L_ of 5.55 mA cm^–2^, which slightly exceeded the values obtained under identical conditions for Pt/C (0.82 V and 5.30 mA cm^−2^). To shed light on ORR pathways, the H_2_O_2_ yield and n were calculated based on the results of RRDE experiments as <5% and 3.9, respectively, which confirmed that Co-CNT/Ti_3_C_2_ favored the four-electron pathway. In addition, this catalyst showed good stability, featuring a current retention of >90% after a 10,000-s chronoamperometry test, whereas a value of <70% was obtained for commercial Pt/C. Thus, the above catalyst exhibited interesting ORR performance despite featuring some of the lowest specific surface areas (109 m^2^ g^−1^) among the catalysts discussed herein. These results were explained by the abundant Co-N/C sites present on the tips of carbon nanotubes, the high graphitization degree of carbon, and the presence of Ti_3_C_2_ nanosheets that ensured fast electron transfer and facilitated O_2_ diffusion and active site exposure.

**Figure 11:**
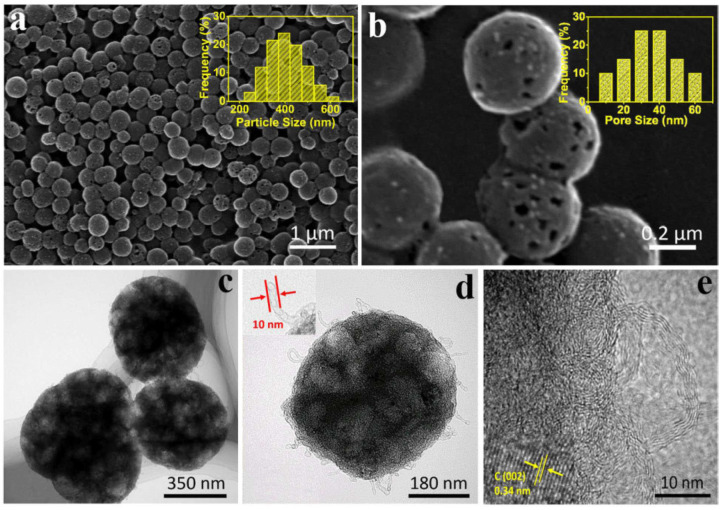
(**a**,**b**) SEM, (**c**,**d**) TEM, and (**e**) HR-TEM images of N/Co-co-doped mesoporous carbon microspheres. Reprinted with permission from ref. [98]. Copyright (2019) John Wiley and Sons.

N/Co-co-doped mesoporous carbon microspheres were synthesized by solvothermal treatment of a 3,6-bis(1-imidazolyl)-1,2,4,5-tetrazine (BIMT) linker with Co(II) nitrate followed by pyrolysis at 800 °C under N_2_ [98]. The above linker was selected because tetrazine/imidazole-containing precursors are more inclined to be transformed into graphitic- and pyridinic-N species, which are active catalytic sites for the ORR. The complexation of Co by BIMT was confirmed by the observation of a typical Co(II)–N peak at 856 cm^−1^ in the IR spectrum of the non-calcined material, while SEM imaging revealed the formation of microspheres with diameters of 400–650 nm (Figure 11). Except for a rougher surface, the catalyst obtained after pyrolysis inherited the precursor architecture, comprising spheres with diameters of 250–600 nm and presenting macropores (≈60 nm) and large mesopores (≈10 nm). Therefore, the calcined material featured a substantial surface area of 381 m^2^ g^−1^, a high pore volume of 1.25 cm^3^ g^−1^, and a low micropore surface area of 14.8 m^2^ g^−1^. The catalyst was concluded to be a truly mesoporous material with well-exposed catalytic sites and ORR-favorable mass transport properties and was compared to Pt/C in both alkaline and acidic media. In 0.5 M H_2_SO_4_, Pt/C exhibited onset and half-wave potentials of 0.852 and 0.671 V, respectively, whereas the respective values of the N/Co-co-doped carbon catalyst equaled 0.863 and 0.707 V. Moreover, the MOF-based catalyst featured a low H_2_O_2_ yield of 5.9% in the 0.1–0.8 V potential window and *n* > 3.91. After a 10,000-s chronoamperometry test carried out in O_2_-saturated 0.5 M H_2_SO_4_ at 0.4 V, Pt/C retained only 65.5% of its initial current density, while the MOF-based catalyst showed a loss of only 5.3%. In addition, the developed catalyst was unaffected by methanol crossover and CO poisoning.

**Figure 12:**
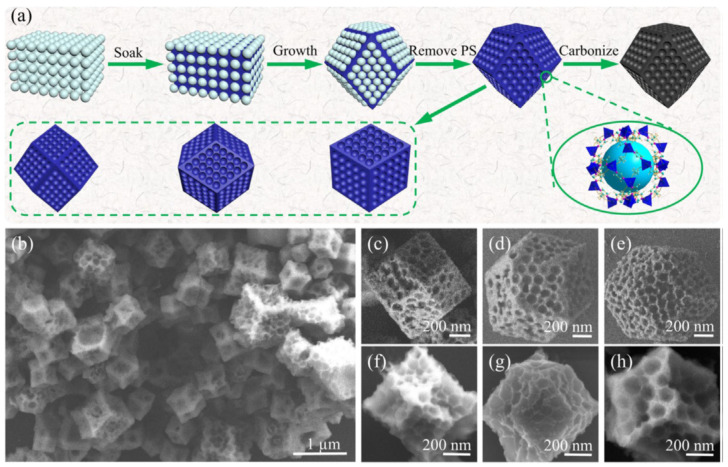
(**a**) Schematic preparation and (**b**,**f**–**h**) SEM images of the Co-supported, N-doped, 3D-ordered porous carbon (Co-NOPC). (**c**–**e**) SEM images of the ordered porous ZIF-67 MOF (OP-ZIF-67) obtained before calcination. Reprinted with permission from reference [99]. Copyright (2020) John Wiley and Sons.

**Figure 13:**
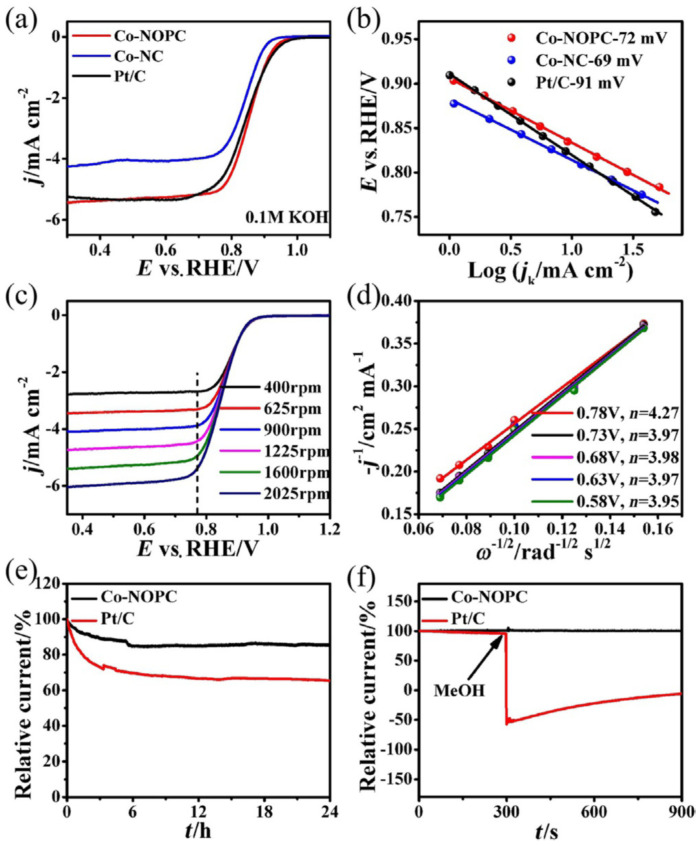
(**a**) ORR polarization curves and (**b**) the correspondinf Tafel plots. (**c**) LSV curves of the MOF-based catalyst (denoted Co-NOPC) at different rotating speeds and (**d**) the corresponding Koutecky–Levich (K-L) plots. (**e**) Chronoamperometric and (**f**) methanol-crossover chronoamperometric responses for Co-NOPC and Pt/C. All tests were performed in 0.1 M KOH. Reprinted with permission from reference [99]. Copyright (2020) John Wiley and Sons.

Recently, ordered porous ZIF-67 (OP-ZIF-67) with accurate control of macro- and microporosity has been prepared through an original approach combining a hard-template method and ultrasonic nucleation [99]. The method relied on the growth of a ZIF-67 MOF in a polystyrene matrix followed by its careful dissolution in THF (Figure 12a). After 2-h pyrolysis under N_2_ at 500 °C, a Co-supported, N-doped, 3D-ordered porous carbon (Co-NOPC) was obtained. SEM imaging (Figure 12) showed that OP-ZIF-67 comprised well-defined rhombododecahedral particles of ≈1 µm and featured ≈110-nm macropores uniformly distributed throughout the single-crystal particles. In the Co-NOPC catalyst, this structure was generally well preserved and was transformed into an interconnected graphitic carbon skeleton, as revealed by XRD. The related pattern showed peaks at ≈23.3 and 43.58°, which were assigned to the (002) and (100) planes of the graphitic structure, respectively. In addition, the absence of Co metal peaks for the catalyst annealed at 500 °C confirmed that nanoparticle formation was avoided. The high-resolution Co 2p_3/2_ spectrum mainly showed two peaks, namely those of Co^2+^ (779.1 eV) and Co^0^ (776.9 eV), and the Co^2+^/Co^0^ peak ratio decreased from 0.915 to 0.686 as the annealing temperature increased from 500 to 700 °C. Together, XRD and XPS results confirmed that Co-N*_x_* active sites preferably formed at 500 °C. In an alkaline medium (see Figure 13a), the onset (0.95 V) and half-wave (0.86 V) potentials as well as the *J*_L_ (5.2 mA cm^−2^) of Co-NOPC were comparable to those of Pt/C (0.98 V, 0.85 V, and 5.3 mA cm^−2^, respectively). RRDE experiments revealed that the average *n* equaled 3.93, while the peroxide yield was under 5% within a wide potential window. Finally, long-term chronoamperometric measurements at 0.86 V demonstrated that after 24 h, the catalyst lost ≈15% of its initial current density, while Pt/C exhibited a loss of 31% (see Figure 13e). Co-NOPC also showed interesting electrochemical performance in an acidic electrolyte. This superior ORR activity originated from the optimized density of Co-N*_x_* active sites and the controlled hierarchical porosity of the catalyst structure, which permitted the fast diffusion of electrolyte/oxygenated species and ensured a high exposure of active sites.

A recent study reported Co-doped ZIF precursors with a wide Co loading range of 0–30 at% [47], attempting to realize a high density of active atomic Co sites uniformly dispersed in a carbon matrix, which remains a grand challenge for enhanced catalytic activity. The catalyst prepared at a Co loading of 20 at% (Co/Co+Zn in the units of molar percentages of the corresponding compounds used for the synthesis of ZIF nanocrystal precursors) presented the best performance and was denoted as 20Co-NC-1100. Uniformly dispersed atomic Co sites were observed throughout the carbon particles obtained after pyrolysis. During TEM imaging, when the electron beam was placed on the observed bright dots, both N and Co were found to be present in the obtained electron energy loss spectrum, which suggested the coexistence of these elements in the form of Co-N*_x_* active sites. Moreover, X-ray absorption spectroscopy demonstrated that Co-N_4_ centers were embedded into the ZIF-derived carbon matrix with the assistance of high-temperature treatment. The related XPS spectrum showed a peak at 399.2 eV, which was attributed to Co-bonded N. HR-TEM imaging confirmed that the Co-doped ZIF catalyst retained its polyhedral particle morphology after annealing and revealed the presence of highly disordered graphitic domains. This feature, ascribed to the inability of atomic Co sites to catalyze the graphitization of carbon, in contrast to metallic Co nanoparticles, was an additional indirect proof of the preferred formation of Co-N*_x_* active sites instead of metal particles. As expected, the prepared catalyst showed very good electrochemical performance and stability. For instance, in 0.5 M H_2_SO_4_, 20Co-NC-1100 exhibited an *E*_1/2_ of 0.80 V, which was only 60 mV lower than that recorded for Pt/C. Finally, after the application of a constant potential of 0.7 V for 100 h, the catalyst retained ≈83% of its initial activity, thus featuring one of the highest ORR activities and the best durability reported for Co-N-C catalysts in challenging acidic media.

In a recent study, the controllable synthesis of MOF-derived Co/N-CNTs (carbon nanotubes) was realized using mesoporous silica layer-covered ZIF-67 nanocrystals [100]. During MOF calcination, the SiO_2_ shell prevented the rapid aggregation of Co nanoparticles in the internal space of ZIF-67, promoting the formation of Co nanocatalysts, and it additionally provided unique external “sieves” to induce the catalytic growth of CNTs. Note that silica was in situ generated by the thermal decomposition of TEOS during calcination under N_2_. The Co/N-CNT catalyst obtained after annealing and HF leaching (to remove SiO_2_) conserved the dodecahedral structure of the parent MOF. The surface of the carbon matrix was sprinkled with CNTs having an average diameter of 13 nm, while the Co nanoparticles were encapsulated by a few layers of graphitic carbon at the CNT tips. RRDE experiments showed that the onset potential for Co/N-CNTs equaled that of Pt/C (−0.005 V vs. Ag/AgCl), while the half-wave potential (−0.154 V vs. Ag/AgCl) and the limiting current density (5.82 mA cm^−2^) were 21 mV more positive and 0.4 mA cm^−2^ larger than those of Pt/C. The average *n* was calculated as ≈3.89, demonstrating that the four-electron pathway was preferred; however, no peroxide yield was provided. Finally, the current density of Co/N-CNTs showed a negligible decay of only ≈3% after a 22000-s chronoamperometric test at a constant potential of −0.35 V vs. Ag/AgCl, whereas a loss of 14% was observed for Pt/C, which suggested the superior stability of the former catalyst.

**Table 4:** Recent research works (2018–2020) dealing with the preparation of Co-based electrocatalysts for fuel cells with a focus on ORR activity. Examples in acidic and alkaline media are provided, and major physicochemical properties and electrochemical performances realized using rotating electrodes are compared.

				RDE/RRDE Experiments ***					
Precursors	Main Catalytically Active Sites	BET Surface Area (m^2^ g^−1^)	N/M (N = Nitrogen; M = metal) Contents (at%) *	ORR Half-Wave Potential (*E*_1/2_ vs. RHE) **	Average n	Peroxide Yield	Cyclability	Methanol Tolerance	Reference
Co/Zn bimetallic ZIF + P123 micelles	Co-N_x_	1539	3.11/0.56	0.1 M HClO_4_: 0.78 (5 mV s^−1^)	0.1 M HClO_4_: 3.88 (0.2–0.5 V)	n/a	0.1 M HClO_4_: after 8.3 h, 87.4% of initial current at 0.45 V	Excellent under acidic conditions	[90]
Co/Zn-ZIF-67	Co-N_x_	1037	n/a	0.5 M H_2_SO_4_: 0.72	0.5 M H_2_SO_4_: 3.90	0.5 M H_2_SO_4_: ≈6% (0.2–0.7 V)	0.5 M H_2_SO_4_: after 8.3 h, 91% of initial current at 0.4 V	Excellent under acidic conditions	[91]
ZnO@ZIF-8 + MAC solution (Ag, Ni, Co, Cu)	Co-N_x_ (the best result)	678.8	n/a	0.80 (5 mV s^−1^)	3.9–4.0	≈4% (0.3–0.7 V)	After 10 h, 97.4% of initial current at 0.6 V	Excellent under alkaline conditions	[92]
Co/Zn-ZIF + C_4_H_11_NO_3_ + dopamine hydrochloride	Co-N_x_	n/a	n/a	0.838 (5 mV s^−1^)	close to 4	≈0% (0.2–0.8 V)	After 2.8 h, 94.1% of initial current at 0.75 V	n/a	[93]
2,3-pyrazinedicarboxylate + CoCl_2_	Co-N_x_	320	6.5/3.0	Related information available in ref. [95]					[94]
Co-ZIF-8 + F127 block copolymer	Co-N_2+2_ + Co-N_4_	825	9.1/1.0	0.5 M H_2_SO_4_: 0.84 (900 rpm and 50 mV s^−1^)	0.5 M H_2_SO_4_: close to 4	0.5 M H_2_SO_4_: <1% (0.2–0.8 V)	0.5 M H_2_SO_4_: after 100 h, 94.5/65% of initial current at 0.7/0.85 V, 200 rpm	n/a	[96]
ZIF-67 + Ti_3_C_2_	Co-N_x_	109	n/a	0.82	3.9 (0.25–0.8 V)	<5% (at 0.5 V)	After 2.8 h, ≈90% of initial current	Excellent under alkaline conditions	[97]
BIMT + Co(NO_3_)_2_	Co-N_x_	381	4.85/0.56	0.872 0.5 M H_2_SO_4_: 0.707	>3.92 0.5 M H_2_SO_4_: >3.91	<3.7% (0.2–0.9 V) 0.5 M H_2_SO_4_: <5.9% (0.1–0.8 V)	After 2.8 h, 94.7% of initial current at 0.4 V	Excellent under acidic conditions	[98]
ZIF-67 + polystyrene matrix	Co-N_x_	418	14.9/4.79	0.86 0.5 M H_2_SO_4_: 0.76	≈3.93 0.5 M H_2_SO_4_: ≈3.89	<5.0% (0.2–0.9 V) 0.5 M H_2_SO_4_: ≈1–7% (0.15–0.75 V)	After 24 h, ≈85% of initial current at 0.86 V 0.5 M H_2_SO_4_: after 24 h, ≈64% of initial current	Excellent under alkaline and acidic conditions	[99]
ZIF-67	Co-N_x_	565	3.56/0.34	0.5 M H_2_SO_4_: 0.80 (900 rpm and 5 mV s^−1^)	n/a	0.5 M H_2_SO_4_: <5% (0.15–0.85 V)	0.5 M H_2_SO_4_: after 100 h, 83% of initial current at 0.7 V, 200 rpm	n/a	[47]
ZIF-67 + TEOS	Co nanoparticles	n/a	6.56/0.86	−0.154 vs. Ag/AgCl	≈3.89	n/a	After 6.1 h, 97% of initial current at −0.35 V vs. Ag/AgCl	Good under alkaline conditions	[100]

* Sometimes, weight percentage was used, and hence, “wt %” data are provided. More than one metal or heteroatom can be provided depending on the active sites involved. ** Potentials are referenced to RHE except when stated otherwise. The ORR performance values were evaluated at 1600 rpm and 10 mV s^−1^ except when stated otherwise. *** All the ORR performance values listed were measured in 0.1 M KOH except when stated otherwise.

### 3.5. Multi-Metal Electrocatalysts

Another bimetallic MOF was proposed by Jose et al. [101], who first assembled ZIF-8@ZIF-67 as a core–shell material featuring structural stability due to the isoreticular structures and similar unit cell parameters of these two MOF, and spherical aggregates consisting of mesoporous frameworks with nanoparticles were obtained after the successive adsorption of Fe and melamine in the material pores and pyrolysis. XRD revealed that the pyrolyzed material contained metallic Co and CoO embedded into a carbon support, while the related Fe 2p core-level spectrum showed the presence of Fe^3+^ species and featured a dominant peak at ≈710.9 eV, which was assigned to N-coordinated Fe. In 0.1 M KOH, the catalyst presented an onset potential of 1.01 V and a half-wave potential of 0.868 V, which were superior to those of Pt/C (1.00 and 0.857 V, respectively). The ZIF-derived catalyst retained 94.6% of its initial current density after 12,000 s at a constant potential of 0.7 V in an alkaline electrolyte, exhibiting higher stability than Pt/C (84%) as well as an excellent methanol tolerance.

Fe-Co/MIL-101(Cr) hybrid catalysts with various Fe/Co molar ratios were prepared by a facile and mild impregnation method [102]. MIL-101(Cr) with octahedral morphology and a particle size of 500–560 nm was first synthesized; then, its pores were filled with Co and Fe nitrates. In contrast to the above examples, the impregnated material was not pyrolyzed but rather used as prepared. XRD showed that the MOF crystal structure was not affected by Fe and Co loadings, as the positions of the main characteristic peaks remained unchanged. Moreover, no crystalline Co or Fe species were detected, which suggested that these elements were present as ions uniformly dispersed within the MOF matrix (specific surface area = 1520 m^2^ g^−1^). The synergistic effect between Fe and Co species contributed to remarkable ORR catalytic activity. The MOF synthesized using equimolar quantities of Fe and Co exhibited the best ORR activity, favoring the four-electron transfer pathway and featuring an onset potential of −0.17 V (vs. Ag/AgCl), and a half-wave potential of −0.28 V in 0.1 M KOH, which were slightly lower than the values obtained for Pt/C. In contrast, the above catalyst exhibited a higher cycling stability (current retention of 85.4% after 20,000 s at a constant potential of −0.4 V vs. Ag/AgCl) than Pt/C (70% under the same conditions).

A highly active electrocatalyst containing Cu/Co- and N-co-doped porous carbon structures was prepared and characterized by Wang et al. [103]. Freshly prepared Co-ZIF-67 was dispersed in a solution of Cu(NO_3_)_2_ in an ultrasonic bath at different ZIF-67/Cu(NO_3_)_2_ ratios. The best results were obtained for a ZIF-67/Cu(NO_3_)_2_ weight ratio of 2.5, and the final catalyst, obtained after pyrolysis at 900 °C under N_2_, was denoted as Cu/Co/N-C#2. TEM imaging showed that ZIF-67 comprised hexagonal prisms with a size of 1.3 µm, whereas a hollow structure with a rougher surface was obtained after Cu-doping. Energy-dispersive spectroscopy (EDS) mappings revealed that MOF particles were surrounded by Cu-containing species. TEM imaging of the pyrolyzed material allowed its lattice spacings to be determined as 0.21 and 0.34 nm, which corresponded to metallic Co (111) and graphitic carbon (002) planes, respectively. Interestingly, XRD revealed the presence of Cu_0.15_Co_2.84_O_4_ nanoparticles that acted as new active sites for the ORR. When electrochemically tested in neutral phosphate buffer saline (PBS) solution, the catalyst exhibited onset and half-wave potentials (0.25 and 0.14 V vs. Ag/AgCl, respectively) exceeding those of Pt/C (0.24 and 0.12 V vs. Ag/AgCl, respectively) and presented a higher *J*_L_ than Pt/C (6.92 vs. 6.72 mA cm^−2^ at −0.6 V vs. Ag/AgCl). The related Nyquist plot showed that the MOF-based electrocatalyst had a lower charge transfer resistance, which was indicative of enhanced ORR activity and was mainly ascribed to the pyrolytic production of graphitized carbon to improve electrical conductivity. However, the initial current density sharply decreased by 6.8% upon the addition of methanol, i.e., the methanol tolerance was not as high as expected. In addition, Cu/Co/N-C#2 showed better long-term stability than Pt/C, featuring an initial current reduction of 15.4% (24.9% for Pt/C) after 8000 s. The superior performance of this new bimetallic Cu/Co-doped porous carbon was attributed to its high surface area and the high nitrogen content engendered by the mixed coordination with Cu and Co ions, which provided various active sites for the ORR (Cu-N*_x_*, Co-N*_x_*-, pyridinic- and graphitic-N, and Cu_0.15_Co_2.84_O_4_).

A high-performance core–shell-structured N-doped CoCx/FeCo@C/reduced graphene oxide (rGO) hybrid was reported as a new bifunctional catalyst for the ORR and the oxygen evolution reaction (OER) [104]. The related synthesis was fairly long and involved successive reactions between the different precursors listed in Table 5 as well as the preparation of GO followed by 4-h pyrolysis at 800 °C under Ar. TEM imaging of the Fe-doped Co_3_[Co(CN)_6_]_2_ intermediate revealed a cubic structure with an average particle diameter of 60 nm. The XRD pattern of N-doped CoCx/FeCo@C/rGO obtained after pyrolysis showed the presence of an FeCo alloy and CoCx, confirming the successful synthesis of the above hybrid. The related C 1s core-level spectrum showed peaks at 285.03 and 286.43 eV, which were attributed to C–N and C–O bonds, respectively, and confirmed the successful doping of nitrogen. In addition, the peaks at 705.70 and 716.97 eV in the Fe 2p spectrum were assigned to Fe^0^ (metallic iron) in FeCo alloys. TEM analysis revealed a wrinkled core–shell structure with a particle size of ≈100 nm and CoCx well wrapped by the carbon membrane. The different catalytic sites (FeCo and CoCx), as well as the N-doped rGO and the carbon shell, contributed to enhanced the ORR activity, which was characterized by an onset potential of 1.0183 V (Pt/C = 1.0174 V) and a half-wave potential of 0.9653 V (Pt/C = 0.9213 V). Chronoamperometry experiments showed that after 5000 s, N-doped CoCx/FeCo@C/rGO preserved 95.9% of its initial current (cf. 74.3% for Pt/C). This high long-term stability was attributed to the presence of a carbon shell that inhibited the possible catalyst corrosion. Excellent *n* values of 3.8–3.95 (between 0.52 and 0.8 V) and a peroxide yield of 8% were obtained. These results suggested that FeCo alloys play a crucial role in the electrocatalytic reaction, in line with the known fact that that the O–O bond easily breaks because of the strong affinity of Fe to O.

Niu and Yang synthesized mesoporous N-doped graphene (MNG) compounds using amorphous ZIF-67 fibers as a precursor [105]. Specifically, ZIF-67 formed on a cubic NaCl substrate was carbonized in an atmosphere of N_2_, which was followed by the dissolution of NaCl in an acidic solution and washing with deionized water. MNG-CoFe, a catalyst with a sheet-like morphology, was fabricated using Fe-doped amorphous ZIF-67 as a MOF precursor to generate MNG encapsulating CoFe nanoparticles. HR-TEM imaging showed that well-organized graphene layers were only observed on the metal surface, implying the assistance of metal nanoparticles in the growth of graphene layers during pyrolysis. Unfortunately, the authors did not report the BET surface area of their catalyst, although it is an important physical property. EDS mapping showed that C and N were distributed throughout the entire sample, with only scattered Co and Fe signals detected within graphene layers, while XRD confirmed the presence of CoFe alloys. Electrochemical performance was assessed in 0.1 M KOH, and the onset potential of MNG-CoFe (0.98 V) exceeded that of Pt/C (0.97 V), while the *J*_L_ values of the two catalysts were similar. The addition of KSCN to the electrolyte did not affect the LSV curve, which confirmed that metal-N/C active sites were not involved in ORR catalysis. Thus, nitrogen dopants and graphene-encapsulated CoFe alloys were concluded to be the active sites for the ORR. Finally, MNG-CoFe showed excellent methanol tolerance and presented a better durability than Pt/C, delivering ≈93% of its initial current density after a 30,000-s chronoamperometric measurement (77% for Pt/C after 10,000 s).

A Fe-N and Co-N homogeneously doped carbon framework was synthesized by the pyrolysis of a cage-encapsulated precursor [106]. First, Fe-Co/Zn ZIF was prepared through a solvothermal reaction between Zn(NO_3_)_2_, Co(NO_3_)_2_, Fe(acac)_3_, and 2-methylimidazole. During the Co/Zn ZIF assembly, the Fe precursor was encapsulated in one cavity or pore of the ideal model. The FeCo-NC catalyst obtained after pyrolysis retained the dodecahedral particle shape of the parent MOF, and TEM imaging revealed the absence of obvious Co or Fe particles, suggesting that these elements were well dispersed and doped into the carbon framework. The obtained catalyst featured a high BET specific surface area of 647.6 m^2^ g^−1^ and contained Fe-N and Co-N active sites (according to XPS analysis), which were expected to impart high ORR activity. Electrochemical performance was studied in alkaline (0.1 M KOH) and acidic (0.1 M HClO_4_) media. LSV measurements showed that in 0.1 M KOH, FeCo-NC presented a half-wave potential (0.855 V) comparable to that of Pt/C (0.827 V), while the current density reached 5.6 mA cm^−2^ (5.0 mA cm^−2^ for Pt/C). The electron transfer number was close to four (*n* = 3.9), and the yield of H_2_O_2_ in the potential range of 0.1–0.6 V was below 4%. The long-term stabilities of FeCo-NC and Pt/C catalysts were assessed by chronoamperometric measurements at 0.57 V during 11000 s. As a result, FeCo-NC retained ≈90% of its initial current density, while Pt/C suffered a loss of 31%. However, the electrochemical performance of FeCo-NC in the acidic electrolyte was significantly inferior to that of Pt/C, and the former catalyst also favored both four- and two-electron reduction under these conditions.

An MOF-74 precursor assembled from Fe/Mn and a cheap organic ligand (2,5-dihydroxyterephthalic acid) were combined with melamine and heated at 800 °C in a flow of N_2_ to generate a core–shell catalyst denoted as Fe_3_Mn_1_/N-CNTs-100 [107]. SEM and TEM imaging showed that many 80-nm-diameter CNTs were formed during pyrolysis. In addition, metallic nanoparticles were observed throughout the carbon matrix, with 50–60% of these particles (average diameter = 26.0 ± 2.3 nm) incorporated inside CNTs. The lattice constant of the incorporated particles (≈0.206 nm) was consistent with the formation of Fe-Mn alloys. Moreover, XRD revealed the presence of crystalline metallic Fe and Fe_2_N. The BET surface area was not as high as expected (104.2 m^2^ g^−1^) because of the blockage of some channels inside CNTs by Fe-Mn alloys. In 0.1 M NaOH, Fe_3_Mn_1_/N-CNTs-100 showed slightly higher ORR performance than commercial Pt/C, exhibiting a half-wave potential of 0.865 V and a limiting current density of 5.33 mA cm^−2^ (0.855 V and 5.27 mA cm^−2^ for Pt/C, respectively). At potentials of 0.25–0.45 V, *n* equaled 3.95, suggesting that Fe_3_Mn_1_/N-CNTs-100 favored four-electron oxygen reduction. When the durability of both catalysts was tested by imposing a constant voltage of 0.8 V for 10,000 s, Fe_3_Mn_1_/N-CNTs-100 maintained 96.1% of the initial current density, while the related value of Pt/C was as low as 65%.

**Figure 14:**
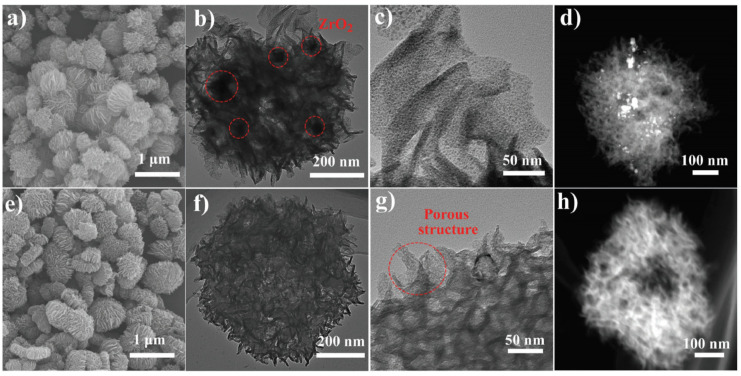
(**a**) SEM, (**b**,**c**) TEM, and (**d**) high-angle annular dark-field STEM (HAADF-STEM) images of the N-GCF-800/ZrO_2_ intermediate catalyst obtained before etching with HF. (**e**) SEM, (**f**) and (**g**) TEM, and (**h**) HAADF-STEM images of the N-GCF-800 catalyst. Reprinted and modified with permission from reference [108]. Copyright (2018) John Wiley and Sons.

A novel Zr–porphyrin MOF hierarchical nanoframework (ZPHNF) assembled from nanosheets was pyrolyzed to generate an N-doped hierarchical graphitic carbon nanoframework with M-N_4_ units [108]. The ZPHNF was synthesized through a solvothermal reaction of ZrCl_4_, Ni- and Fe-tetrakis(4-carboxyphenyl)-porphyrin (Ni-TCPP and Fe-TCPP), benzoic acid, and traces of water in dimethylformamide (DMF). SEM imaging demonstrated that the ZPHNF MOF possessed a uniform 3D framework structure (diameter ≈600 nm) with numerous 2D nanosheets, which were found to be as thick as five layers of Zr_6_ secondary building units according to HAADF-STEM. The product obtained after pyrolysis in N_2_ at 800 °C inherited the general structure and contained abundant ZrO_2_ particles (average size = 5 nm) densely embedded onto nanosheets. These nanoparticles were further removed by HF treatment to obtain the final N-GCF-800 catalyst with a BET surface area of 557 m^2^ g^−1^. The SEM images of the catalyst before and after etching with HF are presented in Figure 14. In alkaline media, N-GCF-Ni9Fe1-800 featured a remarkably enhanced ORR activity compared to that of Pt/C, displaying *E*_1/2_ = 0.83 V and a limiting current density of 6.19 mA cm^−2^ (cf. 0.82 V and 5.57 mA cm^−2^ for Pt/C, respectively). The value of *n* in the potential range of 0.45–0.6 V was calculated as ≈3.99, indicating an ideal four-electron ORR process. Moreover, N-GCF-Ni9Fe1-800 featured excellent methanol tolerance. After 10,000 voltammetric cycles within 0.6–1.1 V, no variance in *E*_1/2_ was noted, and only a slight (0.2 mA) loss of *J*_L_ was observed, suggesting outstanding catalyst stability.

**Figure 15:**
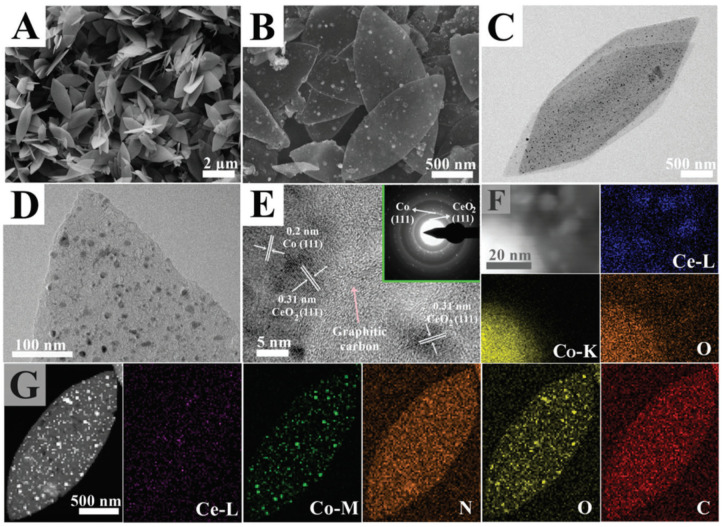
(**A**,**B**) FESEM and (**C**,**D**) TEM images of the ceria@2D-hexagonal-leaf-like hierarchical porous carbon nanosheet (Ce-HPCN) catalyst. (**E**) HR-TEM image showing the presence of Ce/Co in the Ce-HPCN catalyst (inset in (**E**): Selected area electron diffraction (SAED) pattern of Co and CeO_2_) and (**F**) the corresponding STEM-EDS mapping images. (**G**) STEM and elemental mapping images showing the homogenous distribution of Ce, Co, N, C, and O in a leaf-like lamella. Reprinted with permission from reference [109]. Copyright (2018) Royal Society of Chemistry.

Novel ceria@2D-hexagonal-leaf-like hierarchical porous carbon nanosheet (Ce-HPCN) catalysts were synthesized via the carbonization of monoclinic Ce-modified 2D-hexagonal-leaf-like ZIF lamellae (Ce-ZIF-L) [109]. Ce-ZIF-L, obtained by a reaction of Co and Zn nitrates with 2-methylimidazole (ligand) and a Ce source (Ce_2_(OH)_4_SO_4_·2H_2_O) in water, was calcined at 850 °C in Ar to yield the Ce-HPCN catalyst. SEM and TEM imaging (Figure 15) showed that the leaf-like ZIF lamellae had a smooth surface and a uniform average size of ≈2 µm. After pyrolysis, the structure was inherited, and many nanoparticles were detected on the surface of the Ce-HPCN catalyst. Selected area electron diffraction (SAED) confirmed the coexistence and homogenous dispersion of nanosized Co and CeO_2_ species (Figure 15E). In addition, elemental mapping revealed that Ce, Co, N, C, and O were homogeneously distributed in leaf-like lamellae (Figure 15G). In 0.1 M KOH, the onset potentials of Ce-HPCN and Pt/C were obtained as 0.923 and 0.934 V, respectively, whereas the respective half-wave potentials equaled 0.831 and 0.846 V. Moreover, Ce-HPCN featured a low peroxide yield of <5.6% and *n* ≈ 3.87. After 1000 voltammetric cycles between 0 and 1.2 V, no variance in *E*_1/2_ was observed, and only a 2.6% decay of the limiting current density was recorded. The excellent performance of the above catalyst were attributed to the synergistic effect of Co-N*_x_* active sites with CeO_2_, which acted as an oxygen buffer owing to the coexistence of the reversible Ce^4+^/Ce^3+^ redox couple. Moreover, this study presented one of the rare examples of Ce-based electrocatalysts.

Ni_3_ZnN hollow microspheres were synthesized by the nitridation of NiO/ZnO precursors in an atmosphere of NH_3_ [110]. These precursors, composed of 1-µm spheres, were prepared through the reaction of terephthalic acid with Zn^2+^/Ni^2+^ cations under solvothermal conditions. Then, the hollow microspheres were transformed into NiO/ZnO through rapid annealing in air and finally converted into a novel ternary nitride (Ni_3_ZnN) by nitridation in NH_3_. The identity of Ni_3_ZnN was confirmed by XRD, while XPS revealed the presence of N–Zn and N–Ni bonds. SEM and TEM imaging showed that the non-heat-treated MOF comprised regular 5-µm sea urchin-like hierarchical microspheres. After annealing in air and NH_3_, the microspheres were preserved, although their size decreased to 1 µm. The performance of the above catalyst in 0.1 M KOH was inferior to that of the benchmark Pt/C. For instance, Ni_3_ZnN featured an onset potential of 0.81 V, which was 170 mV lower than the value obtained for Pt/C. In addition, *J*_L_ also remained much lower than that of Pt/C. The average *n* was calculated as 2.49 in the 0.3–0.6 V potential window, demonstrating that the ORR largely proceeded through the two-electron pathway. After 2000 CV cycles between 0.6 and 1.2 V at a scan rate of 100 mV s^−1^, the Ni_3_ZnN catalyst showed a 14-mV negative shift in its *E*_1/2_. Unfortunately, the authors did not compare the stability of their catalyst with that of Pt/C.

**Table 5:** Recent research works (2018–2020) dealing with the preparation of multi-metal electrocatalysts for fuel cells with a focus on ORR activity. Examples in acidic and alkaline media are provided, and major physicochemical properties and electrochemical performances realized using rotating electrodes are compared.

				RDE/RRDE Experiments ***					
Precursors	Main Catalytically Active Sites	BET Surface Area (m^2^ g^−1^)	N/M (N = Nitrogen; M = Metal) Contents (at%) *	*E*_1/2_ (V vs. RHE) **	Average n	Peroxide Yield	Cyclability	Methanol Tolerance	Reference
ZIF-8@Fe-ZIF-67 + melamine	Fe-N_x_ + Co/CoO nanoparticles	217	n/a	0.868 (5 mV s^−1^)	near 4.0 (0.4–0.55 V)	n/a	After 3.3 h, 94.6% of initial current at 0.7 V	Excellent under alkaline conditions	[101]
MIL-101(Cr) + Co/Fe nitrates	Co/Fe species	1520	No nitrogen, Fe/Co (1.05/1.03)	−0.28 vs. Ag/AgCl (5 mV s^−1^)	3.695	≈10% (0.3–0.8 V vs. Ag/AgCl)	After 5.6 h, 85.4% of initial current at −0.4 V vs. Ag/AgCl	Excellent under alkaline conditions	[102]
Co-ZIF-67 + Cu(NO_3_)_2_	Co-N_x_ + Cu-N_x_ + Cu_0.15_Co_2.84_O_4_ nanoparticles	286	5.27/4.73(Co) + 4.62(Cu)	0.05 M PBS solution: 0.14 vs. Ag/AgCl	n/a	n/a	0.05 M PBS solution: after 2.2 h, 84.6% of initial current	Sharp 6.8% loss of initial current density upon methanol addition	[103]
K_3_[Co(CN)_6_] + PVP + Co(CH_3_COO)_2_ + FeCl_3_ + GO	FeCo alloy + CoC_x_	68.09	n/a	0.9653 (5 mV s^−1^)	3.8–3.95	8% (0.52–0.8 V)	After 1.4 h, 95.9% of initial current	n/a	[104]
ZIF-67 + NaCl	Co-N_x_ + Fe-N_x_ + CoFe nanoparticles	n/a	6.8/1.1(Co) + 0.8(Fe)	0.98 (5 mV s^−1^)	n/a	n/a	After 8.3 h, 93% of initial current	Excellent under alkaline conditions	[105]
Fe(acac)_3_ + Co/Fe nitrates + 2-mIm	Co-N_x_ + Fe-N_x_	647.6	2.94/0.31(Co) + 0.54(Fe)	0.855 0.1 M HClO_4_: ≈0.7	≈3.9 0.1 M HClO_4_: ≈3.2	<4% (0.1–0.6 V) 0.1 M HClO_4_: 45% (0.2–0.5 V)	After 3.1 h, 90% of initial current at 0.57 V	Excellent under alkaline conditions	[106]
MOF-74 + melamine	Fe-N_x_ + Fe/Fe_2_N nanoparticles + Fe-Mn alloys	104.2	4.34/0.54(Fe) + 0.34(Mn)	0.1 M NaOH: 0.865 0.1 M HClO_4_: 0.57	0.1 M NaOH: 3.95 (0.25–0.45 V)	n/a	0.1 M NaOH: after 2.8 h, 96.1% of initial current at 0.8 V	Excellent under alkaline conditions	[107]
Ni-TCPP + Fe-TCPP + ZrCl_4_	Ni-N_4_ + Fe-N_x_	557	3.64/0.19(Fe) + 0.54(Ni)	0.83 (5 mV s^−1^)	≈3.99 (0.2–0.8 V)	n/a	Stability experiments realized by cyclic voltammetry (100 mV s^−1^, 0.6–1.1 V, 10,000 cycles): no variance in *E*_1/2_ value	Excellent under alkaline conditions	[108]
2-mIm + Zn/Co nitrates + Ce_2_(OH)_4_SO_4_	CeO_2_ + Co-N_x_	n/a	n/a	0.831	≈3.87	<5.6% (0.0–0.8 V)	Stability experiments realized by cyclic voltammetry (50 mV s^−1^, 0.0–1.2 V, 1000 cycles): 97.4% of initial current	Excellent under alkaline conditions	[109]
PTA + Zn/Ni nitrates	Ni_3_ZnN	n/a	n/a	0.81 (onset potential)	2.49 (0.3–0.6 V)	n/a	Stability experiments realized by CV (100 mV s^−1^, 0.6–1.2 V, 2000 cycles): negative *E*_1/2_ shift of 14 mV	n/a	[110]

* Sometimes, weight percentage was used, and hence, “wt %” data are provided. More than one metal or heteroatom can be provided depending on the active sites involved. ** Potentials are referenced to RHE except when stated otherwise. The ORR performance were evaluated at 1600 rpm and 10 mV s^−1^ except when stated otherwise. *** All the ORR performance values listed were measured in 0.1 M KOH except when stated otherwise.

### 3.6. Multi-Site Electrocatalysts

An ingenious two-step strategy for the fabrication of an electrocatalyst containing multifarious Co species embedded in N-rich nanocarbons (Co-N-C) was recently reported [111]. In the first step, Co ions were immobilized by coordination with 1H-imidazo[4,5-f][1,10]phenanthroline (Hip), and the product (denoted as Co-Hip) with abundant Co, C, and N sources was pyrolyzed at various temperatures, with the best results obtained at 800 °C. TEM imaging showed that Co-Hip MOF comprised long and thin nanoribbons, whereas the pyrolyzed material was a porous carbon with metal nanoparticles of 40–100 nm, exhibiting high crystallinity and a lattice spacing of 0.228 nm corresponding to the (020) plane of Co_2_N. The carbon matrix contained numerous pores necessary for electrolyte and gas diffusion to the active sites, although the specific surface area was fairly low (170.26 m^2^ g^−1^). EXAFS measurements confirmed the catalyst composition and revealed the existence of various Co species such as single-atom Co-N_4_ complexes (≈15%), metallic Co (≈55%), and Co_2_N (≈30%). In an alkaline electrolyte, the Co-N-C catalyst and Pt/C featured half-wave potentials of 0.82 and 0.84 V, respectively, delivering similar limiting current densities of 5.32 mA cm^−2^. The values of *n* calculated at various potentials were all close to 4.0, and good stability under a constant potential as well as excellent methanol tolerance were observed. The excellent performance of the above catalyst was attributed to the coexistence of Co_2_N and single-atom Co (Co-N_4_) sites.

A novel metal-doped porous carbon was prepared by Wang et al. through the solvothermal synthesis of ZIF-8 and MIL-100(Fe) followed by heat treatment of their mixture in N_2_ at 1000 °C and subsequent exposure to NH_3_ during cooling [112]. XRD revealed the presence of graphitic carbon as well as metallic Fe and Fe_3_C species, as follows from the detection of peaks at 43.6 and 44.5°. SEM imaging showed strongly bent carbon nanosheets with tunnels as well as some CNTs, which helped to increase catalyst conductivity and create more surface area for contact with the electrolyte. HR-TEM investigations were performed to determine the composition of the particles present throughout the carbon matrix. The interspacings of 0.203 and 0.238 nm were consistent with the (110) lattice fringe of metallic Fe and the (210) lattice fringe of Fe_3_C, respectively. In addition, the N 1s core-level spectrum of the catalyst displayed a peak ascribed to Fe-N active sites (399.3 eV). This catalyst showed impressive ORR activity in an alkaline medium, featuring an *E*_1/2_ improvement of 50 mV over 20 wt % Pt/C. However, in 0.5 M H_2_SO_4_, the half-wave potential (0.79 V) and limiting current density of (−4.38 mA cm^−2^ at 0.2 V) were slightly lower than those of Pt/C (0.85 V and −4.40 mA cm^−2^, respectively). Peroxide yields of 0.78–10.40% were obtained in both alkaline and acidic media, with the average *n* of 3.90 being very close to that of Pt/C (3.95). A high current density retention of 92.2% after 20,000 s was obtained for the MOF-based catalyst, whereas Pt/C featured a lower value of 70.5%.

A recent work has described a new family of carbon-rich benzimidazole-based MOF precursors equipped with various functional groups to modulate their electronic and ligating properties [113] and prepared from FeCl*_3_* and 5,6-disubstituted 1*H*-benzo[*d*]imidazoles (substituent = OH, COOH, or OCH_3_) as ligands. The best MOF was prepared in the case of substituent = OH and pyrolyzed at 900 °C to yield a material (denoted as TAL-1-900) comprising dense ≈15-nm α-Fe/Fe_3_C@C nanocrystals embedded into onion-like carbon shells. XPS revealed the presence of graphitic/pyrrolic/pyridinic N species as well as Fe-N*_x_* active sites, which are all required for high ORR/OER activities. Although TAL-1-900 presented a relatively low specific surface area of 380 m^2^ g^−1^ with N and Fe contents of only 0.6 at% and 0.9 wt %, respectively, it showed fairly good ORR performance. According to the results of RDE measurements in O_2_-saturated 0.1 M KOH, the onset and half-wave potentials of TAL-1-900 (1.01 and 0.87 V, respectively) were similar to those observed for Pt/C (1.01 and 0.85 V, respectively). The value of *n* was close to four, indicating that oxygen was preferentially reduced to water. After continuous cycling from 0.6 to 1.0 V for 5000 cycles, the electrocatalytic activity of TAL-1-900 remained unchanged, as exemplified by the almost unaffected onset and half-wave (Δ*E*_1/2_ = 6.1 mV) potentials and a slight decrease in *J*_L_, which indicated that cycling did not strongly affect catalyst porosity.

A hybrid Fe_3_C@N,S-co-doped CNT–coated porous carbon has recently been fabricated from an isoreticular metal–organic framework-3 (IRMOF-3), melamine, sodium dodecylbenzenesulfonate (SDBS), and Fe(NO_3_)_3_·9H_2_O [26]. IRMOF-3 was synthesized via a solvothermal reaction and ultrasonicated in ethanol in the presence of other precursors. The Fe-N-S/C catalyst was obtained after pyrolysis in a flow of N_2_ at 900 °C and acid leaching. SEM investigations showed that the cubic structure of IRMOF-3 was retained after decoration, and a porous carbon matrix with bamboo-like CNTs and homogeneous dispersion of S, N, and Fe was obtained after pyrolysis. XRD confirmed the presence of graphitic carbon and Fe_3_C and the absence of iron oxide. The high specific surface area (267 m^2^ g^−1^) and the mesoporous structure of this catalyst facilitated the transport of reactants and electrons, which is a pre-requisite for high electrocatalytic activity. RDE experiments performed at 900 rpm in 0.1 M NaOH showed that the Fe-N-S/C catalyst presented a higher limiting current density (4.43 mA cm^−2^) than Pt/C (4.19 mA cm^−2^) and a half-wave potential of 0.89 V, indicating the synergistic effect between S-doped carbon and Fe_3_C groups. Moreover, *n* = 3.9 was obtained at 0.4 V. Chronoamperometric measurements at a constant voltage of 0.8 V showed that the Fe-N-S/C catalyst lost only 5.7% of its initial current density after 10,000 s, whereas Pt/C lost 33%. Finally, the current density of Fe-N-S/C remained steady before and after the addition of methanol, which indicated high methanol tolerance in an alkaline electrolyte. The superior activity was ascribed to the porous carbon, high content of active N-species, and the synergistic effect between Fe_3_C nanoparticles and S dopants.

Fe-, N-, and S-co-doped carbon matrix/CNT nanocomposites (Fe-N-S CNNs) were prepared by the pyrolysis of ZIF-8 impregnated with FeSO_4_ and hydrazine [114]. ZIF-8 impregnated with different amounts of FeSO_4_ maintained an angular octahedral morphology (diameter ≈ 60 nm) but was converted into a composite with a carbon matrix/nanotube 3D nanostructure after pyrolysis. CNTs of 8-nm diameter were embedded in the carbon matrix. The material with the best ORR activity was prepared at a ZIF-8/FeSO_4_ weight ratio of 6% and presented a high surface area of 974 m^2^ g^−1^. HAADF-STEM mapping confirmed that C, N, O, Fe, and S were uniformly distributed on the carbon matrix. In slight contrast to other research works discussed in this review, the presence of C-S-C active sites was confirmed by XPS. Specifically, the observed S 2p peaks (163.9 and 165 eV) were attributed to thiophene-S functions, which are known to collaborate with N-doped sites to promote ORR activity. Very good electrochemical performance was observed in 0.1 M KOH and 0.5 M H_2_SO_4_; e.g., a high half-wave potential of 0.91 V was obtained in alkaline conditions (cf. 0.85 V for Pt/C). The H_2_O_2_ yield was in the range of 1.8–5%, and *n* equaled 3.95. Long-term stability experiments showed that the MOF-based catalyst lost ≈5.21% of its initial current density after 12 h, whereas a decrease of 29.05% was observed for Pt/C after only 4 h. The combination of high surface area, good conductivity, abundant Fe-N*_x_* and C-S-C active sites, and transport channels makes the above material a promising catalyst for ORR in both alkaline and acidic media.

The hydrothermal reaction of a Co salt in the presence of two ligands, 4-pyridyl-tetrathiafulvalene-4-pyridyl (4-py-TTF-4-py) and terephthalic acid (PTA), has been used to prepare a novel MOF (denoted as Co@NSC/MWCNTs), which was further dispersed in a solution of multi-walled carbon nanotubes (MWCNTs) and calcined at 750 °C under N_2_ [115]. SEM/TEM imaging revealed a MWCNT mat featuring random, curled, and intertwined CNTs with diameters of 10–20 nm. Rectangular MOF crystals of ≈5–20 nm size were immobilized on the surface of MWCNTs, and calcination afforded Co nanoparticles embedded into an N/S co-doped graphitic carbon layer (1–3-nm-thick) supported on MWCNTs. The high-resolution Co 2p spectrum of Co@NSC/MWCNTs exhibited peaks at 780.8 and 783 eV, which were ascribed to the Co 2p_1/2_ and Co 2p_3/2_ transitions of metallic Co, respectively, while the peak at 283.9 eV in the C 1s core-level spectrum revealed the presence of C–S–C bonds and suggested the incorporation of sulfur into the carbon matrix. RDE experiments showed that compared to 20 wt % Pt/C (*E*_onset_ = 0.962 V, *E*_1/2_ = 0.854 V), the novel catalyst showed slightly lower onset and half-wave potentials of 0.843 and 0.782 V, respectively, but it presented a higher *J*_L_ (−5.49 mA cm^−2^ at 0.5 V vs. −5.14 mA cm^−2^ for Pt/C), thus featuring higher electrocatalytic activity. In an alkaline medium, Co@NSC/MWCNTs favored the four-electron oxygen reduction pathway, with *n* oscillating between 3.82 and 3.97 V and the peroxide yield equaling ≈5–8% in a potential range of 0.3–0.7 V. Catalyst durability was also evaluated by chronoamperometric response measurement at a constant voltage of 0.692 V during 10,000 s. After this measurement, Co@NSC/MWCNT retained 96.5% of the initial current density, while Pt/C showed a loss of 17% under the same conditions.

A MIL-101(Fe)/C catalyst was prepared through the solvothermal reaction between acid-treated XC-72R carbon black, an Fe salt, and 1,4-benzenedicarboxylic acid (ligand) followed by pyrolysis under Ar at different temperatures (600–700 °C) in the presence of melamine [116]. The non-calcined intermediate presented well-defined octahedral structures with a uniform size of ≈10 µm, which was mainly ascribed to the limiting effect of carbon black on the growth of larger particles. Temperature strongly influenced the nature of Fe species found in the pyrolyzed catalyst. The XRD pattern of the sample prepared at an optimal temperature of 650 °C revealed the presence of Fe and Fe_3_O_4_ and the absence of Fe_3_C. The high contents of N (5.84 at.%) and Fe (1.18 at.%) revealed by XPS made this material particularly well suited for ORR catalysis, favoring the creation of Fe-N*_x_* sites crucially important for the reduction of O_2_ to water. The sample pyrolyzed at 650 °C exhibited the best ORR activity (onset potential = 0.9 V), which was explained by the abundance of active sites such as Fe_3_O_4_ and Fe-N*_x_* formed at this temperature. The electron transfer number was calculated as ≈3.9 in the potential range of 0.3–0.6 V. Unfortunately, no H_2_O_2_ yield was presented, and methanol tolerance was slightly improved in comparison to that of Pt/C; however, a sharp decrease of current was still observed upon the injection of methanol into the electrolyte. The MOF-based catalyst also demonstrated high stability during chronoamperometric measurements, with 80.8% of initial activity retained after 20,000 s (cf. 73.8%. for Pt/C).

A truly different approach was adopted by Rizvi et al., who prepared a Cu benzene-1,3,5-tricarboxylate MOF (Cu-BTC MOF) in the presence of biomass-derived activated carbon (AC) [117]. Dried and crushed Lantana plant leaves, selected as the starting material for AC synthesis, were activated by treatment with phosphoric acid and calcined at 550 °C. After this step, the growth of Cu-BTC MOF was performed in solution in the presence of AC, and the resulting composite was annealed in Ar/H_2_ at 700 °C to yield Cu@AC. XRD revealed the presence of a partially graphitized carbon matrix as well as metallic Cu, Cu oxide and Cu phosphide (Cu_3_P). Although the specific surface area was not as high as expected (153.3 m^2^ g^−1^), this catalyst showed a significantly improved ORR activity in comparison to AC alone, presenting a surface area two-fold higher and a pore volume (0.284 cm^3^ g^−1^) exceeding that of AC (0.16 cm^3^ g^−1^). This enhancement was ascribed to the presence of multiple Cu-rich species. The above electrocatalyst was mainly studied by CV, while the fact that no RDE/RRDE tests were carried out limits the appreciation of this work. When the electrocatalytic activity of Cu@AC was probed in O_2_-saturated 0.1 M KOH, a reduction peak around 0.9 V was observed. However, this peak was not detected when an N_2_-saturated solution was used. The onset potential of Cu@AC was estimated by CV as 0.8 V at a current density of 2.1 mA cm^−2^, while a value of 0.88 V at 1.37 mA cm^−2^ was obtained for Pt/C. In contrast, negligible currents were observed for raw precursors (AC and Cu-BTC MOF), indicating low ORR activity. Finally, the ORR polarization curves of Cu@AC before and after 2000 cycles between 0 and 1.3 V showed a small negative shift of *E*_1/2_ (≈20 mV), confirming the high stability of this catalyst.

Wen et al. presented another example of a Cu-based MOF, preparing Cu nanoparticles embedded onto N-doped carbon-graphene by the pyrolysis of Cu-MOF/GO precursors [118]. Specifically, 2-methylimidazole was dropwise added to a mixture of GO and CuCl_2_ in methanol. The best catalyst, denoted as Cu@NC-700, was obtained at a GO loading of 5 wt % after 6-h pyrolysis at 700 °C in N_2_. The XRD pattern of Cu@NC-700 showed two distinct peaks at 43.4° and 50.6°, attributed to Cu (111) and Cu (200) lattice planes, respectively. In addition, the related FTIR spectrum featured a characteristic Cu–N peak at 1226 cm^−1^. TEM imaging revealed that Cu nanoparticles in Cu@NC-700 were dispersed on N-doped graphene, with some of them coated by an amorphous carbon layer. EDX mapping demonstrated the uniform dispersion of Cu and N on the N-doped carbonaceous catalyst. The sample was characterized by a high content of Cu, which was determined by ICP as ≈6.3 wt %. Finally, XPS confirmed the presence of Cu^0^ and Cu^2+^ as well as the formation of Cu-N*_x_* active sites. Based on the LSV curves of Cu@NC-700 recorded in 0.1 M KOH, *E*_onset_ and *E*_1/2_ were estimated as 0.926 and 0.86 V, respectively, being slightly lower than the values obtained for Pt/C (*E*_onset_ = 0.95 V). The electron transfer numbers of Cu@NC-700 and Pt/C in the potential range of 0.2–0.6 V were determined as 3.95 and ≈4.0, respectively, suggesting that the H_2_O_2_ yield (not provided by the authors) was low. Catalyst durability was tested by chronoamperometric measurements at a constant potential of 0.7 V for 14 h. Cu@NC-700 maintained 85% of its initial current density, while the commercial Pt/C catalyst retained only 61%, which demonstrated the superior ORR durability of the former catalyst. Finally, Cu@NC-700 presented good methanol tolerance and better CO tolerance than Pt/C.

An Fe/N/S-doped carbon catalyst derived from UIO-66-NH_2_ and featuring a well-inherited octahedral morphology and a well-defined mesoporous structure has recently been prepared by a method featuring double-solvent diffusion pyrolysis [119]. Solvothermally prepared UIO-66-NH_2_ nanocrystals (≈100–200 nm in length) were dispersed in a hydrophobic solvent (hexane), and a hydrophilic solution (H_2_O/methanol) containing NH_4_SCN and FeCl_3_ was dropwise added. Impregnation by stirring followed by isolation, first calcination in Ar, leaching with HF, and second calcination at 900 °C in Ar yielded the Fe/N/S-doped carbon catalyst (denoted as Fe/N/S-PC). HAAD-STEM imaging showed that Fe/N/S-PC had a nano-octahedral morphology with a hierarchical porous structure, which favored the fast diffusion of the electrolyte and O_2_. In addition, no Fe, N, S, or Zr particles were observed; i.e., these elements were well dispersed and incorporated into the carbon framework. The authors stated that the N- and S-containing gases (e.g., NH_3_, HCl, HCN, and HSCN) released during NH_4_SCN pyrolysis had a corrosive effect on the organic ligands, which resulted in an increased average pore size, surface area (1589 m^2^ g^−1^), and mesopore volume (1.361 cm^3^ g^−1^). High-resolution S 2p spectra revealed the presence of thiophene-S bonds (C–S–C), which are known to have a catalytic effect on ORR in addition to Fe-N*_x_* active sites. Thus, Fe/N/S-PC presented good electrochemical performance in alkaline and acid electrolytes. For instance, the onset and half-wave potentials obtained in 0.1 M KOH (0.97 and 0.87 V, respectively) surpassed those of Pt/C (0.94 and 0.84 V, respectively). The average *n* = 3.95 demonstrated a preference for the four-electron reduction pathway. The long-term cyclability of Fe/N/S-PC was evaluated by CV to show that after 5000 cycles, *E*_1/2_ negatively shifted by about 13 and 10 mV in alkaline and acidic electrolytes, respectively, i.e., much less than for Pt/C (30 and 40 mV, respectively). The superior performance of Fe/N/S-PC was attributed to its high surface area, high mesoporosity, and highly active (e.g., graphitic/pyridinic N, Fe-N*x*, and thiophene-S) species.

Nanoporous MOF-derived catalysts were hierarchically prepared by using functional carbon black assembled with MIL-101(Fe) as a precursor followed by nitrogen doping with melamine and carbonization under Ar at 700 °C [120]. First, XC-72R carbon black was activated with sulfuric and nitric acids; then, the MIL-101(Fe) MOF was solvothermally synthesized in the presence of the modified carbon, and the adduct was calcined with melamine as a nitrogen source. The related XRD pattern revealed the presence of Fe_3_C and metallic Fe, and the typical strong peak at 26° corresponded to the (002) facet of graphitic carbon. SEM imaging of Fe/Fe_3_C@NC showed a hybrid structure of functional carbon black and ≈500-nm MOF-derived particles. Carbon black hindered grain aggregation (inevitable in its absence), and particles of up to 10 µm were obtained. TEM imaging revealed the presence of Fe/Fe_3_C nanoparticles (diameter = 10–50 nm) encapsulated in carbon and wrapped in a 5-nm-thick graphitic carbon layer. However, the above catalyst presented a relatively low BET surface area of 107 m^2^ g^−1^. The onset (0.85 V) and half-wave (0.7 V) potentials of Fe/Fe_3_C@NC were similar to those of commercial Pt/C, whereas the former catalyst featured a much higher *J*_L_ than the latter and *n* varying from 3.88 to 4.05 within a 0.3–0.6 V potential window. Tolerance to methanol crossover effects was tested by the addition of 3 M methanol to the electrolyte during a chronoamperometric test. Whereas a sharp current drop was observed for platinum, only a slight decrease was recorded for Fe/Fe_3_C@NC, indicating superior tolerance. Chronoamperometric measurements showed that after 20,000 s, Fe/Fe_3_C@NC retained 85% of the original current density (cf. 73.8% for Pt/C), indicating the superior stability of this catalyst.

A novel Co_2_N_5_ binuclear active site structure was designed and elaborated by Xing et al. [121]. The Co-N-C-10 catalyst was prepared by a simple reaction between 2-methylimidazole (ligand) and a mixture of Zn(NO_3_)_2_·6H_2_O and Co(NO_3_)_2_·6H_2_O salts at a Zn/Co atomic ratio of 10. The catalyst produced upon carbonization retained the initial rhombododecahedral morphology of the MOF precursor. HAADF-STEM imaging revealed the presence of Co nanoparticles and bright atomic-size spots, which were present in two sizes, corresponding to single Co ions and two conjoint Co ions with a Co-Co distance of 2.1–2.2 Å. This finding confirmed the successful fabrication of Co_2_N_5_ binuclear active sites. According to EXAFS analyses, the intrinsic ORR activity of active sites was in the order of Co_2_N_5_ > CoN_4_ > Co@C, as follows from the contribution of these sites to the overall ORR activity. DFT calculations demonstrate than Co_2_N_5_ sites exhibit more than 12-fold higher activity than conventional CoN_4_ active sites, while free energy diagrams demonstrate that OH pre-absorbed on Co_2_N_5_ sites act as modifying ligands to promote the conversion of ^*^OH to H_2_O. Consequently, the above catalyst exhibited superior ORR activity. In an acidic electrolyte, Co-N-C-10 presented an onset potential of 0.92 V and a half-wave potential of 0.79 V that was only 100 mV lower than that of the commercial Pt/C catalyst and much higher than those of most reported MOF-based electrocatalysts. RRDE measurements demonstrated that the peroxide yield was as low as 2% within a wide potential range, with *n* was determined as 3.97. Finally, after 20,000 voltammetric cycles between 0.6 and 1.2 V, the *E*_1/2_ of Co-N-C-10 negatively shifted by 12 mV, i.e., by a value at least two times lower than that of Pt/C.

A three-dimensional S-doped Fe/N/C network with hierarchical micro–meso–macro porosity was prepared by pyrolysis of a mixture of ZIF-8, 2-aminothiazole (2-AT), and FeCl_2_ [122]. The catalyst obtained after calcination presented a porous network consisting of numerous short and curved branches well connected in all three dimensions to form a sponge-like 3D network. Raman spectroscopy (*I*_D_/*I*_G_ = 0.92) suggested that more than half of the carbon existed in a graphitic state, which benefitted electronic conductivity. The material was characterized by abundant uniform mesopores with an average size of 13.3 nm and thus had a high BET specific surface area of 625.3 m^2^ g^−1^. HAADF-STEM imaging and EDX elemental mapping confirmed the homogeneous distribution of Fe, N, S, and C. The importance of S-doping for ORR activity was clearly identified by the authors, and the best S-doped Fe/N/C catalyst featured *E*_1/2_ = 0.820 V and a *J*_L_ of 4.57 mA cm^−2^. The above potential differed from that of commercial Pt/C by only 45 mV, and the *J*_L_ (4.0 mA cm^−2^) of the latter catalyst was lower than that of S-doped Fe/N/C. The average *n* of 3.8 and the low peroxide yield of ≈3.8% were similar to those obtained for Pt/C. Durability experiments were performed at a constant voltage of 0.6 V and 900 rpm, showing that S-doped Fe/N/C retained 87.5% of its initial current density after 40,000 s, while Pt/C lost almost 25.6% after only 20,000 s. Finally, the authors tested their catalyst in a real PEMFC, obtaining a maximal power density of 0.80 W cm^−2^ at 1 bar.

Lv et al. synthesized an ORR catalyst by carbonizing a mixture of ZIF-67 and GO in Ar at 900 °C [123], showing that the pyrolyzed material comprised 2D rGO sheets and ZIF-derived carbon twisted together to form a 3D porous framework. Interestingly, the nitrogen content of the catalyst exceeded that of the control sample prepared without GO. A diffraction peak at 44.2° in the related XRD pattern was indexed to the (111) crystalline facets of Co, suggesting the successful reduction of Co ions to Co^0^ after carbonization. The size of the Co particles was estimated as 10 nm. The above catalyst had a modest BET surface area of 234 m^2^ g^−1^ but featured a nitrogen content of ≈5.36 at% that was suitable for the formation of abundant Co-N*_x_* active sites. XPS revealed the presence of cobalt oxides, e.g., CoO and Co_3_O_4_. The onset and half-wave potentials were obtained by RDE tests in 0.1 M KOH as 1.00 and 0.87 V, respectively, resembling the values recorded for Pt/C (1.02 and 0.87 V, respectively). However, the activity of ZIF-C/rGO in acidic media was still inferior to that of Pt/C, as follows from the related *E*_1/2_ values (0.71 vs. 0.77 V for Pt/C). When the durability of ZIF-C/rGO in an alkaline electrolyte was tested by chronoamperometric measurements, the above catalyst retained about 89% of its initial current density after 7 h, while the Pt/C catalyst presented a current decay of ≈28%. In addition, the former catalyst was more stable than the latter in acidic media.

Similarly to the previous example [124], Gao et al. reported a Co/NC-Gr-A catalyst prepared by the in situ growth of porous ZIF-67 on graphene sheets followed by calcination at 800 °C in a flow of N_2_ [124]. The as-prepared ZIF-67 presented a rhombododecahedral morphology with an average side length of ≈220 nm and particles with holes on their surface. After thermal treatment, the particle shape was inherited, and the graphene sheets were wrapped around the surface of the dodecahedral pyrolyzed MOF particles to enhance electron conductivity. TEM imaging showed that Co/NC-Gr-A comprised Co nanoparticles with a core–shell structure and an average size of 13.4 nm. XRD confirmed the presence of metallic Co, and its crystal size was estimated using Scherrer’s equation as 12 nm, which is a value close to that obtained by TEM. The BET surface area was modest (360.5 m^2^ g^−1^), but the average pore volume (1.14 cm^3^ g^−1^) was fairly high and beneficial for reactant diffusion during the ORR. Finally, the Co 2p core-level spectrum presented two distinct peaks ascribed to metallic Co (778.1 and 793.0 eV) and two peaks attributed to the Co-N*_x_* active sites (780.5 and 796.0 eV; 58% of the total Co). Catalyst performance was assessed in 0.1 M KOH only. The half-wave potential of 0.83 V was slightly lower than that of the benchmark Pt/C catalyst, while the corresponding *J*_L_ values (≈5.6 mA cm^−2^) were similar. In addition, the new catalyst presented a very good methanol tolerance, whereas the polarization curve of Pt/C presented a strong peak at 0.77 V due to methanol oxidation. According to RRDE tests, Co/NC-Gr-A and Pt/C exhibited H_2_O_2_ yields of 11.5 and 5.7%, respectively, with the respective *n* values equaling 3.78 and 3.9. Finally, after 3000 CV cycles between 0.1 and 1.1 V, no variance in *E*_1/2_ was observed, which confirmed the good stability of Co/NC-Gr-A. Unfortunately, no comparison with Pt/C was reported.

**Figure 16:**
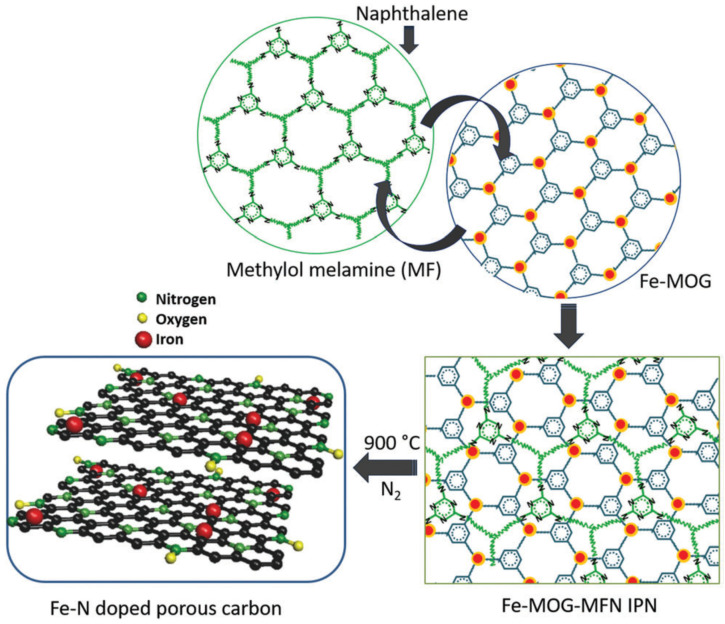
Schematic synthesis of Fe-MOG-MFN-C. Reprinted with permission from ref. [125]. Copyright (2018) Royal Society of Chemistry. MOG: metal–organic gel.

An Fe/N-doped porous graphitic carbon (Fe-MOG-MFN-C) was synthesized by pyrolyzing (in N_2_) an interpenetrating polymer network (IPN) comprising melamine formaldehyde (hard segment) and a metal–organic gel (MOG, soft segment) in the presence of naphthalene (Figure 16) [125]. SEM imaging showed a sheet-like graphitized carbon structure with abundant macropores, while elemental mapping confirmed the homogeneous distribution of N and Fe in the carbon matrix. Fe-MOG-MFN-C exhibited a coherent two-phase morphology and comprised nanoscale star-like structures distributed over graphitic sheets. Moreover, this catalyst featured pores with widths of 2–6 nm and exhibited a high surface area of 950 m^2^ g^−1^. XPS revealed a graphitic nitrogen percentage of ≈1.97% and the existence of an iron oxide phase in the carbon matrix. Electrochemical performance was assessed in 0.1 M KOH, and the onset potential of Fe-MOG-MFN-C (0.91 V) was less than that of Pt/C obtained under the same conditions (1.0 V). The average peroxide yield in the potential range of 0.1–0.6 V equaled 20%, while that of Pt/C equaled 5%. The *n* values were estimated as ≈3.6 and 3.9 for Fe-MOG-MFN-C and Pt/C, respectively. Finally, after an accelerated durability test (5000 CV cycles at 100 mV s^−1^), the *E*_1/2_ of Fe-MOG-MFN-C and Pt/C decreased by 31 and 34 mV, respectively. In conclusion, although the preparation of Fe-MOG-MFN-C was innovative in comparison to most works detailed in this review, the standard Pt/C was superior in terms of ORR catalytic potential while exhibiting similar durability.

In a recent study, ZIF-67 was surface-coated with Co(II) meso-tetra(4-methoxyphenyl)porphine (CoTMPP) to pyrolytically generate an N-doped carbon-containing Co species [126]. Note that ZIF-67 was used as a template for CoTMPP to avoid its aggregation during carbonization at various temperatures. The best-performing sample, denoted as ZIF-67@CoTMPP (800), was obtained at 800 °C. TEM imaging revealed a carbon matrix with numerous well-defined graphitic carbon nanoshells generated by the removal of some Co-based nanoparticles during acid leaching. However, some of these particles were still present, featuring a *d*-spacing of ≈0.20 nm corresponding to the (111) lattice plane of cubic Co. The BET surface area equaled 216 m^2^ g^−1^. XPS suggested that traces of Co were oxidized to Co-based oxides by various oxygen species during the pyrolysis step and confirmed the presence of Co–N bonds. ZIF-67@CoTMPP (800) presented an *E*_1/2_ value that was 9 mV higher than that of Pt/C with a loading of 5 µg_Pt_ cm^−2^ but 59 mV lower than that of Pt/C with a loading of 10 µg_Pt_ cm^−2^. The average *n* of ZIF-67@CoTMPP (800) was determined as 3.36, suggesting that in an alkaline solution, O_2_ was first converted to HO_2_^−^ via two-electron reduction, with a reduction by two further electrons affording OH^−^. In contrast, *n* = 3.98 was obtained for Pt/C, indicating a direct four-electron reduction of O_2_ to H_2_O. Consequently, a high HO_2_^−^ yield of 35.5% was obtained for ZIF-67@CoTMPP (800) (cf. 7.4% for Pt/C). Finally, ZIF-67@CoTMPP (800) presented better durability than commercial Pt/C, as the former *E*_1/2_ value decreased only by 12 mV relative to the initial value of 0.784 V after 5000 cycles, whereas a negative shift of 66 mV was recorded for Pt/C.

Yi et al. reported a self-sacrificial template synthesis of nitrogen-doped hollow carbon microtubes as an ORR electrocatalyst [127]. Polyaniline was deposited on Fe-MIL (Materials of Institut Lavoisier (MOF class)) nanocrystals, and subsequent two-step pyrolysis yielded microstructured carbon tubes with Fe_3_O_4_ nanoparticles encapsulated inside their walls. SEM images of the resulting C-PANI-MIL-SP catalyst showed hollow carbon microtubes with an inner cavity (500 nm in diameter and approximately 3 µm in length) decorated with ≈50-nm metal particles. HR-TEM imaging of these nanoparticles revealed the lattice fringes of Fe_3_O_4_ and showed that the walls of carbon microtubes comprised around 14 graphene layers. XRD confirmed the presence of Fe_3_O_4_ and revealed the absence of metallic Fe particles. The high porosity conferred by hollow carbon microtubes led to a high surface area of 884 m^2^ g^−1^. Electrochemical performance was evaluated in both alkaline and acidic media. In 0.1 M KOH, the ORR catalytic activity of C-PANI-MIL-SP was identical to that of Pt/C (*E*_onset_ = 1.0 V, *E*_1/2_ = 0.87 V). Under acidic conditions, the MOF-based catalyst showed an onset potential (0.86 V) that was slightly lower than that of Pt/C (0.96 V). In addition, in both acidic and alkaline media, C-PANI-MIL-SP showed better durability than Pt/C, featuring current density retentions (82 and 70% in alkaline and acidic media, respectively) superior to those of Pt/C (23 and 28%, respectively).

**Figure 17:**
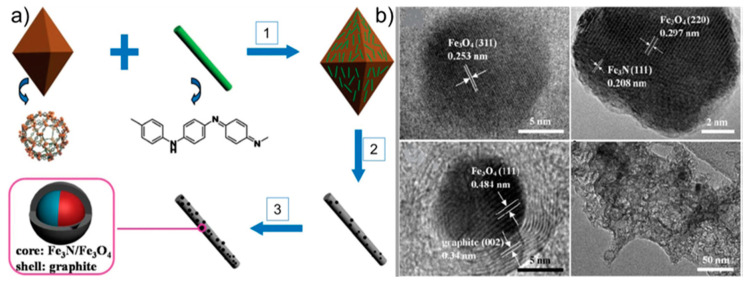
(**a**) Schematic preparation of the C-MIL-101/PANI-8 catalyst obtained by (1) mixing of MIL-101(Fe) MOF with PANI, (2) first heat treatment under N_2_, and (3) acid leaching followed by second heat treatment. (**b**) HR-TEM images of the C-MIL-101/PAni-8 catalyst. Reprinted and modified with permission from ref. [128]. Copyright (2018) Royal Society of Chemistry. MIL: Materials of Institut Lavoisier (MOF class), PANI: polyaniline.

Another MOF-based catalyst prepared using polyaniline (PANI) as a carbon and nitrogen source was reported by Su et al. [128]. First, MIL-101(Fe) nanoparticles were synthesized using a microwave-assisted method and mixed with freshly prepared PANI. Carbonization at 900 °C under N_2_ afforded a catalyst (C-MIL-101/PAni-8) with an optimized core–shell Fe_3_O_4_/Fe_3_N@graphitic carbon structure. As shown in related SEM images, a good dispersion of PANI on octahedral MIL crystals was obtained because of the interaction of the carboxyl groups on the MIL-101(Fe) surface with PANI amino groups. After carbonization, the catalyst exhibited a cross-linked nanorod morphology and contained metallic nanoparticles with an average diameter of 14.8 nm. HR-TEM observations revealed that these particles mainly comprised Fe_3_O_4_ and Fe_3_N, further showing the formation of a core–shell Fe_3_O_4_/Fe_3_N@graphitic carbon structure with an Fe_3_O_4_/Fe_3_N core and a graphitic carbon shell (see HR-TEM images in Figure 17). XRD confirmed the presence of these Fe species. An ideal high BET surface area of 918.4 m^2^ g^−1^ and a high total pore volume of 1.11 cm^3^ g^−1^ were obtained. The deconvoluted N 1s core-level spectrum demonstrated the formation of Fe–N bonds, which possibly originated from Fe_3_N and Fe-N*_x_* active sites. In 0.1 M NaOH, the new catalyst presented a half-wave potential (0.916 V) exceeding that of Pt/C (0.885 V), additionally featuring *n* = 3.96 and a low H_2_O_2_ yield of <3% and thus showing high promise. In addition, after a 100,000-s chronoamperometric test, C-MIL-101/PAni-8 retained 86.2% of its initial current density, while Pt/C presented a rapid current density loss and retained only 46.6% of the initial value.

N-doped porous carbon shells with embedded Fe/Fe_3_C nanoparticles were prepared through the direct pyrolysis of a hexamethylenetetramine (HMT)-impregnated Fe-containing MOF (MIL-100-Fe) [129]. MIL-100-Fe was synthesized via a standard hydrothermal reaction, mixed with various quantities of HMT, and pyrolyzed in N_2_ at different temperatures. The best catalyst (Fe-N_2_-800) was obtained at an HMT/MIL-100-Fe mass ratio of two and a calcination temperature of 800 °C, featuring a BET surface area of 138 m^2^ g^−1^. TEM and SEM analyses revealed the presence of Fe/Fe_3_C nanoparticles coated by carbon layers (≈8 nm thick), as confirmed by XRD and XPS. The electrochemical performances of Fe-N_2_-800 and Pt/C were assessed in an alkaline medium. The onset and half-wave potentials of the MOF-based catalyst equaled −0.077 and −0.25 V (vs. saturated calomel electrode (SCE)), respectively, while those of Pt/C reached −0.033 and −0.175 V, respectively. The HO_2_^−^ yield of Fe-N_2_-800 ranged from 9.2 to 16.8% between −0.8 and −0.2 V (2.5–3.1% for Pt/C) and corresponded to *n* = 3.70–3.91 (cf. 3.92–3.98% for Pt/C). Although Fe-N_2_-800 was obviously inferior to Pt/C, the former catalyst presented better durability. After 5.5 h at a constant potential of −0.6 V, Fe-N_2_-800 lost 3.8% of the initial current density, while Pt/C was not able to retain more than 84.1% of its initial current.

**Table 6:** Recent research works (2018–2020) dealing with the preparation of multi-site electrocatalysts for fuel cells with a focus on ORR activity. Examples in acidic and alkaline media are provided, and major physicochemical properties and electrochemical performances realized using rotating electrodes are compared.

				RDE/RRDE Experiments ***					
Precursors	Main Catalytically Active Sites	BET Surface Area (m^2^ g^−1^)	N/M (N = Nitrogen; M = Metal) Contents (at%) *	*E*_1/2_ (V vs. RHE) **	Average n	Peroxide Yield	Cyclability	Methanol Tolerance	Reference
Co-Hip	Co-N_4_ + Co_2_N + metallic Co	170.26	n/a	0.82 (5 mV s^−1^)	close to 4	n/a	After 2.8 h, 88.2% of initial current at 0.6 V	Excellent under alkaline conditions	[111]
ZIF-8 + MIL-100(Fe)	Fe-N_x_ + Fe-Fe_3_C particles	755	6.47/5.7	0.88 0.5 M H_2_SO_4_: 0.79	≈3.90 0.5 M H_2_SO_4_: ≈3.90	≈1–10% (0.2–0.8 V) 0.5 M H_2_SO_4_: ≈5–8% (0.2–0.8 V)	After 5.6 h, 92.2% of initial current at 0.55 V	Excellent under alkaline conditions	[112]
5,6-Disubstituted 1H-benzo[d]imidazoles + FeCl_3_	Fe-N_x_ + Fe-Fe_3_C particles	380	0.6/0.9 (wt %)	0.87	close to 4	n/a	n/a	n/a	[113]
IRMOF-3 + melamine + SDBS + Fe(NO_3_)_3_	Fe-N_x_ + Fe-Fe_3_C particles + N/S co-doped carbon	267	6.5/1.4	0.1 M NaOH: 0.89 (900 rpm)	0.1 M NaOH: 3.9 (0.4 V)	n/a	0.1 M NaOH: after 2.8 h, 94.3% of initial current at 0.8 V	Excellent under alkaline conditions	[26]
ZIF-8 + FeSO_4_ + hydrazine	Fe-N_x_ + C-S-C	974	9.65(N) + 0.98(S)/1.6	0.91 0.5 M H_2_SO_4_: 0.78	≈3.95 0.5 M H_2_SO_4_: ≈4.0	1.8–5% (0.2–0.8 V) 0.5 M H_2_SO_4_: <1% (0.2–0.9 V)	After 12 h, 94.79% of initial current 0.5 M H_2_SO_4_: after 16 h, 88.54% of initial current	Excellent under alkaline and acidic conditions	[114]
4-py-TTF-4-py + PTA + MWCNTs	Co-N_x_ + C-S-C	n/a	n/a/2.6 (wt %)	0.782	3.82–3.97	5–8% (0.3–0.7 V)	After 2.8 h, 96.5% of initial current at 0.692 V	Excellent under alkaline conditions	[115]
MIL-101(Fe) + XC-72R + melamine	Fe-N_x_ + Fe_3_O_4_	104	5.84/1.18	0.9 (onset potential)	≈3.9 (0.3–0.6 V)	n/a	After 5.6 h, 80.8% of initial current at 0.7 V	Medium under alkaline conditions	[116]
H_3_BTC + Cu(NO_3_)_2_ + AC from biomass	Cu-rich particles	153.3	None/6.87	This catalyst was mainly studied by CV					[117]
CuCl_2_ + 2-mIm + GO	Cu nanoparticles + Cu-N_x_	n/a	n/a/6.3 (wt %)	0.86 (5 mV s^−1^)	3.95 (0.2–0.6 V)	n/a	After 14 h, 85% of initial current at 0.7 V	Excellent under alkaline conditions	[118]
UIO-66-NH_2_ + NH_4_SCN + FeCl_3_	Fe-N_x_ + C-S-C	1589	4.93(N) + 1.49(S)/0.31	0.87 0.1 M HClO_4_: 0.785	≈3.95 (0.5–0.7 V) 0.1 M HClO_4_: ≈3.6 (0.5–0.7 V)	n/a	Stability experiments realized by CV (50 mV s^−1^, 0.6–1.0 V, 5000 cycles): negative *E*_1/2_ shift of 13 mV 0.1 M HClO_4_: negative *E*_1/2_ shift of 10 mV	n/a	[119]
MIL-101(Fe) + modified XC-72R + melamine	Fe-N_x_ + Fe-Fe_3_C nanoparticles	107	5.4/n/a	0.70	≈3.88–4.05 (0.3–0.6 V)	n/a	After 5.6 h, 85% of initial current	Medium under alkaline conditions	[120]
ZIF-67 + ZIF-8	Co nanoparticles + CoN_4_ + Co_2_N_5_	850	5.17/1.06	0.1 M HClO_4_: 0.79 (5 mV s^−1^)	0.1 M HClO_4_: ≈3.97	0.1 M HClO_4_: <2% (0.1–0.8 V)	Stability experiments realized by CV (100 mV s^−1^, 0.6–1.2 V, 20000 cycles): 0.1 M HClO_4_: negative *E*_1/2_ shift of 12 mV	n/a	[121]
ZIF-8 + 2-AT + FeCl_2_	Fe-N_x_ + C-S-C	625.3	2.70 (wt %) (N) + 1.64 (wt %) (S)/0.13	0.1 M H_2_SO_4_: 0.82 (900 rpm)	0.1 M H_2_SO_4_: ≈3.8	0.1 M H_2_SO_4_: 3.8% (0.2–0.8 V)	0.1 M H_2_SO_4_: after 11.1 h, 87.5% of initial current at 0.6 V, 900 rpm	n/a	[122]
ZIF-67 + GO	Co-N_x_ + CoO + Co_3_O_4_	234.3	5.36/0.76	0.87 (5 mV s^−1^) 0.5 M H_2_SO_4_: 0.71 (5 mV s^−1^)	3.82 (0.3–0.5 V)	n/a	After 7 h, 89% of initial current at −0.13 V 0.5 M H_2_SO_4_: after 7 h, 61% of initial current at 0.71 V	n/a	[123]
ZIF-67 + GO	Co nanoparticles + Co-N_x_	360.5	7.06/2.01	0.83	3.78	11.5%	Stability experiments realized by CV (50 mV s^−1^, 0.1–1.1 V, 3000 cycles): quasi no variance in *E*_1/2_	Excellent under alkaline conditions	[124]
H_3_BTC + FeCl_3_ + melamine + naphthalene	Fe-N_x_ + iron oxide	950	1.97(graphitic N)/0.15	0.91 (onset potential, 5 mV s^−1^)	≈3.6	≈20% (0.1–0.6 V)	Stability experiments realized by CV (100 mV s^−1^, 0.57–0.97 V, 5000 cycles): negative *E*_1/2_ shift of 31 mV	n/a	[125]
ZIF-67 + CoTMPP	Co-N_x_ + Co nanoparticles + Co-based oxides	216	n/a	0.784 (5 mV s^−1^)	3.36	<35.5% (0.0–0.8 V)	Stability experiments realized by CV (5000 cycles): negative *E*_1/2_ shift of 12 mV	Medium under alkaline conditions	[126]
MIL-101(Fe) + PANI	FeN_2+2_/ FeN_4_ + Fe_3_O_4_	884	2.21/0.13	0.870.1 M HClO_4_: 0.67	≈3.7 0.1 M HClO_4_: ≈3.5	<4% (0.5–0.8 V) 0.1 M HClO_4_: ≈15% (0.2–0.5 V)	Stability experiments realized by CV (100 mV s^−1^, 0.6–1.0 V): (10,000 cycles): 82% of initial current 0.1 M HClO_4_ (5000 cycles): 70% of initial current	Excellent under alkaline and acidic conditions	[127]
MIL-101(Fe) + PANI	Fe-N_x_ + Fe_3_N + Fe_3_O_4_	918.4	1.4/0.1	0.1 M NaOH: 0.916 (900 rpm)	0.1 M NaOH: ≈3.96	0.1 M NaOH: <3% (0.4–0.9 V)	0.1 M NaOH: after 27.8 h, 86.2% of initial current at 0.8 V	Excellent under alkaline conditions	[128]
MIL-101(Fe) + HMT	Fe-N_x_ + Fe/Fe_3_C nanoparticles	138	3.13/2.99	−0.25 vs. SCE	3.70–3.91	9.2–16.8% (−0.8 to −0.2 V vs. SCE)	After 5.5 h, 96.2% of initial current at −0.6 V vs. SCE	Excellent under alkaline conditions	[129]

* Sometimes, weight percentage was used, and hence, “wt %” data are provided. More than one metal or heteroatom can be provided depending on the active sites involved. ** Potentials are referenced to RHE except when stated otherwise. The ORR performance values were evaluated at 1600 rpm and 10 mV s^−1^ except when stated otherwise. *** All the ORR performance values listed were measured in 0.1 M KOH except when stated otherwise.

## 4. Conclusion and Perspectives

**Figure 18:**
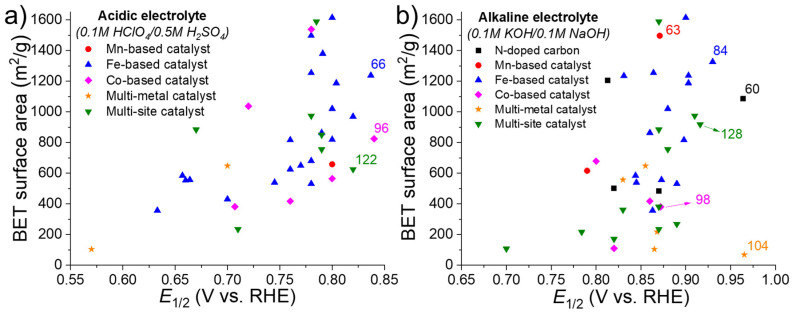
Graphic of Brunauer-Emmet-Teller (BET) surface area vs. *E*_1/2_ for the different categories of MOF-based electrocatalysts investigated in (**a**) acidic and (**b**) alkaline media. The best electrocatalysts in terms of *E*_1/2_ for each category are identified with the corresponding reference number. Note that some works are not represented because they do not report BET surface area, use an electrolyte that is too exotic to be compared with other works (e.g., phosphate buffer solution), or *E*_1/2_ is reported vs. another reference.

Figure 18 lists the BET surface areas and half-wave potentials of the electrocatalysts discussed herein, with some works not included because of missing information. Figure 18a relates to catalysts characterized in acidic media, while Figure 18b relates to catalysts characterized in alkaline media. As the onset/half-wave potentials depend on electrolyte pH [130], the *E*_1/2_ values in alkaline media are slightly higher than those recorded in acidic media. Given that only a few examples were presented for certain catalyst categories (i.e., Mn-based catalysts, N-doped carbons, multi-metal catalysts), it was fairly hard to determine the best one. The above graphics also contain references to works reporting the highest *E*_1/2_ value for each category. The benchmark Pt/C catalyst is not presented, as its *E*_1/2_ value is strongly impacted by the method of catalyst ink fabrication and electrochemical parameters, which vary from one publication to another. However, it can be stated that ORR performance (represented by *E*_1/2_) is positively correlated with BET surface area, except for some examples such as the catalyst reported by Fang et al. [104]. In this case, the catalyst presented a BET surface area of only 68.09 m^2^ g^−1^ but featured the highest half-wave potential in its category (multi-metal catalysts). However, as indicated below, other crucial factors strongly impact the ORR performance.

For N-doped carbons, the best ORR performance was observed for the catalyst with an interconnected carbon structure presented by Zhao et al. [60] (Figure 3). In this example, the macropores generated by the removal of the NaCl template contributed to fast mass transfer, and the high ORR activity was ascribed to the large BET surface area (1086 m^2^ g^−1^) and total pore volume (2.0 cm^3^ g^−1^). When tested under the same conditions in an alkaline medium, the benchmark Pt/C catalyst presented a half-wave potential of 0.834 V, while the N-doped carbon gave a value of 0.862 V.

Recent examples of Mn-based catalysts are scarce, with the best-performing catalyst in this category being a hybrid Mn*_x_*O*_y_*/N-C material introduced by Ahmad Shah et al. and prepared via a PSE method [63] (Figure 5). The employed method is interesting and allows one to replace a fraction of the inactive Zn metal by Mn^2+^ ions without affecting the MOF structure. This electrocatalyst presented one of the highest BET surface areas among the materials investigated in this review (1497 m^2^ g^−1^), which is probably one of the reasons for its high ORR activity. In fact, the material presented a relatively high half-wave potential of 0.871 V, which exceeded that of the standard Pt/C under the same conditions (0.854 V).

Among Fe-based catalysts, the highest *E*_1/2_ value in an acidic electrolyte was reported by Liu et al. [66]. The high performance was attributed to a well-graphitized structure that ensured good electrical conductivity for electron transfer, while the high specific surface area offered abundant space to uniformly distribute the atomically isolated Fe active sites and increase the area of contact with the electrolyte. In an alkaline electrolyte, the SA-Fe-NHPC catalyst synthesized by Chen et al. presented the best performance [84]. This catalyst showed higher ORR activity than the benchmark Pt/C catalyst tested under the same conditions, presenting a half-wave potential of 0.93 V (0.85 V for Pt/C). The combination of high surface area, high pore volume (2.7 cm^3^ g^−1^), hierarchically porous carbon structure, and well-dispersed Fe-N*_x_* active sites contributed to the enhanced electrochemical performance, according to the authors.

For the Co-based catalyst category, the surfactant-assisted MOF approach (Figure 9) employed by Wu et al. [96]. led to a catalyst with an impressive ORR activity in acidic media (*E*_1/2_ = 0.84 V). The above value is the highest reported in this review for a catalyst tested in an acid medium, and the authors claimed that this catalyst outperforms previously reported PGM- and Fe-free catalysts, setting a new record. In this example, the confinement effect of the surfactant yielded a core–shell structure with atomically dispersed Co-N-C sites and a high density of active sites. In addition, this catalyst presented the highest *I*_D_/*I*_G_ value (≈1.52) among all the materials investigated, which is crucial to ensure fast electron transfer. In alkaline media (Figure 11), the mesoporous carbon microspheres prepared by Bai et al. [98] showed the best ORR activity in the Co-based catalyst category. Although the BET surface area was not very high (381 m^2^ g^−^), this catalyst had a high pore volume of 1.25 cm^3^ g^−1^ and a low micropore surface area (14.8 m^2^ g^−1^), thus being a typical mesoporous material. This feature is crucial to increase the exposure of catalytic sites to the electrolyte as well as facilitate mass transport.

Concerning multi-site catalysts, the best catalyst tested in an acidic electrolyte was the sponge-like 3D network material reported by Wu et al. [122]. This catalyst presented hierarchical micro–meso–macro porosity with abundant uniform mesopores and a high BET specific surface area of 625.3 m^2^ g^−1^. In addition to the homogeneous distribution of Fe, N, S, and C observed for this sample, the above features are mandatory to achieve good ORR activity. The catalyst featured a half-wave potential (0.820 V) similar to that of Pt/C (0.865 V) but presented a higher limiting current density, which was probably due to the enhanced diffusion of species in the hierarchical pore structure. The MOF-based catalyst proposed by Su et al. [128] showed the best performance in alkaline media, featuring a half-wave potential (0.916 V) that was above that of Pt/C (0.885 V). The material was characterized by a high BET surface area (918.4 m^2^ g^−1^) and high porosity due to a cross-linked carbonaceous structure. The core–shell Fe_3_O_4_/Fe_3_N@graphitic carbon structure (Figure 17) favored fast electron/active species transfer that is mandatory to achieve high ORR activity.

Finally, in most works dealing with the preparation of MOF-based electrocatalysts, Zn atoms were used to form highly structured metal–organic frameworks via the evaporation of this metal during calcination to create porosity. Al-Zoubi et al. presented an innovative way of synthesizing MOFs using Cd instead of Zn [79]. Cd, defined as a low-temperature sacrificial metal by the authors, helped preserve the single-atom Fe active sites and limited their agglomeration into metal nanoparticles, which would be detrimental for ORR activity. The proposed method seems to provide highly dispersed metal-N sites but is probably not applicable on an industrial scale because of the high price of Cd.

Taking into account the works presented in this review, the following crucial criteria have to be taken into account or respected to achieve high ORR activity:

1. A hierarchical porous structure and a high pore volume to facilitate gas/liquid diffusion.
2. A high specific surface area to increase the contact between the active sites and the electrolyte.
3. A highly graphitized carbonaceous structure (high *I*_D_/*I*_G_) to ensure efficient electron delocalization.
4. Atomically dispersed metal-N active sites to increase ORR activity.
5. High metal/nitrogen content of the carbonaceous catalyst.
6. To be accessible by the electrolyte/electrons, metal quantum dots/active sites should be dispersed on the catalyst surface and not be present in the bulk of the material (i.e., not be electrically isolated).
7. Metal agglomeration has to be avoided during calcination.

## List of Abbreviations and Acronyms

2-AT = 2-Aminothiazole	Hip = 1H-imidazo[4,5-f][1,10]phenanthroline	PANI = Polyaniline
2-mIm = 2-Methylimidazole	HMT = Hexamethylenetetramine	PBS = Phosphate buffer solution
4-py-TTF-4-py = 4-Pyridyl-tetrathiafulvalene-4-pyridyl	HNCSs = Hollow N-doped carbon microspheres	PEMFCs = Proton-exchange membrane fuel cells
AC = Activated carbon	HR-TEM = High-resolution TEM	PGM = Pt-group metal
BET = Brunauer–Emmet–-Teller	ICP = Inductively coupled plasma	PTA = Terephthalic acid
BIM = Benzimidazole	IPN = Interpenetrating polymer network	PVP = Polyvinylpyrrolidone
[bmim][Tf2N] = 1-Butyl-3-methylimidazoliumbis(trifiuoromethylsulfonyl) imide	IRES = Intermittent renewable energy sources	RDE = Rotating disk electrode
CNT = Carbon nanotube	IRMOF = Isoreticular metal organic framework	RHE = Reversible hydrogen electrode
CV = Cyclic voltammetry	*J*_L_ = Diffusion-limited current density	rpm = Rotation per minute
CVD = Chemical vapor deposition	K-L = Koutecky–Levich	RRDE = Rotating ring-disk electrode
DAP = 2,6-diaminopyridine	LSV = Linear sweep voltammetry	SAED = Selected area electron diffraction
DFT = Density functional theory	MAC = Metal–ammonia complex	SCE = Saturated calomel electrode
DOE = Department of Energy	MEA = Membrane electrode assembly	SEM = Scanning electron microscopy
*E*_1/2_ = Half-wave potential	MIL = Materials of Institut Lavoisier (MOF class)	STEM = Scanning transmission electron microscopy
EDS/EDX = Energy-dispersive spectroscopy	MOF = Metal–organic frameworks	TCPP = Tetrakis(4-carboxyphenyl)porphyrin
*E*_onset_ = Onset potential	MOG = Metal–organic gel	TEM = Transmission electron microscopy
EXAFS = Fourier transform extended X-ray absorption fine structure	MWCNTs = Multi-walled carbon nanotubes	TEOS = Tetraethylorthosilicate
FeAc = Ferrous acetate	n = Electron transfer number	UIO = University of Oslo (MOF class)
Fe(acac)3 = Iron(III) 2,4-pentanedionate	n/a = Not applicable	XC-72R = Type of carbon black
FePc = Iron(II) phthalocyanine	NTCDA = 1,4,5,8-Naphthalenetetracarboxylic anhydride	XPS = X-ray photoelectron spectroscopy
GO = Graphene oxide	OER = Oxygen evolution reaction	XRD = X-ray diffraction
H_3_BTC = 1,3,5-Benzenetricarboxylic acid	ORR = Oxygen reduction reaction	ZIF = Zeolitic imidazolate framework
HAADF-STEM = High-angle annular dark-field STEM	P123 = Triblock copolymers poly(ethylene oxide)-b-poly(propylene oxide)-b-poly(ethylene oxide)	

## References

[1] Engels A., Kunkis M., Altstaedt S. (2020). A New Energy World in the Making: Imaginary Business Futures in a Dramatically Changing World of Decarbonized Energy Production. Energy Res. Soc. Sci..

[2] Quaschning V. (2019). Front Matter. Renewable Energy and Climate Change.

[3] Lin B., Zhu J. (2019). Determinants of Renewable Energy Technological Innovation in China under CO_2_ Emissions Constraint. J. Environ. Manag..

[4] Zeng J., Liu T., Feiock R., Li F. (2019). The Impacts of China’s Provincial Energy Policies on Major Air Pollutants: A Spatial Econometric Analysis. Energy Policy.

[5] World Energy Needs and Nuclear Power|Energy Needs|Nuclear Energy meeting Energy Needs—World Nuclear Association. https://www.world-nuclear.org/information-library/current-and-future-generation/world-energy-needs-and-nuclear-power.aspx.

[6] Ho S.S., Kristiansen S. (2019). Environmental Debates over Nuclear Energy: Media, Communication, and the Public. Environ. Commun..

[7] Al-Ghussain L., Samu R., Taylan O., Fahrioglu M. (2020). Sizing Renewable Energy Systems with Energy Storage Systems in Microgrids for Maximum Cost-Efficient Utilization of Renewable Energy Resources. Sustain. Cities Soc..

[8] Dua R., White K., Lindland R. (2019). Understanding Potential for Battery Electric Vehicle Adoption Using Large-Scale Consumer Profile Data. Energy Rep..

[9] Rivard E., Trudeau M., Zaghib K. (2019). Hydrogen Storage for Mobility: A Review. Materials.

[10] MIT Study: Quebec Hydro Reservoirs Provide Valuable Energy Storage for U.S. Northeast. https://www.hydroreview.com/2020/02/18/mit-study-quebec-hydro-reservoirs-provide-valuable-energy-storage-for-u-s-northeast/.

[11] Thema M., Bauer F., Sterner M. (2019). Power-to-Gas: Electrolysis and Methanation Status Review. Renew. Sustain. Energy Rev..

[12] Abe J.O., Popoola A.P.I., Ajenifuja E., Popoola O.M. (2019). Hydrogen Energy, Economy and Storage: Review and Recommendation. Int. J. Hydrogen Energy.

[13] Ren X., Dong L., Xu D., Hu B. (2020). Challenges towards Hydrogen Economy in China. Int. J. Hydrogen Energy.

[14] Sinigaglia T., Freitag T.E., Kreimeier F., Martins M.E.S. (2019). Use of Patents as a Tool to Map the Technological Development Involving the Hydrogen Economy. World Pat. Inf..

[15] Ditaranto M., Heggset T., Berstad D. (2020). Concept of Hydrogen Fired Gas Turbine Cycle with Exhaust Gas Recirculation: Assessment of Process Performance. Energy.

[16] Ghorbani B., Vijayaraghavan K. (2019). A Review Study on Software-Based Modeling of Hydrogen-Fueled Solid Oxide Fuel Cells. Int. J. Hydrogen Energy.

[17] Widera B. (2020). Renewable Hydrogen Implementations for Combined Energy Storage, Transportation and Stationary Applications. Therm. Sci. Eng. Prog..

[18] Satyapal S. Hydrogen and Fuel Cell Program Overview. https://www.hydrogen.energy.gov/pdfs/review19/plenary_overview_satyapal_2019.pdf.

[19] (2020). Fuel Cell Market Size & Share|Industry Growth Report. https://www.grandviewresearch.com/industry-analysis/fuel-cell-market.

[20] Prokop M., Drakselova M., Bouzek K. (2020). Review of the Experimental Study and Prediction of Pt-Based Catalyst Degradation during PEM Fuel Cell Operation. Curr. Opin. Electrochem..

[21] Wang Y., Ruiz Diaz D.F., Chen K.S., Wang Z., Adroher X.C. (2020). Materials, Technological Status, and Fundamentals of PEM Fuel Cells—A Review. Mater. Today.

[22] Wilson A., Kleen G., Papageorgopoulos D. DOE Hydrogen and Fuel Cells Program Record. https://www.hydrogen.energy.gov/pdfs/17007_fuel_cell_system_cost_2017.pdf.

[23] Arnold R. The Lowdown on Hydrogen—Part 1: Transportation. https://energypost.eu/the-lowdown-on-hydrogen-part-1-transportation/v.

[24] Dombrovskis J.K., Palmqvist A.E.C. (2016). Recent Progress in Synthesis, Characterization and Evaluation of Non-Precious Metal Catalysts for the Oxygen Reduction Reaction. Fuel Cells.

[25] ElectroCat Consortium. https://www.electrocat.org/.

[26] Zhou S., Zhou Q.-X., Su H., Wang Y., Dong Z., Dai X., Zhang X. (2019). Hybrid of Fe3C@N, S Co-Doped Carbon Nanotubes Coated Porous Carbon Derived from Metal Organic Frameworks as an Efficient Catalyst towards Oxygen Reduction. J. Colloid Interface Sci..

[27] Jaouen F., Proietti E., Lefèvre M., Chenitz R., Dodelet J.-P., Wu G., Chung H.T., Johnston C.M., Zelenay P. (2010). Recent Advances in Non-Precious Metal Catalysis for Oxygen-Reduction Reaction in Polymer Electrolyte Fuel Cells. Energy Environ. Sci..

[28] Huang X., Shen T., Zhang T., Qiu H., Gu X., Ali Z., Hou Y. (2020). Efficient Oxygen Reduction Catalysts of Porous Carbon Nanostructures Decorated with Transition Metal Species. Adv. Energy Mater..

[29] Zhang K., Liang Z., Zou R. (2020). Metal-Organic Frameworks for Electrochemical Energy Conversion: Status and Challenges. Sci. China Chem..

[30] He Y., Tan Q., Lu L., Sokolowski J., Wu G. (2019). Metal-Nitrogen-Carbon Catalysts for Oxygen Reduction in PEM Fuel Cells: Self-Template Synthesis Approach to Enhancing Catalytic Activity and Stability. Electrochem. Energy Rev..

[31] Thanasilp S., Hunsom M. (2010). Effect of MEA Fabrication Techniques on the Cell Performance of Pt–Pd/C Electrocatalyst for Oxygen Reduction in PEM Fuel Cell. Fuel.

[32] Li G., Anderson L., Chen Y., Pan M., Chuang P.-Y.A. (2017). New Insights into Evaluating Catalyst Activity and Stability for Oxygen Evolution Reactions in Alkaline Media. Sustain. Energy Fuels.

[33] Xia W., Mahmood A., Liang Z., Zou R., Guo S. (2016). Earth-Abundant Nanomaterials for Oxygen Reduction. Angew. Chem. Int. Ed..

[34] Yang H.B., Miao J., Hung S.-F., Chen J., Tao H.B., Wang X., Zhang L., Chen R., Gao J., Chen H.M. (2016). Identification of Catalytic Sites for Oxygen Reduction and Oxygen Evolution in N-Doped Graphene Materials: Development of Highly Efficient Metal-Free Bifunctional Electrocatalyst. Sci. Adv..

[35] Li D., Xu H.-Q., Jiao L., Jiang H.-L. (2019). Metal-Organic Frameworks for Catalysis: State of the Art, Challenges, and Opportunities. EnergyChem.

[36] Liu B., Shioyama H., Akita T., Xu Q. (2008). Metal-Organic Framework as a Template for Porous Carbon Synthesis. J. Am. Chem. Soc..

[37] Dang S., Zhu Q.-L., Xu Q. (2018). Nanomaterials Derived from Metal–Organic Frameworks. Nat. Rev. Mater..

[38] Wang C., Yang H., Zhang Y., Wang Q. (2019). NiFe Alloy Nanoparticles with Hcp Crystal Structure Stimulate Superior Oxygen Evolution Reaction Electrocatalytic Activity. Angew. Chem. Int. Ed..

[39] Wang H.-F., Chen L., Pang H., Kaskel S., Xu Q. (2020). MOF-Derived Electrocatalysts for Oxygen Reduction, Oxygen Evolution and Hydrogen Evolution Reactions. Chem. Soc. Rev..

[40] Zhu Q.-L., Xia W., Akita T., Zou R., Xu Q. (2016). Metal-Organic Framework-Derived Honeycomb-Like Open Porous Nanostructures as Precious-Metal-Free Catalysts for Highly Efficient Oxygen Electroreduction. Adv. Mater..

[41] Qian Y., Hu Z., Ge X., Yang S., Peng Y., Kang Z., Liu Z., Lee J.Y., Zhao D. (2017). A Metal-Free ORR/OER Bifunctional Electrocatalyst Derived from Metal-Organic Frameworks for Rechargeable Zn-Air Batteries. Carbon.

[42] Jiang S., Sun Y., Dai H., Hu J., Ni P., Wang Y., Li Z., Li Z. (2015). Nitrogen and Fluorine Dual-Doped Mesoporous Graphene: A High-Performance Metal-Free ORR Electrocatalyst with a Super-Low HO2− Yield. Nanoscale.

[43] Li M., Wang C., Hu S., Wu H., Feng C., Zhang Y. (2019). Nitrogen, Phosphorus Co-Doped Mesoporous Carbon Materials as Efficient Catalysts for Oxygen Reduction Reaction. Ionics.

[44] Yin P., Yao T., Wu Y., Zheng L., Lin Y., Liu W., Ju H., Zhu J., Hong X., Deng Z. (2016). Single Cobalt Atoms with Precise N-Coordination as Superior Oxygen Reduction Reaction Catalysts. Angew. Chem. Int. Ed..

[45] Chen Y., Ji S., Zhao S., Chen W., Dong J., Cheong W.-C., Shen R., Wen X., Zheng L., Rykov A.I. (2018). Enhanced Oxygen Reduction with Single-Atomic-Site Iron Catalysts for a Zinc-Air Battery and Hydrogen-Air Fuel Cell. Nat. Commun..

[46] Xiao M., Zhu J., Ma L., Jin Z., Ge J., Deng X., Hou Y., He Q., Li J., Jia Q. (2018). Microporous Framework Induced Synthesis of Single-Atom Dispersed Fe-N-C Acidic ORR Catalyst and Its in Situ Reduced Fe-N_4_ Active Site Identification Revealed by X-Ray Absorption Spectroscopy. ACS Catal..

[47] Wang X.X., Cullen D.A., Pan Y.-T., Hwang S., Wang M., Feng Z., Wang J., Engelhard M.H., Zhang H., He Y. (2018). Nitrogen-Coordinated Single Cobalt Atom Catalysts for Oxygen Reduction in Proton Exchange Membrane Fuel Cells. Adv. Mater. Deerfield Beach Fla.

[48] Masa J., Zhao A., Xia W., Muhler M., Schuhmann W. (2014). Metal-Free Catalysts for Oxygen Reduction in Alkaline Electrolytes: Influence of the Presence of Co, Fe, Mn and Ni Inclusions. Electrochim. Acta.

[49] Chen Y., Ji S., Wang Y., Dong J., Chen W., Li Z., Shen R., Zheng L., Zhuang Z., Wang D. (2017). Innenrücktitelbild: Isolated Single Iron Atoms Anchored on N-Doped Porous Carbon as an Efficient Electrocatalyst for the Oxygen Reduction Reaction (Angew. Chem. 24/2017). Angew. Chem..

[50] Paul R., Zhu L., Chen H., Qu J., Dai L. (2019). Recent Advances in Carbon-Based Metal-Free Electrocatalysts. Adv. Mater..

[51] Wang J., Wu G., Wang W., Xuan W., Jiang J., Wang J., Li L., Lin W.-F., Ding W., Wei Z. (2019). A Neural-Network-like Catalyst Structure for the Oxygen Reduction Reaction: Carbon Nanotube Bridged Hollow PtCo Alloy Nanoparticles in a MOF-like Matrix for Energy Technologies. J. Mater. Chem. A.

[52] Xue Y., Li H., Ye X., Yang S., Zheng Z., Han X., Zhang X., Chen L., Xie Z., Kuang Q. (2019). N-Doped Carbon Shell Encapsulated PtZn Intermetallic Nanoparticles as Highly Efficient Catalysts for Fuel Cells. Nano Res..

[53] Nadeem M., Yasin G., Arif M., Bhatti M.H., Sayin K., Mehmood M., Yunus U., Mehboob S., Ahmed I., Flörke U. (2020). Pt-Ni@PC900 Hybrid Derived from Layered-Structure Cd-MOF for Fuel Cell ORR Activity. ACS Omega.

[54] Hanif S., Shi X., Iqbal N., Noor T., Anwar R., Kannan A.M. (2019). ZIF Derived PtNiCo/NC Cathode Catalyst for Proton Exchange Membrane Fuel Cell. Appl. Catal. B Environ..

[55] Xue S., Yu Y., Wei S., Xu B., Lei J., Ban R., Wu Q., Zheng M., Li J., Kang J. (2018). Nitrogen-Doped Porous Carbon Derived from ZIF-8 as a Support of Electrocatalyst for Enhanced Oxygen Reduction Reaction in Acidic Solution. J. Taiwan Inst. Chem. Eng..

[56] Chai L., Zhang L., Wang X., Xu L., Han C., Li T.-T., Hu Y., Qian J., Huang S. (2019). Bottom-up Synthesis of MOF-Derived Hollow N-Doped Carbon Materials for Enhanced ORR Performance. Carbon.

[57] Huang G., Ren M., Wang Y., Zhou J., Cai J. (2019). Direct Carbonization of ZIF-8 to N-Doped Carbons: Amino Acid Modulation and Enhanced Catalytic Activity for Oxygen Reduction Reaction. Mater. Chem. Phys..

[58] Li Z., Gao Q., Qian W., Tian W., Zhang H., Zhang Q., Liu Z. (2018). Ultrahigh Oxygen Reduction Reaction Electrocatalytic Activity and Stability over Hierarchical Nanoporous N-Doped Carbon. Sci. Rep..

[59] Tong J., Li W., Bo L., Ma J., Li T., Li Y., Zhang Q., Fan H. (2018). Composite of Hierarchically Porous N-Doped Carbon/Carbon Nanotube with Greatly Improved Catalytic Performance for Oxygen Reduction Reaction. ACS Sustain. Chem. Eng..

[60] Qian Y., An T., Birgersson K.E., Liu Z., Zhao D. (2018). Web-Like Interconnected Carbon Networks from NaCl-Assisted Pyrolysis of ZIF-8 for Highly Efficient Oxygen Reduction Catalysis. Small Weinh. Bergstr. Ger..

[61] Singh H., Zhuang S., Nunna B.B., Lee E.S. (2018). Thermal Stability and Potential Cycling Durability of Nitrogen-Doped Graphene Modified by Metal-Organic Framework for Oxygen Reduction Reactions. Catalysts.

[62] Asadian E., Shahrokhian S., Iraji Zad A. (2020). ZIF-8/PEDOT @ Flexible Carbon Cloth Electrode as Highly Efficient Electrocatalyst for Oxygen Reduction Reaction. Int. J. Hydrogen Energy.

[63] Ahmad Shah S.S., Najam T., Cheng C., Chen S., Xiang R., Peng L., Lu L., Ding W., Wei Z. (2018). Design and Synthesis of Conductive Carbon Polyhedrons Enriched with Mn-Oxide Active-Centres for Oxygen Reduction Reaction. Electrochim. Acta.

[64] Gonen S., Lori O., Cohen-Taguri G., Elbaz L. (2018). Metal Organic Frameworks as a Catalyst for Oxygen Reduction: An Unexpected Outcome of a Highly Active Mn-MOF-Based Catalyst Incorporated in Activated Carbon. Nanoscale.

[65] Li J., Chen M., Cullen D.A., Hwang S., Wang M., Li B., Liu K., Karakalos S., Lucero M., Zhang H. (2018). Atomically Dispersed Manganese Catalysts for Oxygen Reduction in Proton-Exchange Membrane Fuel Cells. Nat. Catal..

[66] Liu D., Li J., Ding S., Lyu Z., Feng S., Tian H., Huyan C., Xu M., Li T., Du D. (2020). 2D Single-Atom Catalyst with Optimized Iron Sites Produced by Thermal Melting of Metal–Organic Frameworks for Oxygen Reduction Reaction. Small Methods.

[67] Wang Y., Wang J., Wei D., Li M. (2019). A “MOF-Protective-Pyrolysis” Strategy for the Preparation of Fe–N–C Catalysts and the Role of Fe, N, and C in the Oxygen Reduction Reaction in Acidic Medium. ACS Appl. Mater. Interfaces.

[68] Zhang X., Huang X., Hu W., Huang Y. (2019). A Metal-Organic Framework-Derived Fe–N–C Electrocatalyst with Highly Dispersed Fe–Nx towards Oxygen Reduction Reaction. Int. J. Hydrogen Energy.

[69] Deng Y., Chi B., Li J., Wang G., Zheng L., Shi X., Cui Z., Du L., Liao S., Zang K. (2019). Atomic Fe-Doped MOF-Derived Carbon Polyhedrons with High Active-Center Density and Ultra-High Performance toward PEM Fuel Cells. Adv. Energy Mater..

[70] Xu X., Xia Z., Zhang X., Sun R., Sun X., Li H., Wu C., Wang J., Wang S., Sun G. (2019). Atomically Dispersed Fe-N-C Derived from Dual Metal-Organic Frameworks as Efficient Oxygen Reduction Electrocatalysts in Direct Methanol Fuel Cells. Appl. Catal. B Environ..

[71] Zhong R., Wu Y., Liang Z., Guo W., Zhi C., Qu C., Gao S., Zhu B., Zhang H., Zou R. (2019). Fabricating Hierarchically Porous and Fe3C-Embeded Nitrogen-Rich Carbon Nanofibers as Exceptional Electocatalysts for Oxygen Reduction. Carbon.

[72] Deng Y., Tian X., Chi B., Wang Q., Ni W., Gao Y., Liu Z., Luo J., Lin C., Ling L. (2020). Hierarchically Open-Porous Carbon Networks Enriched with Exclusive Fe–Nx Active Sites as Efficient Oxygen Reduction Catalysts towards Acidic H2–O2 PEM Fuel Cell and Alkaline Zn–Air Battery. Chem. Eng. J..

[73] Zheng L., Yu S., Lu X., Fan W., Chi B., Ye Y., Shi X., Zeng J., Li X., Liao S. (2020). Two-Dimensional Bimetallic Zn/Fe-Metal-Organic Framework (MOF)-Derived Porous Carbon Nanosheets with a High Density of Single/Paired Fe Atoms as High-Performance Oxygen Reduction Catalysts. ACS Appl. Mater. Interfaces.

[74] Hou B., Wang C.C., Tang R., Zhang Q., Cui X. (2020). High Performance Fe–N–C Oxygen Reduction Electrocatalysts by Solid-Phase Preparation of Metal–Organic Frameworks. Mater. Res. Express.

[75] Jiao L., Li J., LaRochelle Richard L.K., Stracensky T., Liu E., Sun Q., Sougrati M.-T., Zhao Z., Yang F., Zhong S. (2019). High-Performance Iron-Based ORR Catalysts Synthesized via Chemical Vapor Deposition. ChemRxiv. Preprint..

[76] Thompson S.T., Wilson A.R., Zelenay P., Myers D.J., More K.L., Neyerlin K.C., Papageorgopoulos D. (2018). ElectroCat: DOE’s Approach to PGM-Free Catalyst and Electrode R&D. Solid State Ion..

[77] Huang J.-W., Cheng Q.-Q., Huang Y.-C., Yao H.-C., Zhu H.-B., Yang H. (2019). Highly Efficient Fe–N–C Electrocatalyst for Oxygen Reduction Derived from Core–Shell-Structured Fe(OH)_3_ @Zeolitic Imidazolate Framework. ACS Appl. Energy Mater..

[78] Li Y., Liu X., Zheng L., Shang J., Wan X., Hu R., Guo X., Hong S., Shui J. (2019). Preparation of Fe–N–C Catalysts with FeN _x_ (*x* = 1, 3, 4) Active Sites and Comparison of Their Activities for the Oxygen Reduction Reaction and Performances in Proton Exchange Membrane Fuel Cells. J. Mater. Chem. A.

[79] Al-Zoubi T., Zhou Y., Yin X., Janicek B., Sun C., Schulz C.E., Zhang X., Gewirth A.A., Huang P., Zelenay P. (2020). Preparation of Nonprecious Metal Electrocatalysts for the Reduction of Oxygen Using a Low-Temperature Sacrificial Metal. J. Am. Chem. Soc..

[80] Yang X., Wang Y., Zhang G., Du L., Yang L., Markiewicz M., Choi J., Chenitz R., Sun S. (2020). SiO2-Fe/N/C Catalyst with Enhanced Mass Transport in PEM Fuel Cells. Appl. Catal. B Environ..

[81] Song Y., Zhang X., Cui X., Shi J. (2019). The ORR Kinetics of ZIF-Derived Fe N C Electrocatalysts. J. Catal..

[82] Yang L., Shao Z. (2019). Tunable and Convenient Synthesis of Highly Dispersed Fe–N_x_ Catalysts from Graphene-Supported Zn–Fe-ZIF for Efficient Oxygen Reduction in Acidic Media. RSC Adv..

[83] Guo Z., Zhang Z., Li Z., Dou M., Wang F. (2019). Well-Defined Gradient Fe/Zn Bimetal Organic Framework Cylinders Derived Highly Efficient Iron- and Nitrogen- Codoped Hierarchically Porous Carbon Electrocatalysts towards Oxygen Reduction. Nano Energy.

[84] Chen G., Liu P., Liao Z., Sun F., He Y., Zhong H., Zhang T., Zschech E., Chen M., Wu G. (2020). Zinc-Mediated Template Synthesis of Fe-N-C Electrocatalysts with Densely Accessible Fe-N *_x_* Active Sites for Efficient Oxygen Reduction. Adv. Mater..

[85] Jiao L., Wan G., Zhang R., Zhou H., Yu S.-H., Jiang H.-L. (2018). From Metal–Organic Frameworks to Single-Atom Fe Implanted N-Doped Porous Carbons: Efficient Oxygen Reduction in both Alkaline and Acidic Media. Angew. Chem. Int. Ed..

[86] Jiao L., Zhang R., Wan G., Yang W., Wan X., Zhou H., Shui J., Yu S.-H., Jiang H.-L. (2020). Nanocasting SiO_2_ into Metal–Organic Frameworks Imparts Dual Protection to High-Loading Fe Single-Atom Electrocatalysts. Nat. Commun..

[87] Yu P., Chen R., Zhang Q., Liang S., Ni M., Yang W. (2018). Porous Carbon Supported Atomic Iron as Electrocatalysts for Acidic Oxygen Reduction Reaction. Sci. Bull..

[88] Yang Z.K., Yuan C.-Z., Xu A.-W. (2018). A Rationally Designed Fe-Tetrapyridophenazine Complex: A Promising Precursor to a Single-Atom Fe Catalyst for an Efficient Oxygen Reduction Reaction in High-Power Zn–Air Cells. Nanoscale.

[89] Fujiwara Y., Lee J.-S.M., Tsujimoto M., Kongpatpanich K., Pila T., Iimura K., Tobori N., Kitagawa S., Horike S. (2018). Fabrication of ε-Fe2N Catalytic Sites in Porous Carbons Derived from an Iron–Triazolate Crystal. Chem. Mater..

[90] Meng Z., Cai S., Wang R., Tang H., Song S., Tsiakaras P. (2019). Bimetallic−organic Framework-Derived Hierarchically Porous Co-Zn-N-C as Efficient Catalyst for Acidic Oxygen Reduction Reaction. Appl. Catal. B Environ..

[91] Lai S., Xu L., Liu H., Chen S., Cai R., Zhang L., Theis W., Sun J., Yang D., Zhao X. (2019). Controllable Synthesis of CoN_3_ Catalysts Derived from Co/Zn-ZIF-67 for Electrocatalytic Oxygen Reduction in Acidic Electrolytes. J. Mater. Chem. A.

[92] Chai L., Zhang L., Wang X., Hu Z., Xu Y., Li T.-T., Hu Y., Qian J., Huang S. (2020). Cube-Shaped Metal-Nitrogen–Carbon Derived from Metal-Ammonia Complex-Impregnated Metal-Organic Framework for Highly Efficient Oxygen Reduction Reaction. Carbon.

[93] Wang C.C., Hou B., Wu X., Fan X., Zhang J., Cui X. (2019). Efficient Bimetallic Zeolitic Imidazolate Framework Derived Co–N–C Oxygen Reduction Reaction Electrocatalysts. Mater. Res. Express.

[94] Díaz-Duran A.K., Viva F.A., Roncaroli F. (2019). High Durability Fuel Cell Cathodes Obtained from Cobalt Metal Organic Frameworks. Electrochim. Acta.

[95] Díaz-Duran A.K., Roncaroli F. (2017). MOF Derived Mesoporous Nitrogen Doped Carbons with High Activity towards Oxygen Reduction. Electrochim. Acta.

[96] He Y., Hwang S., Cullen D.A., Uddin M.A., Langhorst L., Li B., Karakalos S., Kropf A.J., Wegener E.C., Sokolowski J. (2019). Highly Active Atomically Dispersed CoN_4_ Fuel Cell Cathode Catalysts Derived from Surfactant-Assisted MOFs: Carbon-Shell Confinement Strategy. Energy Environ. Sci..

[97] Chen J., Yuan X., Lyu F., Zhong Q., Hu H., Pan Q., Zhang Q. (2019). Integrating MXene Nanosheets with Cobalt-Tipped Carbon Nanotubes for an Efficient Oxygen Reduction Reaction. J. Mater. Chem. A.

[98] Bai Y., Yang D., Yang M., Chen H., Liu Y., Li H. (2019). Nitrogen/Cobalt Co-doped Mesoporous Carbon Microspheres Derived from Amorphous Metal-Organic Frameworks as a Catalyst for the Oxygen Reduction Reaction in Both Alkaline and Acidic Electrolytes. ChemElectroChem.

[99] Qiao M., Wang Y., Mamat X., Chen A., Zou G., Li L., Hu G., Zhang S., Hu X., Voiry D. (2020). Rational Design of Hierarchical, Porous, Co-Supported, N-Doped Carbon Architectures as Electrocatalyst for Oxygen Reduction. ChemSusChem.

[100] Zhou H., He D., Saana A.I., Yang J., Wang Z., Zhang J., Liang Q., Yuan S., Zhu J., Mu S. (2018). Mesoporous-Silica Induced Doped Carbon Nanotube Growth from Metal–Organic Frameworks. Nanoscale.

[101] Jose V., Jayakumar A., Lee J. (2019). Bimetal/Metal Oxide Encapsulated in Graphitic Nitrogen Doped Mesoporous Carbon Networks for Enhanced Oxygen Electrocatalysis. ChemElectroChem.

[102] Li G., He X., Yin F., Chen B., Yin H. (2019). Co-Fe/MIL-101(Cr) Hybrid Catalysts: Preparation and Their Electrocatalysis in Oxygen Reduction Reaction. Int. J. Hydrogen Energy.

[103] Wang H., Wei L., Liu J., Shen J. (2020). Hollow Bimetal ZIFs Derived Cu/Co/N Co-Coordinated ORR Electrocatalyst for Microbial Fuel Cells. Int. J. Hydrogen Energy.

[104] Fang H., Huang T., Sun Y., Kang B., Liang D., Yao S., Yu J., Dinesh M.M., Wu S., Lee J.Y. (2019). Metal-Organic Framework-Derived Core-Shell-Structured Nitrogen-Doped CoCx/FeCo@C Hybrid Supported by Reduced Graphene Oxide Sheets as High Performance Bifunctional Electrocatalysts for ORR and OER. J. Catal..

[105] Niu W., Yang Y. (2018). Amorphous MOF Introduced N-Doped Graphene: An Efficient and Versatile Electrocatalyst for Zinc–Air Battery and Water Splitting. ACS Appl. Energy Mater..

[106] Wu M., Hu X., Li C., Li J., Zhou H., Zhang X., Liu R. (2018). Encapsulation of Metal Precursor within ZIFs for Bimetallic N-Doped Carbon Electrocatalyst with Enhanced Oxygen Reduction. Int. J. Hydrogen Energy.

[107] Dong Z., Liu G., Zhou S., Zhang Y., Zhang W., Fan A., Zhang X., Dai X. (2018). Restructured Fe−Mn Alloys Encapsulated by N-Doped Carbon Nanotube Catalysts Derived from Bimetallic MOF for Enhanced Oxygen Reduction Reaction. ChemCatChem.

[108] He T., Ni B., Ou Y., Lin H., Zhang S., Li C., Zhuang J., Hu W., Wang X. (2018). Nanosheet-Assembled Hierarchical Carbon Nanoframeworks Bearing a Multiactive Center for Oxygen Reduction Reaction. Small Methods.

[109] Xia W., Li J., Wang T., Song L., Guo H., Gong H., Jiang C., Gao B., He J. (2018). The Synergistic Effect of Ceria and Co in N-Doped Leaf-like Carbon Nanosheets Derived from a 2D MOF and Their Enhanced Performance in the Oxygen Reduction Reaction. Chem. Commun..

[110] He B., Yuan Y., Wang J., Pervaiz E., Dong X., Shao Z., Yang M. (2018). Hierarchical Ni3ZnN Hollow Microspheres as Stable Non-Noble Metal Electrocatalysts for Oxygen Reduction Reactions. Electrocatalysis.

[111] Ke Q., Yang L., Gao Y., Wu X.-L., Chen F., Liu S., Lin H., Yuan C.-Z., Chen J. (2020). Dual Active Sites of the Co_2_ N and Single-Atom Co–N_4_ Embedded in Nitrogen-Rich Nanocarbons: A Robust Electrocatalyst for Oxygen Reduction Reactions. Nanotechnology.

[112] Wang H., Yin F., Liu N., Kou R., He X., Sun C., Chen B., Liu D., Yin H. (2019). Engineering Fe–Fe_3_ C@Fe–N–C Active Sites and Hybrid Structures from Dual Metal–Organic Frameworks for Oxygen Reduction Reaction in H_2_–O_2_ Fuel Cell and Li–O_2_ Battery. Adv. Funct. Mater..

[113] Ping K., Braschinsky A., Alam M., Bhadoria R., Mikli V., Mere A., Aruväli J., Paiste P., Vlassov S., Kook M. (2020). Fused Hybrid Linkers for Metal–Organic Framework-Derived Bifunctional Oxygen Electrocatalysts. ACS Appl. Energy Mater..

[114] Jin H., Zhou H., He D., Wang Z., Wu Q., Liang Q., Liu S., Mu S. (2019). MOF-Derived 3D Fe-N-S Co-Doped Carbon Matrix/Nanotube Nanocomposites with Advanced Oxygen Reduction Activity and Stability in Both Acidic and Alkaline Media. Appl. Catal. B Environ..

[115] Li S., Jiang Z., Xiao X., Chen W., Tian X., Hao X., Jiang Z.-J. (2019). MOF-Derived Co Nanoparticles Embedded in N,S-Codoped Carbon Layer/MWCNTs for Efficient Oxygen Reduction in Alkaline Media. Ionics.

[116] Luo Y., Zhang J., Chen Y., Li Z., Chen J., Wang G., Wang R. (2019). MOF-Derived Porous Carbon Supported Iron-Based Catalysts with Optimized Active Sites towards Oxygen Reduction Reaction. J. Electroanal. Chem..

[117] Rizvi S.A.M., Iqbal N., Haider M.D., Noor T., Anwar R., Hanif S. (2019). Synthesis and Characterization of Cu-MOF Derived Cu@AC Electrocatalyst for Oxygen Reduction Reaction in PEMFC. Catal. Lett..

[118] Wen X., Qi H., Cheng Y., Zhang Q., Hou C., Guan J. (2020). Cu Nanoparticles Embedded in N-Doped Carbon Materials for Oxygen Reduction Reaction. Chin. J. Chem..

[119] Zheng L., Dong Y., Chi B., Cui Z., Deng Y., Shi X., Du L., Liao S. (2019). UIO-66-NH _2_ -Derived Mesoporous Carbon Catalyst Co-Doped with Fe/N/S as Highly Efficient Cathode Catalyst for PEMFCs. Small.

[120] Luo Y., Zhang J., Kiani M., Chen Y., Chen J., Wang G., Chan S.H., Wang R. (2018). Synthesis of MOF-Derived Nonprecious Catalyst with High Electrocatalytic Activity for Oxygen Reduction Reaction. Ind. Eng. Chem. Res..

[121] Xiao M., Zhang H., Chen Y., Zhu J., Gao L., Jin Z., Ge J., Jiang Z., Chen S., Liu C. (2018). Identification of Binuclear Co2N5 Active Sites for Oxygen Reduction Reaction with More than One Magnitude Higher Activity than Single Atom CoN4 Site. Nano Energy.

[122] Wu Y.-J., Wang Y.-C., Wang R.-X., Zhang P.-F., Yang X.-D., Yang H.-J., Li J.-T., Zhou Y., Zhou Z.-Y., Sun S.-G. (2018). Three-Dimensional Networks of S-Doped Fe/N/C with Hierarchical Porosity for Efficient Oxygen Reduction in Polymer Electrolyte Membrane Fuel Cells. ACS Appl. Mater. Interfaces.

[123] Lv L., Kang S., Li X., Shen J., Liu S. (2018). ZIF-Derived Carbons as Highly Efficient and Stable ORR Catalyst. Nanotechnology.

[124] Gao H., Li Y., Meng E., Ma Y., Zhang Y., Wang L., Luo H., Zhang L., Chen W., Xia Y. (2020). Co/NC-Gr Composite Derived from ZIF-67: Effects of Preparation Method on the Structure and Electrocatalytic Performance for Oxygen Reduction Reaction. Int. J. Hydrogen Energy.

[125] Shijina K., Illathvalappil R., Sumitha N.S., Sailaja G.S., Kurungot S., Nair B.N., Mohamed A.P., Anilkumar G.M., Yamaguchi T., Hareesh U.S. (2018). Melamine Formaldehyde–Metal Organic Gel Interpenetrating Polymer Network Derived Intrinsic Fe–N-Doped Porous Graphitic Carbon Electrocatalysts for Oxygen Reduction Reaction. New J. Chem..

[126] Wang L., Jin X., Fu J., Jiang Q., Xie Y., Huang J., Xu L. (2018). Mesoporous Non-Noble Metal Electrocatalyst Derived from ZIF-67 and Cobalt Porphyrin for the Oxygen Reduction in Alkaline Solution. J. Electroanal. Chem..

[127] Yang L., Zeng Y., Tang X., Xu D., Fang D., Huang H., Shao Z., Yi B. (2018). Self-Sacrificial Template Synthesis of a Nitrogen-Doped Microstructured Carbon Tube as Electrocatalyst for Oxygen Reduction. ChemElectroChem.

[128] Su H., Zhou S., Zhang X., Sun H., Zhang H., Xiao Y., Yu K., Dong Z., Dai X., Huang X. (2018). Metal-Organic Frameworks-Derived Core-Shell Fe3O4/Fe3N@graphite Carbon Nanocomposites as Excellent Non-Precious Metal Electrocatalyst for Oxygen Reduction. Dalton Trans. Camb. Engl. 2003.

[129] Guo D., Han S., Wang J., Zhu Y. (2018). MIL-100-Fe Derived N-Doped Fe/Fe3C@C Electrocatalysts for Efficient Oxygen Reduction Reaction. Appl. Surf. Sci..

[130] Rojas-Carbonell S., Artyushkova K., Serov A., Santoro C., Matanovic I., Atanassov P. (2018). Effect of PH on the Activity of Platinum Group Metal-Free Catalysts in Oxygen Reduction Reaction. ACS Catal..

